# Development and Optimization of Chromosomally-Integrated Fluorescent *Mycobacterium tuberculosis* Reporter Constructs

**DOI:** 10.3389/fmicb.2020.591866

**Published:** 2020-12-09

**Authors:** Katharina Kolbe, Alice C. Bell, Gareth A. Prosser, Maike Assmann, Hee-Jeong Yang, He Eun Forbes, Sophia Gallucci, Katrin D. Mayer-Barber, Helena I. Boshoff, Clifton E. Barry III

**Affiliations:** ^1^Tuberculosis Research Section, Laboratory of Clinical Immunology and Microbiology, National Institute of Allergy and Infectious Diseases, National Institutes of Health, Bethesda, MD, United States; ^2^Drug Discovery Unit, College of Life Sciences, James Black Centre, University of Dundee, Dundee, United Kingdom; ^3^Inflammation and Innate Immunity Unit, Laboratory of Clinical Immunology and Microbiology, National Institute of Allergy and Infectious Diseases, National Institutes of Health, Bethesda, MD, United States

**Keywords:** *Mycobacterium tuberculosis*, fluorescent protein, reporter strain, riboswitch, pH, magnesium

## Abstract

*Mycobacterium tuberculosis* resides in the lungs in various lesion types with unique microenvironmental conditions. This diversity is in line with heterogeneous disease progression and divergent drug efficiency. Fluorescent reporter strains can be used to decipher the micromilieu and to guide future treatment regimens. Current reporters using replicating plasmids, however, are not suitable for long-term mouse infections or studies in non-human primates. Using a combination of recombinant DNA and protein optimization techniques, we have developed reporter strains based on integrative plasmids, which exhibit stimulus-response characteristics and fluorescence intensities comparable to those based on replicating plasmids. We successfully applied the concepts by constructing a multi-color reporter strain able to detect simultaneous changes in environmental pH, Mg^2+^ concentrations, and protein expression levels.

## Introduction

Tuberculosis (TB) is the leading cause of death from a single infectious agent worldwide. In 2019 the World Health Organization (WHO) reported that global TB infections resulted in an estimated 1.4 million fatalities (Global Tuberculosis Report, 2020). *Mycobacterium tuberculosis* (*Mtb*) mainly infects the lungs, where it leads to very heterogeneous lesion types, that possess a wide range of pathological and immunological characteristics. Representative pathologies of pulmonary disease in humans include air-filled cavities and nodules with a wide range of interior composition, including necrotic, fibrotic, and caseous features, often found simultaneously in infected individuals (Lenaerts et al., 2015). The diverse locations where the bacilli reside are accompanied by unique microenvironmental conditions, such as hypoxic stress (Via et al., 2008; Belton et al., 2016; Prosser et al., 2017), nutrient limitation (Kim et al., 2010; Berney and Berney-Meyer, 2017), ion starvation or toxicity (Wagner et al., 2005; Hood and Skaar, 2012; Marcela Rodriguez and Neyrolles, 2014), and low pH (Kempker et al., 2017; Baker et al., 2019). Metabolic and physiologic adaptations of *Mtb* bacteria under these circumstances (Ehrt et al., 2018), as well as the lesion architecture itself (Prideaux et al., 2015; Strydom et al., 2019), strongly influence disease progression and drug efficiency. Given the intricacies of the assorted conditions present during infection and the connection to treatment outcome, it is important to develop tools to probe the local bacterial environment *in vivo* and use this information to guide future treatment regimens.

To date, several fluorescent mycobacterial reporter strains have been successfully applied in mice and have revealed novel insights about the local microenvironment and bacteria-host interactions (Abramovitch, 2018; MacGilvary and Tan, 2018). For instance, Abramovitch et al. (2011) and Tan et al. (2013) developed reporter strains that monitor pH, an environmental cue known to be important for *Mtb in vitro* growth (Supplementary Figure 1A) and intracellular survival (Vandal et al., 2009). While pH is well studied as a critical signal during host infections, the impact of environmental ions has only recently been studied leading to the development of chloride (Cl^–^) and potassium (K^+^)-responsive reporter strains (Tan et al., 2013; MacGilvary et al., 2019). For magnesium (Mg^2+^) ions, however, no tools are currently available to reliably quantify or read out its concentration. The importance of Mg^2+^ for mycobacterial growth has been demonstrated in *in vitro* culture using media with low Mg^2+^ concentration (Supplementary Figure 1B; Buchmeier et al., 2000; Piddington et al., 2000; Walters et al., 2006; Goodsmith et al., 2015) and inhibitors of Mg^2+^ homeostasis (Supplementary Figure 2A; Lopez Quezada et al., 2019; Park et al., 2019). In addition, there is accumulating evidence that survival of *Mtb* within the acidic pH of phagosomes is Mg^2+^ dependent (Buchmeier et al., 2000; Piddington et al., 2000). *Mtb* mutants lacking the persistence-associated integral membrane protein (PerM, *rv0955*) (Goodsmith et al., 2015) or the Mg^2+^ transporter C (MgtC, *rv1811*) (Buchmeier et al., 2000) showed Mg^2+^-dependent growth defects *in vitro*. *In vivo*, these gene deletions resulted in attenuation in the chronic phase of infection and loss of virulence, respectively (Buchmeier et al., 2000; Goodsmith et al., 2015). In this context, it has been hypothesized that Mg^2+^ restriction might be a mechanism to control *Mtb* infections. Thus, there is a need for new reporter strains for this divalent ion.

The drawback of current reporter strains is the use of replicating plasmids, which are not suitable for long term mouse infections or studies in non-human primates due to the loss of the reporter construct over time (our unpublished data; Abramovitch, 2018). Furthermore, relying on a single plasmid limits the number of responsive elements that can be introduced into one reporter strain, thereby restricting the ability to correlate different environmental factors and gain deeper insights into the complexity of the microenvironment.

To overcome these limitations, we have developed novel strategies to generate reporter strains using exclusively integrative plasmids, which exhibit stimulus-response characteristics and fluorescence intensities (FIs) comparable to those based on replicating plasmids. Screening a library of fluorescent proteins (FPs), designing new multicistronic gene constructs, testing various mycobacterial promoters, and exploiting the flexibility of mRNA-based responsive elements gave us the toolbox to accomplish this goal. We successfully applied these concepts by constructing a multi-color reporter strain able to detect simultaneous changes in environmental pH, Mg^2+^ concentrations, and protein expression levels.

## Materials and Methods

### Bacterial Strains, Media, and Culture Conditions

The bacterial strains used in this study are listed in Supplementary Table 1. *Escherichia coli* (*E. coli*) NEB5α strains were cultured in Luria-Bertani liquid media (LB; Sigma) or grown on LB agar (Invitrogen). For selection of recombinant *E. coli*, Carbenicillin (Car), Kanamycin (Kan), and Hygromycin (Hyg) were used at concentrations of 50, 50, and 200 μg/ml, respectively. *Mtb* bacteria were cultured in Difco Middlebrook 7H9 liquid media (BD) supplemented with ADGNTw (Supplementary Table 2) or grown on Difco Middlebrook 7H11 agar (BD) supplemented with OADGN (Supplementary Table 2). For selection of recombinant *Mtb*, Kan and Hyg were used at concentrations of 25 and 50 μg/ml, respectively. For acid and Mg^2+^ stress conditions *Mtb* bacteria were grown in Sauton’s medium (Supplementary Table 2) adjusted to different pH values and supplemented with desired magnesium sulfate (MgSO_4_) concentrations.

### Formation of New Gene Constructs

Cloning vectors, suicide plasmids, and oligonucleotides used in this study are listed in Supplementary Tables 3, 4, respectively. Promoter and gene sequences are listed in Supplementary Tables 5, 6. The plasmids were constructed using oligo annealing, restriction enzyme-based cloning, and Gibson Assembly.

#### Plasmid Modifications

The backbone of plasmid pMV361 (Stover et al., 1991) was amplified using primers 1 and 2, digested with *Nco*I and reannealed, in order to introduce an *Nco*I restriction site at the translational start site and simplify future cloning steps. The L5 integrase was removed using primers 3 and 4 with terminal *Bam*HI restriction sites. Furthermore, the *hsp60* promoter (Stover et al., 1991) was exchanged with P_s__myc_ from pML1357 (Huff et al., 2010) exploiting the *Xba*I and *Nco*I restriction sites. The new plasmid was named pL5 P_smyc_ (Supplementary Figure 3).

pML1357, an integrative plasmid targeted to the mycobacterial Giles integration site (Huff et al., 2010), was purchased from Addgene and modified by exchanging the multiple cloning site and *smyc* promoter with those from pL5 P_smyc_ using *Xba*I and *Hin*dIII restriction sites. The Giles integrase of pML1357 was removed by PCR amplification of the plasmid using primers 7 and 8 with *Xho*I restriction sites and reannealing to form pML1357dI. Primers 9 and 10 were used to introduce a second multiple cloning site into pML1357dI replacing the *xylE* gene encoding catechol 2,3 dioxygenase. The new plasmid was named pGiles P_smyc_ (Supplementary Figure 3).

In order to transfer the Giles integrase into a suicide vector, the gene for the integrase together with its promoter was amplified from pML1357 using primers 11 and 12 and inserted into the non-mycobacterial plasmid pUC19 (Norrander et al., 1983). The plasmid was named pUC19-GI.

#### Library of FP Genes

To enable quantification of protein expression levels via western blot, an *N*-terminal 6x-His tag was added to all FPs used in this study. A DNA sequence encoding the 6x-His tag was introduced downstream of the *smyc* promoter (Kaps et al., 2001; Ehrt et al., 2005) in the pL5 P_smyc_ plasmid via oligo annealing using oligomers 13 and 14. FP genes, purchased from Addgene (Supplementary Table 3), were amplified using the respective primers in Supplementary Table 4 and introduced by restriction enzyme-based cloning, usually *Nco*I and *Hin*dIII, downstream of the P_s__myc_-*His* element.

#### Multicistronic Constructs

The Addgene-sourced *mWasabi* gene contained an internal *Nco*I site. To enable *Nco*I-based cloning, the restriction site was removed by a single point mutation (C_354_ → G_354_) using Site-Directed Mutagenesis. The modified *mWasabi dN* gene was subsequently cloned downstream of the P_s__myc_-*His* element using *Nco*I and *Hin*dIII restriction sites. In addition, a synthetic, codon-optimized version of *mWasabi* was purchased (Eurofins) based on reported mycobacterial codon preferences.^1^ The *COmWasabi* synthetic gene was introduced into the *Nco*I and *Cla*I sites downstream of P_s__myc_-*His* with primer pair 41 and 42 containing the restriction sites *Bsp*HI and *Cla*I. *Nco*I and *Bsp*HI ligation led to destruction of the *Nco*I site. *His*-tagged *COmWasabi* together with the ribosome binding site (RBS) was then amplified using primers 43 and 42 and cloned into the restriction sites *Hin*dIII and *Cla*I downstream of *mWasabi dN*. The new gene construct was named double green and could be easily transferred using *Nco*I- and *Cla*I-based cloning.

In order to generate the double red construct, the *mScarlet* gene was codon optimized and cloned downstream of the P_s__myc_-*His* element using primers 21 and 44 with the restriction sites *Nco*I and *Hin*dIII. Subsequently, *His*-tagged *COmScarlet* together with its RBS was transferred into the *Hin*dIII restriction site at the 3′ end of the *mScarlet* gene using primer pair 45 and 44. For the triple red construct, an RBS and the *His*-tagged gene encoding mRuby3 was introduced downstream of the two *mScarlet* genes by exploiting the *Cla*I restriction site. Since the *RBS*-*HismRuby3* DNA element was amplified with a forward primer containing an *Acl*I restriction site, the *Cla*I site at the 5′ end was deleted while it remained intact at the 3′ end.

#### Promoter Library

For the promoter library, the *smyc* promoter upstream of *eGFP* was exchanged with the promoters P_h__sp__60_, P_G__13_ (Barker et al., 1999), P_m__sp__12_ (Chan et al., 2002), P_MOP_ (George et al., 1995), and P_left_ (Nesbit et al., 1995), respectively. Plasmids containing P_h__sp__60_, P_G__13_, and P_m__sp__12_ were purchased from Addgene. P_MOP_ was obtained from the vector pMH29 (George et al., 1995) and a synthetic DNA sequence of P_left_, identical to the sequence described by Jain et al. (2012), but with an *Nco*I site at the 3′ end, was ordered from Eurofins. The promoters P_h__sp__60_, P_G__13_, P_m__sp__12_, and P_left_ were introduced into the *Xba*I and *Nco*I restriction sites upstream of the *eGFP* gene. P_MOP_ was inserted into the same restriction sites, but using primer pair 52 and 53 containing *Spe*I and *Nco*I sites.

The P_left^*^_ element was constructed by Gibson Assembly ligating the PCR product of P_left_, amplified using primers 56 and 57, and the *Xba*I/*Bsa*HI digested pL5 P_smyc_-*eGFP* plasmid. We used the same oligo annealing strategy, described above, to introduce a 6x-*His* tag downstream of the P_left^*^_ element.

#### Mono-Responsive Reporter Gene Constructs

pGiles was used as the backbone for constructing the pH-responsive reporter plasmid. The triple red gene construct, reported above, was amplified from the respective pL5 P_smyc_-triple red plasmid and transferred into the second multiple cloning site of pGiles carrying the native promoter of *rv2390c* (Tan et al., 2013) in the restriction sites *Bmt*I and *Bgl*II. To accomplish this insertion, we applied Gibson Assembly. The plasmid was digested with the enzymes *Bgl*II and *Not*I and the three genes were amplified together using primers 58 and 59. These primers contained *Apa*I and *Bgl*II intrinsic restriction sites, respectively, which led to a new *Bgl*II site downstream of the triple red element and an *Apa*I site at the 5′ end. Subsequently, using the restriction sites *Bmt*I and *Apa*I the native promoter of *rv2390c* was exchanged with a 6x-*His*-tagged native promoter of the mycobacterial gene *rv2395A* (Abramovitch et al., 2011) amplified from *Mtb* H37Rv genomic DNA.

The Mg^2+^-responsive elements together with their native promoters upstream of the mycobacterial genes *rv1535* and *rv1806* (P_np__1535_ and P_np__1806_) were amplified from *Mtb* genomic DNA using primers 62–65. These DNA fragments were cloned into the first multiple cloning site of pGiles exploiting the restriction sites *Xba*I and *Nco*I. A 6x-*His* tag was inserted into the *Nco*I site by oligo annealing. *mWasabi dN* was introduced downstream between *Nco*I and *Hin*dIII sites, using primers 39 and 40.

In order to construct the P_s__myc__1806_ and P_left__1806_ fusion products, we applied Gibson Assembly. As vectors, pL5 containing either P_s__myc_-*mWasabi dN* or P_left__^*^_-*HismClover3* were used. The plasmid backbone was amplified with primers 66 and 67 or 70 and 71. The Mg^2+^-responsive element was amplified from P_np__1806_-*mWasabi dN* with primers 68, 69, 72, and 73 and ligated with the respective plasmid part. Downstream of P_s__myc__1806_ a 6x-*His* tag was introduced as described above. Subsequently, the fused elements were transferred into the *Xba*I and *Nco*I restriction sites upstream of *mWasabi dN* in the pGiles plasmid.

For the Mg^2+^ responsive double green constructs, the *His*-tagged gene encoding COmWasabi together with its RBS was introduced downstream of the *mWasabi dN* gene, as described above.

#### Tri-Responsive Reporter Gene Construct

To reduce the number of plasmids needed for the construction of the *Mtb* tri-responsive reporter strain, the DNA regions encoding P_np__2395__A_-triple red and P_left__1806_-double green were combined in a single plasmid. The original plasmids containing these reporter constructs were digested with *Xba*I and *Cla*I and newly ligated, introducing P_left__1806_-double green into the vector containing the pH-responsive element.

The double blue system under the control of the constitutive promoter P_s__myc_ was designed as follows. The gene of mTagBFP2 was codon optimized and introduced into the *Nco*I and *Cla*I restriction sites, downstream of the P_s__myc_-*His* element in pL5 P_smyc_-*His*, using primer pair 74 and 75 with *Bsp*HI and *Cla*I sites, respectively, thus, deleting the *Nco*I cloning site. The DNA region encoding His-tagged COmTagBFP2 together with the RBS was amplified using primers 43 and 75 and cloned downstream of P_s__myc_-*HismTagBFP2* in the pL5 P_smyc_-*HismTagBFP2* plasmid.

### Generation of Fluorescent *Mtb* Strains and Reporter Strains

Plasmids were incorporated into electrocompetent *Mtb* H37Rv (or HN878 for *in vivo* assay) cells via electroporation using a Bio-Rad GenePulser Xcell electroporator and 2 mm electroporation cuvettes (Bio-Rad). The conditions for electroporation were 2500 V, 25 μF, and 1000 Ω. All pL5-based plasmids were co-electroporated with a suicide plasmid, pBS-Int (Springer et al., 2001), which encodes for the L5 integrase. The suicide plasmid used for incorporation of pGiles plasmids was pUC19-GI. Transformants were selected on 7H11 agar (BD) containing Kan, Hyg, or both antibiotics.

### Western Blot

*Mtb* H37Rv bacteria expressing the FP constructs were grown in 7H9 ADGNTw media (20 mL) to an OD_600_ of 0.6. Cultures were centrifuged (3000 × *g*, RT, 10 min) and resuspended in lysis buffer (Thermo Fisher Scientific; 600 μl) containing 1× protease inhibitor (Roche). Bacterial lysis was performed by bead-beating (Roche MagnaLyzer; 6500 rpm, 45 s, three times with cooling on ice in between) with 0.1 mm Zirconia/Silica beads (BioSpec Products; 400 μl). Lysates were centrifuged (21,130 × *g*, 4°C, 5 min) and protein concentrations of the supernatants determined by BCA assay (Pierce). Samples were then boiled (95°C, 5 min) with reducing loading SDS-PAGE sample buffer (Bio-Rad) and equivalent protein quantities of each sample loaded onto Any kD Mini-PROTEAN TGX Precast Protein Gel (Bio-Rad; 220 V, 35 min) with a Color Prestained Protein Standard (Broad Range, 10–250 kDa, NEB). Proteins were transferred onto a nitrocellulose membrane (Invitrogen) using the Trans-Blot Turbo Transfer System (Bio-Rad; 25 V, 10 min). The membrane was blocked with BSA-TBST [5% BSA in TBST (1× TBS with 0.1% Tween 20)] for 1 h at RT, washed three times (15 min each) with TBST, cut in half, and incubated with the primary mouse antibodies anti-His6 (Sigma; 1:3000 dilution in BSA-TBST) or anti-GroEL (Abcam, ab20519; 1:2000 dilution in BSA-TBST) overnight at 4°C. After three washing steps for 15 min, each with TBST, the secondary goat anti-mouse horseradish peroxidase (HRP) antibody (Invitrogen; 1:10,000 dilution in BSA-TBST) was applied and the membrane was incubated for 1 h at RT. The blot was washed three times (15 min each) with TBST, developed using Clarity and Clarity Max ECL Western Blotting Substrate (Bio-Rad), and analyzed with the ChemiDoc Imaging System (Bio-Rad). Relative quantifications of protein expression levels were carried out using the Bio-Rad Image Lab Software.

### *In vitro* Assays

#### Measuring Signal Intensities of Fluorescent *Mtb* Strains

The various H37Rv strains expressing the FP constructs were grown to mid-logarithmic phase in 7H9 ADGNTw media containing the required antibiotic. A total of 200 μl each were transferred into a clear-bottomed black 96 well plate (Costar) and the plate was placed in a CLARIOstar Plus microplate reader (BMG LABTECH). Fluorescence measurements were taken using the optimal excitation and emission wavelengths of each FP and values were normalized to the respective optical densities (OD_600_) of culture in each well.

#### Detection of FI Changes Based on Environmental Conditions

Reporter strains, grown in 7H9 ADGNTw medium, were adapted for one week to Sauton’s medium (pH 7.0, 500 μM MgSO_4_) starting with an initial OD_600_ of 0.01. Subsequently, 1 ml of culture (OD_600_: 0.8) was centrifuged (20,293 × *g*, RT, 2 min), the pellet washed once with Sauton’s medium without MgSO_4_, and finally resuspended in 800 μl of the same Sauton’s medium (pH 7.0, no MgSO_4_) leading to an OD_600_ of 1.0. A total of 24 well plates were assembled with Sauton’s media containing the required antibiotic, pH values and Mg^2+^ concentrations (1 ml/well), and the reporter strain suspension was added. Starting ODs were adjusted to the bacterial growth characteristics in the respective medium, e.g., for a 4 days time point the OD_600_ in medium with a pH of 7.0 and 500 μM MgSO_4_ was set to 0.025, while OD_600_s in a media with low pH (5.0) or low Mg^2+^ concentrations (10 μM) were set to 0.1. After the respective incubation time at 37°C, 200 μl of each well was transferred to a clear-bottomed black 96 well plate (Costar) and FIs determined as described above. The same experimental set up was applied to analyze the impact of other divalent ions (Ca^2+^, Co^2+^, Cu^2+^, Mn^2+^, Ni^2+^, Zn^2+^) or the CorA inhibitor pyrimidinetrione amide analog 10 (PAA10) (Park et al., 2019) on riboswitch-based fluorescence response. In the case of the divalent ions, a MgSO_4_ concentration of 10 μM was used and a divalent ion concentration around fourfold lower than the determined minimal inhibitory concentrations (MICs). The influence of PAA10 was tested using six different concentrations (0, 5, 25, 50, 100, 250 nM).

#### Determination of Minimal Inhibitory Concentrations

The various divalent ions (Ca^2+^, Co^2+^, Cu^2+^, Mn^2+^, Ni^2+^, Zn^2+^) and the molecule PAA10 were serially (1:2) diluted in Sauton’s medium (pH 7.0, 500 μM MgSO_4_) in 96-well round-bottom plates (Thermo Fisher Scientific; 50 μl/well). The starting concentrations of the divalent ions and PAA10 were 5 mM and 4 μM, respectively. *Mtb* H37Rv wild-type (WT) bacteria were grown in Sauton’s medium (pH 7.0, 500 μM MgSO_4_) to an OD_600_ of 0.2. The culture was diluted (1:1000) in the same medium and 50 μl was added to each well. Plates were analyzed after 14 days of incubation at 37°C. The MIC was determined as the lowest concentration of the tested substance that showed complete inhibition of visible bacterial growth.

### *In vivo* Assay

#### *In vivo* Detection of P_left^*^_ FP Strains by Flow Cytometry

For *in vivo* infections, WT B6.SJL (CD45.1/1) mice were intrapharyngeally inoculated with 50 CFU of *Mtb* HN878 (WT, L5 *attB*:P_left^*^_ *mWasabi*, L5 *attB*:P_left^*^_ *mCherry*, or L5 *attB*:P_left^*^_ *mScarlet*). Lungs were harvested 37 days post-infection and dissociated via GentleMACS and Lung Cell Isolation Buffer (Miltenyi Biotec). Digested lungs were passed through a 100 μm cell strainer and cells washed and purified with 37% Percoll/RPMI. Cells for flow cytometry were washed, counted and subsequently fixed overnight using an Intracellular Fixation & Permeabilization Buffer (eBioscience/Thermo Fisher Scientific). Samples were acquired on a X50 Symphony flow cytometer (BD Biosciences) and analyzed using FlowJo software (BD Biosciences).

#### *In vivo* Detection of *Mtb* HN878 (L5 *attB*:P_left*_ *mScarlet*) by Confocal Microscopy

C3HeB/FeJ mice were infected with *Mtb* HN878 (L5 *attB*:P_left^*^_ *mScarlet*) by aerosol (50–100 CFU/lung). Mice were treated with standard TB drugs at 5 weeks post-infection for 4 weeks and followed up for 4 weeks prior to disease reactivation. At 13 weeks post-infection, lungs were harvested after perfusion with 4% paraformaldehyde and incubated in a fixation and permeabilization solution (BD Biosciences) for overnight at 4°C followed by washing and dehydration in 30% sucrose. Lungs were embedded in OCT compound (Sakura) and stored at −80°C until used. A total of 20 μm sections of frozen lung tissue were incubated in a blocking buffer (1% BSA, 0.3% Triton X-100, 1% Fc block in 1× PBS) for 2 h at RT followed by staining with anti-mouse CD68 antibody (FA11; Biolegend) overnight at 4°C. Tissues were imaged on a Leica SP8 confocal microscope and images were analyzed with Imaris software (Bitplane).

## Results

**FIGURE 1:**
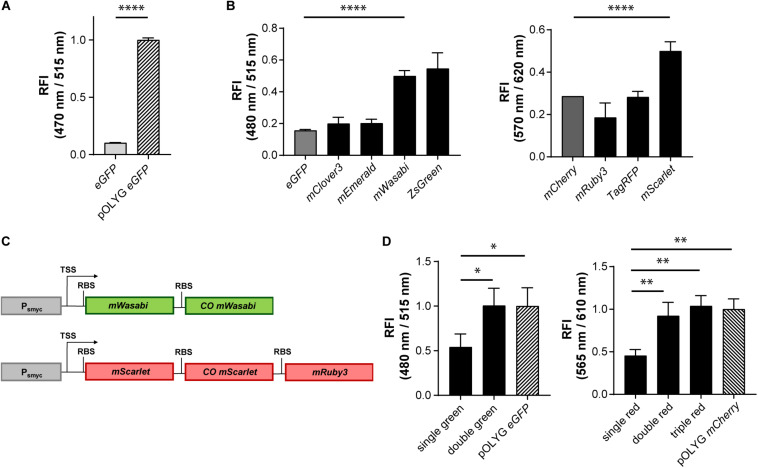
Increase in relative fluorescence intensity using different fluorescent proteins and multicistronic constructs. **(A)** Relative fluorescence intensity (RFI) of *Mtb* H37Rv strains expressing eGFP either from an integrated gene construct or from the replicating plasmid pOLYG, and **(B)** *Mtb* H37Rv strains expressing different green (left) or red (right) fluorescent proteins. **(C)** Design of double green and triple red gene constructs. TSS indicates transcriptional start site; RBS indicates ribosome binding site. **(D)** RFIs of *Mtb* H37Rv strains containing the double or triple gene constructs chromosomally integrated compared to the replicating plasmids pOLYG P_smyc_-*eGFP* and pOLYG P_smyc_-*mCherry*, respectively. All genes were under the control of the *smyc* promoter. FIs were detected during the logarithmic growth phase of the strains, analyzed relative to their optical densities at 600 nm, and normalized with FIs of *Mtb* (pOLYG P_smyc_ *eGFP*) and *Mtb* (pOLYG P_smyc_ *mCherry*) equal to 1. Data are representative of at least three independent experiments; the error bars indicate standard deviation; for statistical analysis groups were compared by Student’s unpaired, two-tailed *t*-tests, *p*-values: ^****^p < 0.0001, ^∗∗^*p* < 0.01, ^∗^*p* < 0.05.

We sought to develop fluorescent reporter strains based on stable integrative plasmids that would be detectable *in vivo* at equivalent bacterial densities as those achieved currently with episomal, multi-copy constructs. To determine the brightness difference between episomal multicopy and chromosomal single copy expression, we first measured the signal intensity of mycobacteria expressing eGFP either from the replicating plasmid pOLYG or from a gene construct integrated into the L5 *attB* site of the mycobacterial chromosome, *in vitro*. In both strains, expression of the FP was under control of the same mycobacterial promoter, P_s__myc_. The FI of *Mtb* (pOLYG P_smyc_ *eGFP*) was approximately 10 times higher than that of *Mtb* (L5 *attB*:P_smyc_ *eGFP*), carrying a single copy of the gene (Figure 1A).

To increase the brightness of *Mtb* strains carrying the gene construct chromosomally, we screened a library of FPs with excitation and emission wavelengths comparable to those of the commonly used green and red FPs eGFP and mCherry, constructed multicistronic FP constructs, and tested expression strengths of various mycobacterial promoters.

### Increase of Signal Intensity Using Different FPs

Genes encoding five different green FPs (eGFP, mClover3, mEmerald, mWasabi, ZsGreen) and four red FPs (mCherry, mRuby3, TagRFP, mScarlet) were cloned downstream of the mycobacterial *smyc* promoter within an integrative plasmid. *Mtb* bacteria were transformed with these constructs and FIs relative to the OD_600_s of the strains were measured. All mycobacterial strains expressing alternative green FPs showed higher FIs compared to the eGFP expressing reference strain. The brightest signals were observed for *Mtb* (L5 *attB*:P_smyc_ *mWasabi*) and *Mtb* (L5 *attB*:P_smyc_ *ZsGreen*) (Figure 1B) with a threefold increase above that of *Mtb* (L5 *attB*:P_smyc_ *eGFP*). For *Mtb* (L5 *attB*:P_smyc_ *mWasabi*) a comparable 3.5 times higher protein expression level was detected by western blot. In contrast, *Mtb* (L5 *attB*:P_smyc_ *ZsGreen*) had a similar expression level to that of *Mtb* (L5 *attB*:P_smyc_ *eGFP*) (Supplementary Figure 4A). In the group of red-fluorescent *Mtb* strains, only the one expressing mScarlet showed a significantly higher FI compared to the mCherry expressing strain (Figure 1B). Conversely, the protein expression level of *Mtb* (L5 *attB*:P_smyc_ *mScarlet*) was 10-fold lower than *Mtb* (L5 *attB*:P_smyc_ *mCherry*) as quantified by western blot (Supplementary Figure 4B).

### Increase of Signal Intensity Using Multicistronic FP Constructs

Introducing different FPs resulted in an increase in FI of the stably integrated single copy constructs, however brightness levels were still significantly below those of *Mtb* (pOLYG P_smyc_ *eGFP*) and *Mtb* (pOLYG P_smyc_ *mCherry*). To enhance the FI of the single copy *mWasabi* construct, a second, codon optimized, allele encoding mWasabi, along with an RBS, was cloned downstream of the P_s__myc_-gene region. Codon optimization was performed in order to increase expression levels as well as limit homologous recombination and thus the loss of gene constructs over time. A similar bicistronic gene sequence was designed for the red FPs including *mScarlet* and codon optimized *mScarlet* (*COmScarlet*). Furthermore, a tricistronic construct was developed containing the gene for mRuby3 downstream of the two *mScarlet* genes (Figure 1C). The new plasmids were transformed into *Mtb* resulting in the strains *Mtb* (L5 *attB*:P_smyc_ double green), *Mtb* (L5 *attB*:P_smyc_ double red), and *Mtb* (L5 *attB*:P_smyc_ triple red). Comparison of the FIs showed a twofold signal increase for *Mtb* (L5 *attB*:P_smyc_ double green) as well as *Mtb* (L5 *attB*:P_smyc_ double red) compared to the equivalent *Mtb* strain containing only one gene of the FP. The fluorescence readout signal of *Mtb* (L5 *attB*:P_smyc_ double green) was equivalent to that achieved by *Mtb* (pOLYG P_smyc_ *eGFP*). *Mtb* (L5 *attB*:P_smyc_ triple red) showed a slightly higher FI than *Mtb* (L5 *attB*:P_smyc_ double red), which was comparable to that of *Mtb* (pOLYG P_smyc_ *mCherry*) (Figure 1D).

### Increase of Signal Intensity Using Different Mycobacterial Promoters

**FIGURE 2:**
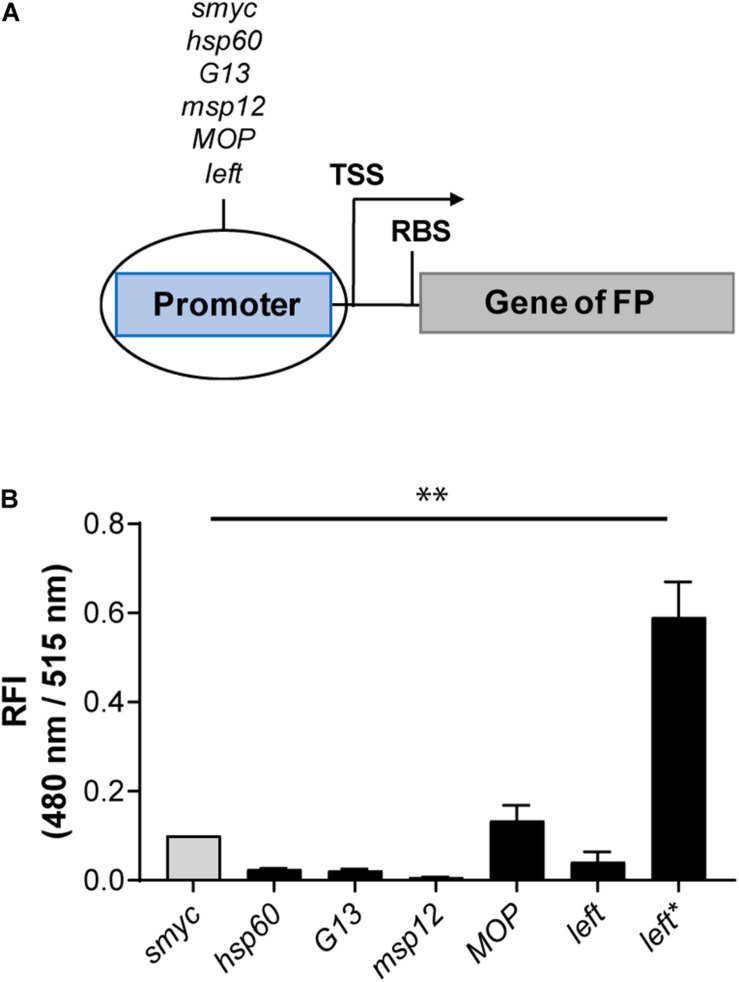
Introduction of the P_left*_ element led to a strong fluorescence increase. **(A)** Design of the promoter-gene constructs. TSS indicates transcriptional start site; RBS indicates ribosome binding site. **(B)** RFI of *Mtb* H37Rv strains expressing eGFP under the control of different constitutive promoters. All gene constructs were chromosomally integrated. FIs were detected during the logarithmic growth phase of the strains, analyzed relative to their optical densities at 600 nm, and normalized with FI of *Mtb* (L5 *attB*:P_smyc_ *eGFP*) equal to 0.1. Data are representative of at least two independent experiments; the error bars indicate standard deviation; for statistical analysis groups were compared by Student’s unpaired, two-tailed *t*-tests, *p*-value: ^∗∗^*p* < 0.01.

As an alternative strategy to increase FIs, we cloned a variety of mycobacterial promoters (P_s__myc_, P_h__sp__60_, P_G__13_, P_m__sp__12_, P_MOP_, and P_left_) upstream of the gene encoding eGFP in a chromosomally integrating plasmid (Figure 2A). The strongest eGFP expression level was detected for *Mtb* (L5 *attB*:P_MOP_ *eGFP*), with a FI fivefold higher compared to the *Mtb* strain containing the commonly used *hsp60* promoter and less than 1.5 times brighter compared to *Mtb* (L5 *attB*:P_s__myc_ *eGFP*). The lowest fluorescence signal was measured for *Mtb* (L5 *attB*:P_m__sp__12_ *eGFP*). *Mtb* (L5 *attB*:P_h__sp__60_ *eGFP*), *Mtb* (L5 *attB*:P_G__13_ *eGFP*), and *Mtb* (L5 *attB*:P_left_ *eGFP*) had similar brightness levels. Analysis of the P_left_ and P_s__myc_ promoter-RBS sequences (Supplementary Table 5) revealed that P_s__myc_ contained an RBS (AGGAGG) similar to the consensus RBS of *Mtb* (DeJesus et al., 2013; Newton-Foot and Gey van Pittius, 2013), while the putative RBS for P_left_ was identified as GGGAGA. Under the assumption that the consensus RBS is associated with stronger expression levels, we exchanged the RBS downstream of the *left* promoter to that of P_s__myc_ to generate a new promoter-RBS element, which was named P_left^*^_. P_left^*^_ was cloned upstream of the gene encoding eGFP and subsequently transformed into *Mtb*. When the FI of *Mtb* (L5 *attB*:P_left^*^_ *eGFP*) was compared to the other mycobacterial promoters previously tested, there was a 25-fold increase in expression strength compared to *Mtb* (L5 *attB*:P_h__sp__60_ *eGFP*) and a sixfold increase in expression over the P_s__myc_ containing *Mtb* strain (Figure 2B).

### Combination of Bright Protein Candidates and the New P_left^*^_ Construct

**FIGURE 3:**
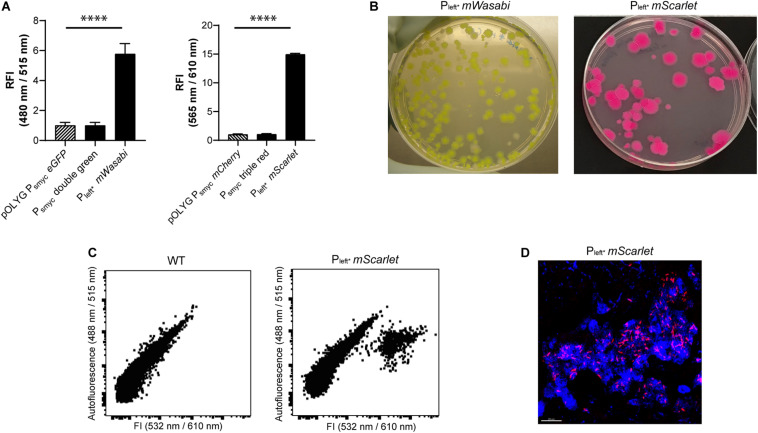
Fluorescence intensities were strongly increased when combining the P_left*_ element with genes expressing mWasabi or mScarlet. **(A)** Fluorescence comparison between *Mtb* H37Rv strains containing either the replicating plasmids pOLYG P_smyc_-*eGFP* and pOLYG P_smyc_-*mCherry*, respectively, or the chromosomally integrated gene constructs P_smyc_-double green/-triple red, and P_left*_-*HismWasabi*/-*HismScarlet*. RFI were determined during the logarithmic growth phase of the strains, analyzed relative to their optical densities at 600 nm, and normalized with FIs of *Mtb* (pOLYG P_smyc_ *eGFP*) and *Mtb* (pOLYG P_smyc_ *mCherry*) equal to 1. Data are representative of at least two independent experiments; the error bars indicate standard deviation; for statistical analysis groups were compared by Student’s unpaired, two-tailed *t*-tests, *p*-values: ^****^p < 0.0001. **(B)** *Mtb* (L5 *attB*:P_left*_ *mWasabi*) and *Mtb* (L5 *attB*:P_left*_ *mScarlet*) colonies grown on 7H11 agar. **(C)** Flow cytometric analysis of *Mtb* HN878 (L5 *attB*:P_left*_ *mScarlet*) at 610 nm in lung single-cell suspensions from WT B6.SJL (CD45.1/1) 37 days post-infection with 50 CFU. Data are representative of one experiment with three mice. **(D)** Lung tissue image of C3HeB/FeJ mice 13 weeks post-infection with *Mtb* HN878 (L5 *attB*:P_left*_ *mScarlet*). mScarlet expressing bacilli (red) were detected intracellularly in macrophages (CD68+, blue). Scale bar indicates 20 μm.

After identification of P_left^*^_ as a strong promoter-RBS element, we combined it with the alternative green and red FP candidates described previously. P_left^*^_ was cloned upstream of the genes encoding mWasabi and mScarlet in chromosomally-integrating plasmids, and transformed into *Mtb*. *Mtb* (L5 *attB*:P_left^*^_ *mWasabi*) showed fivefold higher FI compared to the *Mtb* (L5 *attB*:P_s__myc_ double green), and *Mtb* (pOLYG P_s__myc_ *eGFP*) strains. The signal intensity of the *Mtb* (L5 *attB*:P_left^*^_ *mScarlet*) strain was about 15 times higher than the *Mtb* (L5 *attB*:P_s__myc_ triple red), and the *Mtb* (pOLYG P_s__myc_ *mCherry*) strains (Figure 3A). In addition, *Mtb* (L5 *attB*:P_left^*^_ *mWasabi*) and *Mtb* (L5 *attB*:P_left^*^_ *mScarlet*) colonies appear visibly green and pink, respectively, when grown on agar plates (Figure 3B). Additional agar plate images of *Mtb* strains expressing other FPs under the control of the *left*^∗^ promoter are depicted in Supplementary Figure 5. Despite their brightness, *Mtb* (L5 *attB*:P_left^*^_ *mScarlet*) had very similar growth characteristics and *Mtb* (L5 *attB*:P_left^*^_ *mWasabi*) only minimal growth differences on agar plates and in liquid media (Supplementary Figure 6) in comparison to a WT strain.

### Detection of P_left^*^_ FP Strains in Lungs of WT Mice

The new, bright P_left^*^_ FP strains described above represent a useful starting point for the construction of more advanced reporter strains. Based on their strong fluorescence profile, these strains also provide an attractive alternative to commonly used fluorescent *Mtb* strains for *in vivo* imaging. This was tested by infecting WT B6.SJL (CD45.1/1) mice with *Mtb* HN878 (WT), *Mtb* HN878 (L5 *attB*:P_left^*^_ *mCherry*), *Mtb* HN878 (L5 *attB*:P_left^*^_ *mScarlet*), and *Mtb* HN878 (L5 *attB*:P_left^*^_ *mWasabi*) and analyzing single-cell suspensions of mouse lungs after fixation 5 weeks post infection by flow cytometry. Cells infected with the fluorescent P_left^*^_ strains could clearly be distinguished from uninfected cells (Figure 3C and Supplementary Figure 7). In addition, *Mtb* HN878 (L5 *attB*:P_left^*^_ *mScarlet*) bacteria were successfully visualized in lungs of C3HeB/FeJ mice 13 weeks post-infection using confocal microscopy (Figure 3D), indicating long-term expression of the FP during mouse infections.

### Exploiting Multicistronic FP Constructs to Generate a Bright pH Reporter

**FIGURE 4:**
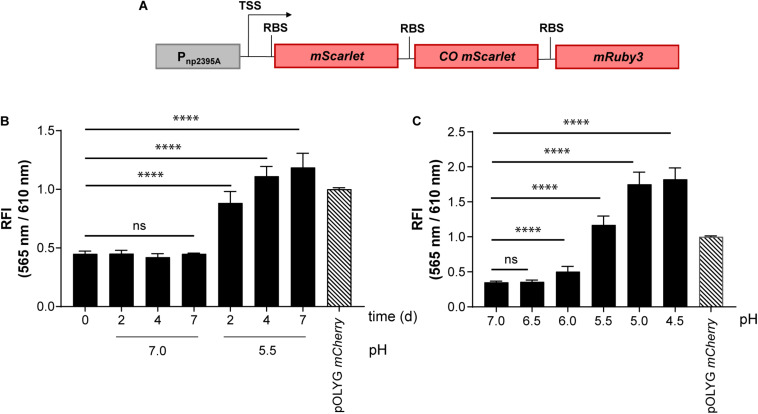
*Mtb* (Giles *attB*:P_np__2395__A_ triple red) showed pH-dependent fluorescence with high signal intensity despite chromosomal integration. **(A)** Design of the pH-responsive gene construct. TSS indicates transcriptional start site; RBS indicates ribosome binding site. RFI of *Mtb* (Giles *attB*:P_np__2395__A_ triple red) was analyzed as a function of **(B)** time and **(C)** pH dependency at a Mg^2+^ concentration of 500 μM after 6 days of incubation. FIs were detected during the logarithmic growth phase of the strain, analyzed relative to its optical densities at 600 nm, and normalized with FI of *Mtb* (pOLYG P_smyc_ *mCherry*) equal to 1. Data are representative of at least three biological replicates; the error bars indicate standard deviation; for statistical analysis groups were compared by Student’s unpaired, two-tailed *t*-tests, *p*-values: ^****^p < 0.0001, ns: not significant.

To apply these new findings to the optimization of more complex, chromosomally-integrated reporter constructs, we first exchanged the *smyc* promoter in the triple red gene construct for the previously reported pH-responsive promoter of the *Mtb* gene *rv2395A* (Abramovitch et al., 2011; Figure 4A). After successful transformation of *Mtb*, FIs of the new reporter strain *Mtb* (Giles *attB*:P_np__2395__A_ triple red) were measured *in vitro* as a function of pH and time. In agreement with the results described previously using a multi-copy reporter construct (Abramovitch et al., 2011), our multicistronic, integrated, pH reporter displayed a threefold increase in fluorescence after 3 days incubation at acidic pH (5.5) compared to neutral pH (7.0). Importantly, the detected fluorescence readout at pH 5.5 reached the level of *Mtb* (pOLYG P_smyc_ *mCherry*) (Figure 4B). We further investigated the reporter’s response under a range of physiologically relevant pH values. While no significant difference in FI was detected at pH 6.5 compared to pH 7.0, a pH-dependent increase was apparent from pH 6.0 to pH 4.5, with signal intensities twofold higher at pH 5.0 and 4.5 compared to the reference strain *Mtb* (pOLYG P_smyc_ *mCherry*) (Figure 4C and Supplementary Figure 8).

### Generation of Novel Mg^2+^ Reporter Strains Using mRNA-Based Responsive Elements

**FIGURE 5:**
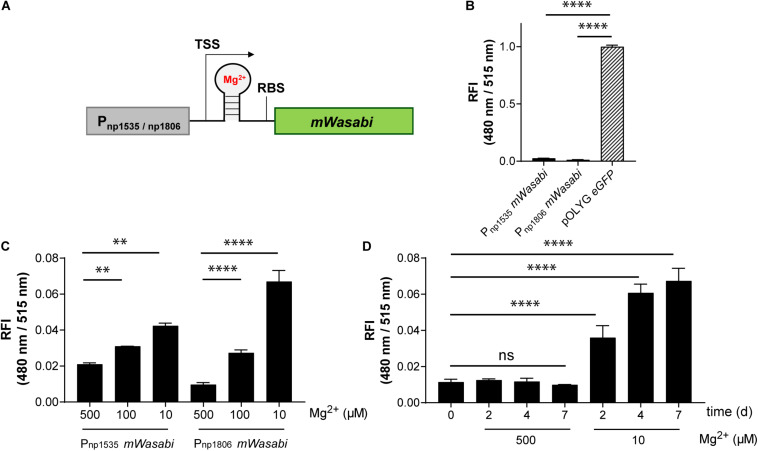
*Mtb* reporter strains carrying the native promoter regions upstream of the genes *rv1535* or *rv1806* showed Mg^2+^-dependent readout signals, but with low fluorescence intensities. **(A)** Design of Mg^2+^-responsive gene constructs. TSS indicates transcriptional start site; RBS indicates ribosome binding site. **(B)** Fluorescence comparison between *Mtb* bacteria expressing eGFP from the replicating plasmid pOLYG and the reporter strains *Mtb* (Giles *attB*:P_np__1535_ *mWasabi*) and *Mtb* (Giles *attB*:P_np__1806_ *mWasabi*) at a Mg^2+^ concentration of 500 μM and pH 7.0. **(C)** Mg^2+^ dependence of the RFI of the reporter strains at pH 7.0 after 7 days of incubation. **(D)** Time dependency of the fluorescence increase of *Mtb* (Giles *attB*:P_np__1806_ *mWasabi*) when exposed to low Mg^2+^ levels (10 μM) at pH 7.0. FIs were detected during the logarithmic growth phase of the strains, analyzed relative to their optical densities at 600 nm, and normalized with FI of *Mtb* (pOLYG P_smyc_ *eGFP*) equal to 1. Data are representative of at least three biological replicates; the error bars indicate standard deviation; for statistical analysis groups were compared by Student’s unpaired, two-tailed *t-*tests, *p*-values: ^****^p < 0.0001, ^∗∗^*p* < 0.01, ns: not significant.

Conformational changes of the untranslated region (UTR) of the mRNA can be induced by a variety of small molecules, e.g., metabolites or drugs, and ion concentrations, leading to changes in expression levels of the downstream gene (Sinumvayo et al., 2018). These unique responsive oligonucleotides are termed riboswitches and represent a promising tool for reporter strain construction. A number of riboswitches have been identified in *Mtb* bacteria by sequence homology, including two homologous motifs upstream of the *Mtb* genes *rv1535* and *rv1806*. Based on transcriptomic profiling under ion starvation, these riboswitches were classified as Mg^2+^-responsive elements (Walters et al., 2006). We cloned both riboswitches with their native promoters upstream of the gene encoding mWasabi (Figure 5A) and integrated the new reporter constructs into the Giles attachment site of the *Mtb* chromosome. The new reporter strains, *Mtb* (Giles *attB*:P_np__1535_ *mWasabi*) and *Mtb* (Giles *attB*:P_np__1806_ *mWasabi*), showed Mg^2+^-dependent fluorescence responses *in vitro* inversely correlated to the concentration of the divalent ion. Grown under normal conditions with high levels of Mg^2+^ these strains were much less fluorescent than the episomally encoded pOLYG *eGFP* control (Figure 5B). While the FI of *Mtb* (Giles *attB*:P_np__1535_ *mWasabi*) was two times greater at the lower (10 μM) compared to the higher Mg^2+^ concentration (500 μM), *Mtb* (Giles *attB*:P_np__1806_ *mWasabi*) displayed fluorescence differences of sevenfold under these conditions (Figure 5C). The Mg^2+^ response was time dependent reaching maximal levels after approximately 4–7 days of incubation (Figure 5D).

### Increasing the FI of the Riboswitch-Based *Mtb* Reporter Strain

**FIGURE 6:**
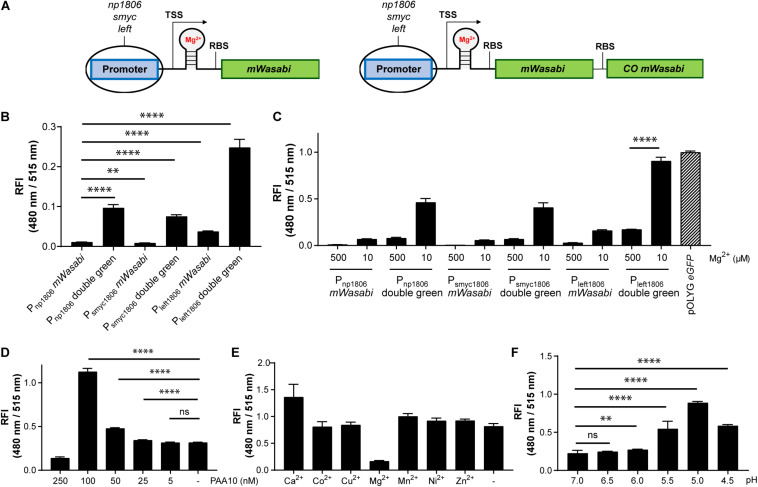
Using protein-riboswitch fusions and multicistronic constructs yielded increased fluorescence intensity while maintaining a Mg^2+^-specific response. **(A)** Design of Mg^2+^-responsive gene constructs. TSS indicates transcriptional start site; RBS indicates ribosome binding site. Influence of protein-riboswitch fusions and multicistronic constructs on **(B)** the relative fluorescence intensity (RFI) at a Mg^2+^ concentration of 500 μM and pH 7.0, and **(C)** Mg^2+^-dependent fluorescence increase at pH 7.0 after 7 days of incubation. RFIs of *Mtb* (Giles *attB*:P_left__1806_ double green) were detected **(D)** at a Mg^2+^ concentration of 500 μM and pH 7.0 when exposed for 4 days to different concentrations of the CorA inhibitor pyrimidinetrione amide analog 10 (PAA10), **(E)** RFI after 6 days coincubation at a Mg^2+^ concentration of 10 μM and pH 7.0 with the addition of calcium chloride (CaCl_2_; 1 mM), cobalt(II) sulfate (CoSO_4_; 100 μM), copper(II) sulfate (CuSO_4_; 40 μM), magnesium sulfate (MgSO_4_; 1 mM), manganese(II) sulfate (MnSO_4_; 150 μM), nickel(II) sulfate (NiSO_4_; 300 μM), or zinc sulfate (ZnSO_4_; 1 mM), **(F)** RFI after 6 days incubation in media with Mg^2+^ concentrations of 500 μM and different pH values. FIs were analyzed relative to the optical densities at 600 nm and normalized with FI of *Mtb* (pOLYG P_smyc_ *eGFP*) equal to 1. Data are representative of at least four biological replicates; the error bars indicate standard deviation; for statistical analysis groups were compared by Student’s unpaired, two-tailed *t*-tests, *p*-values: ^****^p < 0.0001, ^∗∗^*p* < 0.01, ns: not significant.

While the data above confirmed the utility of the *np1535* and *np1806* riboswitch elements in generating Mg^2+^-responsive reporter systems, the overall FIs of the chromosomally-integrated constructs were 40- and 86-fold, respectively, lower than the corresponding signal achieved by the constitutively active reference strain *Mtb* (pOLYG P_smyc_ *eGFP*) (Figure 5B). In order to improve the readout signal of the Mg^2+^ responsive reporter, we applied the strategies described above, including (a) promoter exchange and (b) formation of multicistronic FP constructs. The essential DNA element representing the responsive riboswitch was identified based on published transcriptional start site (TSS) data (Cortes et al., 2013) and by sequence alignment of the DNA regions upstream of the *Mtb* genes *rv1535* and *rv1806*. An oligonucleotide region of around 250 base pairs was determined, which showed 70% sequence homology (Supplementary Table 5). The predicted RBS, AGGAGG, and AGGAGA, of those genes closely resembled the consensus RBS of *Mtb* (DeJesus et al., 2013; Newton-Foot and Gey van Pittius, 2013), which suggests that they were suitable elements for strong gene expression systems without further modifications. Based on this analysis, we fused the putative core riboswitch of *np1806*, along with the native RBS, to either the *smyc* or *left* promoter. Furthermore, these new promoter-riboswitch constructs were placed upstream of either *mWasabi* or double green (Figure 6A). To determine the basal fluorescence of the *Mtb* strains carrying the newly designed plasmids, we measured signal intensity at a high Mg^2+^ concentration of 500 μM. While P_s__myc__1806_ and the native promoter led to similar FIs, *Mtb* (Giles *attB*:P_left__1806_ *mWasabi*) showed a fivefold increased brightness, which correlates with the fold-change we observed previously comparing the promoter elements P_s__myc_ and P_left^*^_ (Figures 2B, 6B and Supplementary Figure 9). When testing the FIs of the *Mtb* strains containing two gene copies of mWasabi, the results displayed a fluorescent signal output between seven and nine times higher than the reporter strains harboring a single *mWasabi* gene (Figure 6B and Supplementary Figure 10). Importantly, the Mg^2+^ responsiveness of the new *Mtb* reporter strains remained intact despite manipulations of the promoter and gene regions. *Mtb* (Giles *attB*:P_left__1806_ double green) showed a fivefold fluorescence enhancement at low Mg^2+^ concentration (10 μM) compared to high cation levels (500 μM), achieving a similar brightness to *Mtb* (pOLYG P_smyc_ *eGFP*) (Figure 6C).

### The *Mtb* (Giles *attB*:P_left__1806_ Double Green) Reporter Strain Selectively Responds to Mg^2+^

The fluorescence response of the novel riboswitch-based reporter strain was further analyzed across a broader range of physiologically relevant Mg^2+^ conditions and demonstrated a robust concentration and time dependency (Supplementary Figure 10). To verify Mg^2+^ specificity the influence of a known inhibitor (PAA10) of the mycobacterial Mg^2+^ transporter CorA (Park et al., 2019) was examined. A significant fluorescence increase was detected from an inhibitor concentration of 25 nM, increasing to a maximum at inhibitor concentrations close to its MIC (250 nM) (Supplementary Figure 2A). At a concentration of 100 nM, the detected FI of the reporter strain suggests that intracellular Mg^2+^ levels are lower than those obtained at external media MgSO_4_ concentrations of 10 μM (Figure 6D and Supplementary Figure 2B). To exclude promiscuity towards other divalent ions, the response profile of the novel reporter strain *Mtb* (Giles *attB*:P_left__1806_ double green) was explored under low magnesium (10 μM) and high divalent ion (Ca^2+^, Co^2+^, Cu^2+^, Mn^2+^, Ni^2+^, Zn^2+^) concentrations, close to their MIC values or at 1 mM if the MIC was greater than 2.5 mM (Supplementary Figure 11). None of the tested ions had a significant impact on the FI induced by the absence of Mg^2+^ ions, indicating high specificity of the riboswitch (Figure 6E and Supplementary Figure 11).

### Influence of Environmental pH on the Fluorescence Response of *Mtb* (Giles *attB*:P_left__1806_ Double Green)

Based on previous data showing that lower pH values influence the Mg^2+^ levels required for mycobacterial growth (Buchmeier et al., 2000; Piddington et al., 2000), we predicted that acidic pH would impact the fluorescence signal of *Mtb* (Giles *attB*:P_left__1806_ double green). Screening the reporter’s response at high Mg^2+^ concentrations (500 μM) and a range of pH values supported this assumption. At pH 5.5, there was a 2.5-fold increase in the fluorescence signal compared to pH 7.0. When the pH dropped to 5.0, a fourfold increase in FI was observed and a threefold increase was measured for pH 4.5. These data imply that the intrabacterial Mg^2+^ levels are 50 times lower than environmental ion concentrations at pH 5.0 (Figure 6F and Supplementary Figure 12), suggesting that the Mg^2+^ uptake of *Mtb* is dependent on extracellular pH.

These results show that to fully understand the micromilieu, both pH and Mg^2+^ must be evaluated in parallel. Therefore, a reporter with the ability to simultaneously respond to pH and Mg^2+^ had to be constructed. In addition, this strain had to contain an expression system under the control of a constitutive promoter to exclude false positive or negative results based on fluctuations in protein expression levels.

### Expanding the FP Panel for *Mtb* Bacteria

For construction of a triple reporter, we took advantage of the variety of available FPs, in order to track multiple conditions simultaneously without spectral crosstalk. Eleven different genes (*mTagBFP2*, *mT-Sapphire*, *mTurquoise2*, *mTFP1*, *LSSmOrange*, *CyOFP1*, *Ypet*, *mPapaya*, *mOrange2*, *mKate2*, *mCardinal*) encoding FPs with a large variety of excitation and emission wavelengths were cloned downstream of the P_smyc_-*His* element within pL5 P_smyc_-*His*. Signal intensities of the new fluorescent *Mtb* strains were screened under different wavelength pairings and possible combinations with minimal spectral overlap were identified. In addition to *Mtb* (L5 *attB*:P_smyc_ *mWasabi*) and *Mtb* (L5 *attB*:P_smyc_ *mScarlet*), four other *Mtb* strains (L5 *attB*:P_smyc_ *mTagBFP2*, *LSSmOrange*, *mPapaya*, *mCardinal*) exhibited fluorescence characteristics which allowed signal detection in parallel (Supplementary Figures 13, 14).

### Generation of a Three-Color Fluorescent Strain to Simultaneously Monitor pH, Mg^2+^, and Protein Expression

**FIGURE 7:**
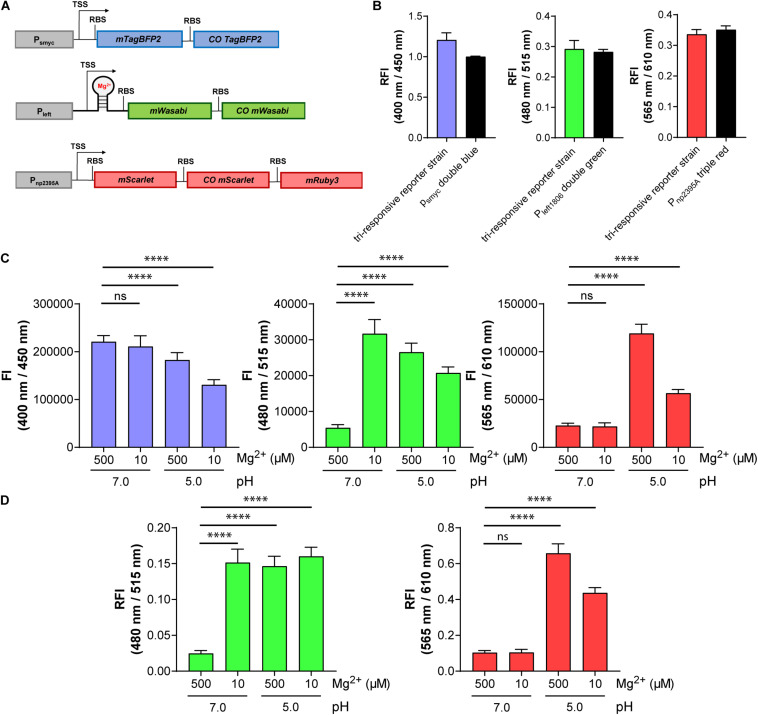
The triple reporter strain enables simultaneous visualization of three conditions in parallel using fluorescence signals at three different wavelengths: protein expression (blue), Mg^2+^ concentration (green), and pH (red). **(A)** Design of the gene constructs included in the tri-responsive reporter strain. TSS indicates transcriptional start site; RBS indicates ribosome binding site. **(B)** RFI of the triple reporter strain in comparison with the single reporter strains *Mtb* (L5 *attB*:P_smyc_ double blue), *Mtb* (Giles *attB*:P_left__1806_ double green), and *Mtb* (Giles *attB*:P_np__2395__A_ triple red) detected at their respective optimal excitation and emission wavelengths in media with pH 7.0 and a Mg^2+^ concentration of 500 μM. Data were normalized with FIs of *Mtb* (L5 *attB*:P_smyc_ double blue), *Mtb* (pOLYG P_smyc_ *eGFP*), and *Mtb* (pOLYG P_smyc_ *mCherry*) equal to 1. **(C)** FIs of the three fluorescent proteins detected after 6 days of *Mtb* (tri-responsive reporter) growth in neutral (pH 7.0) and acidic pH (pH 5.0), as well as high (500 μM) and low (10 μM) Mg^2+^ concentrations. **(D)** FIs at 480/515 nm (green) and 565/610 nm (red) depicted relative to changes in protein expression levels (400/450 nm, blue). All FIs were detected during the logarithmic growth phase of the strain and analyzed relative to its optical densities at 600 nm. Data are representative of at least three biological replicates; the error bars indicate standard deviation; for statistical analysis groups were compared by Student’s unpaired, two-tailed *t*-tests, *p*-values: ^****^p < 0.0001, ns: not significant.

We exploited the compatibility of mWasabi, mScarlet, and mTagBFP2 to generate the intended triple reporter strain detecting pH, Mg^2+^ concentration, and protein expression levels. The newly identified reporter constructs P_np__2395__A_-triple red and P_left__1806_-double green were introduced into different multiple cloning sites within the same plasmid, which integrates into the Giles *attB* site of the mycobacterial chromosome. In addition, the constitutive *smyc* promoter was cloned upstream of *mTagBFP2* and *COmTagBFP2* and introduced into a separate plasmid integrating into the L5 *attB* site (Figure 7A). The FIs of the new triple reporter *Mtb* strain (protein expression: blue, λ_ex_:400 nm/λ_em_:450 nm; Mg^2+^ concentration: green, λ_ex_:480 nm/λ_em_:515 nm; pH: red, λ_ex_:565 nm/λ_em_:610 nm), carrying both plasmids integrated at different sites of the chromosome, were compared to those harboring the individual reporter constructs separately. No significant differences in signal intensities were detected (Figure 7B). Furthermore, the new tri-responsive reporter strain had identical growth characteristics in 7H9 medium compared to the strains containing the single multicistronic gene constructs under control of the *smyc* promoter or a WT strain, respectively (Supplementary Figure 15).

### Testing Response Characteristics of the Tri-Responsive Reporter Strain

The triple reporter strain was cultured under a range of physiologically relevant pH values and Mg^2+^ concentrations and the OD_600_-normalized FIs were measured. Red fluorescence increased significantly when the bacteria were cultured in media at pH 5.0, versus pH 7.0, as observed previously with the individual pH-responsive reporter strain, and the FI at the low pH was twofold greater for high (500 μM) compared to low (10 μM) Mg^2+^ concentration (Figure 7C, right panel). Green FI increased to a similar extent when the strain was grown under either low Mg^2+^ concentrations (10 μM), acidic pH (5.0), or a combination of the two conditions (Figure 7C, middle panel). A sixfold difference in green FI was measured when comparing 10 to 500 μM Mg^2+^ levels at pH 7.0. The green fluorescence signal output was higher in low Mg^2+^ and neutral pH compared to low or high Mg^2+^ and acidic pH. A decrease in blue FI was observed under acidic conditions, with a stronger reduction in media containing 10 μM Mg^2+^ (Figure 7C, left panel). Plotting the red and green FIs relative to the blue signal (in effect normalizing for protein expression levels; Figure 7D) led to alterations in the data, particularly evident in the reduced variation between the Mg^2+^ (green) responses across all of the tested “activating” conditions. In all cases, the response of the triple reporter strain was time dependent (Supplementary Figures 16–19).

## Discussion

Using a combination of recombinant DNA and protein optimization techniques, we have developed a novel reporter strain which, despite chromosomal integration, exhibits high FIs. This triple reporter strain is able to monitor protein expression, pH, and, for the first time, Mg^2+^ concentration simultaneously. One method to accomplish the brightness of the new strain was to exploit the variety of available FPs. Although several green and red FPs are described to have higher molecular brightness than eGFP and mCherry, those two FPs are still most frequently used for the development of *Mtb* reporter strains (Abramovitch, 2018; MacGilvary and Tan, 2018). In this study, our green FP of choice was mWasabi, an FP derived from mTFP1 that exhibits high photostability, preferable narrow excitation and emission spectra, and improved molecular brightness relative to eGFP (Ai et al., 2008). While mWasabi has previously been employed in various mycobacterial model systems (Takaki et al., 2013; Ganji et al., 2015; Bernut et al., 2016; Sukheja et al., 2017), to the best of our knowledge it has never been quantitatively compared to eGFP as a reporter protein in this genus. Here, we show that *Mtb* bacteria expressing mWasabi had a promising threefold higher FI compared to *Mtb* expressing eGFP from the same promoter, supporting its use as an improved mycobacterial reporter gene. One potential disadvantage of mWasabi is its sensitivity towards acidic pH, similar to that of eGFP (Zhou et al., 2012; Tanida et al., 2014). However, for our purpose the pH dependence was not of great concern, since *Mtb* is known to maintain its intra-bacterial pH between 6.8 and 7.5, even when exposed to acid environmental conditions (Vandal et al., 2008). Besides mWasabi, ZsGreen, a GFP-like protein isolated from a *Zoanthus* species (Matz et al., 1999), led to a comparable high fluorescence signal when expressed in *Mtb* bacteria. The protein expression level of ZsGreen was 3.5-fold lower compared to mWasabi, which could be an advantage regarding energy cost and possible toxicity associated with excessive protein production. Despite these benefits, we decided to proceed with mWasabi, since this green FP had already been successfully used for *in vivo* imaging of *Mycobacterium marinum* (Stirling et al., 2020). Future studies will explore the utility of mNeonGreen, the brightest monomeric green FP yet described (Shaner et al., 2013). While molecular brightness, calculated as the product of extinction coefficient and quantum yield, might be used as a first indication for the applicability of FPs in reporter strain development, there was no correlation between molecular brightness and final signal intensity of fluorescent *Mtb* bacteria, in this study (Supplementary Figure 20). The discrepancy between molecular brightness, protein expression level, and FI, might be explained by proper intra-bacterial folding of FPs (Kremers et al., 2007).

While a variety of red FPs have been previously screened as alternatives to mCherry, so far only the tandem dimer tdTomato was identified to exceed the brightness of mCherry when expressed in mycobacteria (Carroll et al., 2010; Kong et al., 2016). Recently, mScarlet was engineered with the highest ever calculated molecular brightness and the longest fluorescence lifetime in the red FP spectral class. Furthermore, it shows high photostability (t_1__/__2_ = 277 s) and acid tolerance (Bindels et al., 2017). Based on these data, we introduced mScarlet into *Mtb* and observed a higher FI for *Mtb* (L5 *attB*:P_smyc_ *mScarlet*) compared to *Mtb* (L5 *attB*:P_smyc_ *mCherry*). The high readout signal in combination with the low expression level of mScarlet is promising for reporter strain development. In addition, its monomeric character is favorable over the tandem dimer of tdTomato for the construction of multicistronic FP systems.

In order to monitor three conditions in parallel, we had to introduce an additional FP with excitation and emission wavelengths varying from those of mWasabi and mScarlet. Four FPs, mTagBFP2 (Subach et al., 2011), LSSmOrange (Shcherbakova et al., 2012), mPapaya (Hoi et al., 2013), and mCardinal (Chu et al., 2014), were identified as potential candidates. Comparing the fluorescence output signals at the optimal wavelengths for each FP identified *Mtb* (L5 *attB*:P_smyc_ *mTagBFP2*) as the brightest strain with around 100-fold higher FI compared to *Mtb* (L5 *attB*:P_smyc_ *mCardinal*) (Supplementary Figure 21). Based on the low signal intensity detected for *Mtb* bacteria expressing mCardinal, we excluded this FP for reporter strain development. However, with excitation and emission wavelengths with maxima at 604 and 659 nm, it might still be a promising candidate for future *in vivo* studies. We also did not choose mPapaya, since spectral cross talk with mScarlet and mWasabi are more likely to occur with this yellow FP compared to LSSmOrange and mTagBFP2. Both LSSmOrange (data not shown) and mTagBFP2 were successfully used to construct analogous triple reporter strains. In both cases, expressing all three FPs in a single strain did not impact the FIs and response characteristics of each reporter system. However, microscopes are commonly equipped with filter sets suitable for detection of this blue FP, but not LSSmOrange, which has excitation and emission wavelengths at 437 and 572 nm. Furthermore, photoconvertible characteristics were described for this long stokes shift (LSS) FP. Irradiation with a strong 400 nm laser resulted in a change in the absorption peak of LSSmOrange from 437 to 553 nm and thereby in the loss of the LSS (Fron et al., 2015). While the laser intensity of confocal microscopes is most likely not strong enough to induce this photoconversion, we decided not to continue with LSSmOrange. Additional benefits of mTagBFP2 are its high photostability (Subach et al., 2011), as well as a low sensitivity to pH and hypoxia (Tu et al., 2014), two environmental conditions *Mtb* bacteria are often exposed to within the lungs.

To increase brightness all of the reporter constructs contained at least two genes encoding FPs with similar excitation and emission wavelengths. Insertion of a second gene had the intended additive effect resulting in a twofold fluorescence increase. Construction of a three gene system led only to a slight signal enhancement compared to the two gene element. These results are in line with studies from Lim et al. (2011), showing that protein expression levels are anti-proportional to the distance of the gene location to the TSS.

Introducing the *left* promoter with a modified RBS was another successful method to obtain increased FIs of mycobacterial strains. P_left_ was first identified as a promoter in the genome of the mycobacteriophage L5 (Nesbit et al., 1995). An initial phage-based study using a P_left_-*mVenus* expression system suggested 100-fold greater FI compared to the analogous *hsp60* construct (Jain et al., 2012). However, following reports introducing P_left_ directly into *Mtb* via replicating plasmids detected only a threefold increase, leading to FIs similar to those of P_s__myc_-gene constructs (Ehrt et al., 2005; Kanno et al., 2016; Kong et al., 2016). In our hands, *Mtb* bacteria containing P_left_-*eGFP* showed comparable FIs to *Mtb* (L5 *attB*:P_h__sp__60_ *eGFP*). The only difference between our P_left_ and the one used by Jain et al. (2012) and Kanno et al. (2016) were three nucleotides in front of the start codon (cat → tcc), which were exchanged in order to construct an *Nco*I cloning site. The apparent discrepancies in expression levels may be due to effects on RNA folding and thereby protein production (Horbal et al., 2018). Expression from the *left* promoter was further increased by replacement of its RBS with the one from P_smyc_ creating P_left^*^_, which allowed us to generate integrated fluorescent strains with FIs exceeding those obtained using episomal expression from pOLYG by several fold.

In order to exploit the strength of P_left_ for reporter strain development, the responsive element had to be outside of the promoter region. In this study, we utilized riboswitches. Their high ligand affinity and specificity, as well as a precise dose-dependence, makes them ideal candidates for reporter strain development (Sherwood and Henkin, 2016; McCown et al., 2017; Sinumvayo et al., 2018). While a number of riboswitches, including cyclic-di-AMP-, cobalamin-, tRNA-, and Mg^2+^-sensing RNA elements, have been predicted in *Mtb* using sequence homology (Arnvig and Young, 2012; Nawrocki et al., 2015; Schwenk and Arnvig, 2018), only a single B_12_-dependent riboswitch upstream of the *metE* gene has been experimentally validated to date (Warner et al., 2007). The first synthetic promoter-riboswitch fusion was introduced in 2012 into mycobacteria by using a theophylline-responsive riboswitch, which enabled the construction of a new conditional gene knockdown system (Seeliger et al., 2012). To date, however, riboswitches have not been exploited to design novel reporter strains in mycobacteria.

In this study, we applied two Mg^2+^ responsive elements, also termed ykok leader or Mbox, for this purpose, which were described to be located upstream of the genes *rv1535* and *rv1806*. Transcription of both of these genes was known to be strongly upregulated 13.2- and 5.7-fold, respectively, in *Mtb* bacteria when Mg^2+^-starved for 48 h (Walters et al., 2006). In line with this, we detected a fluorescence increase for both *Mtb* strains carrying the native promoter elements upstream of *mWasabi* when MgSO_4_ concentration in culture was reduced from 500 to 10 μM. The signal enhancement was 3.5 times higher for *Mtb* (Giles *attB*:P_np__1806_ *mWasabi*) compared to *Mtb* (Giles *attB*:P_np__1535_ *mWasabi*).

Construction of a P_left_-riboswitch(1806) fusion led to a fivefold signal increase compared to the native promoter element, while keeping Mg^2+^-response characteristics intact. The new *Mtb* (Giles *attB*:P_left__1806_ double green) reporter strain exclusively responded to Mg^2+^ but not to other divalent ions and thus will enable specific monitoring of Mg^2+^ concentrations *in vivo*, even in the presence of Cu^2+^, and Zn^2+^ ions, which were previously described to reach high μM concentrations in *Mtb*-containing phagosomes (Wagner et al., 2005).

While other divalent ions did not interfere with the reporter response, pH values of 5.5 or lower led to an increase of the fluorescence signal compared to neutral pH, even in media with high Mg^2+^ concentrations (500 μM). The pH-dependence of *rv1806* expression has already been described (Rohde et al., 2007). The mechanism of this pH response, however, is still poorly understood. Destruction of a DNA region, controversially expressing either a potential nucleic acid binding protein AprA (*rv2395A*) or the small non-coding RNA (ncRNA) *mcr7* (Abramovitch et al., 2011; Solans et al., 2014), led to reduced expression of *rv1806* (Abramovitch et al., 2011). While *aprA*/*mcr7* is under control of the pH-dependent PhoPR two component system, *rv1806* is expressed at WT levels in a PhoP transposon mutant (Abramovitch et al., 2011). More confusingly, the transcriptomic profiling strongly differs between PhoP mutants and Mg^2+^-starved WT bacteria. However, disruption of PhoPR prevents growth in low-Mg^2+^ media and addition of excess Mg^2+^ during infection can partially overcome the growth defect of PhoP mutants in THP-1 macrophages (Walters et al., 2006). Interestingly, in our study, *Mtb* (Giles *attB*:P_np__2395__A_ triple red) indeed showed a reduced output signal when Mg^2+^ levels were lowered at acidic pH, and this could not be fully explained by reduction of total protein expression levels. Walters et al. (2006) predicted the pH and Mg^2+^ correlative effects to be based on cell wall remodeling. However, this has still to be confirmed. Nevertheless, it underlines the necessity to introduce a pH-responsive gene system into the Mg^2+^-dependent *Mtb* reporter strain in order to enable differentiation between fluorescence increase based on acid environmental conditions or low Mg^2+^ concentrations.

Our novel triple reporter strain, detecting Mg^2+^ levels, pH and protein expression levels, will be used in the future to study the importance of Mg^2+^ ions *in vivo* and the potential of Mg^2+^ transporters as drug targets. One potential candidate is the mycobacterial protein of the proline-glutamate family PE20, whose expression is controlled by the riboswitch(1806), used in this study. PE20 in combination with the proline-proline-glutamate containing protein 31 (PPE31) was shown to be responsible for Mg^2+^ uptake across the outer membrane of *Mtb* in a phthiocerol dimycocerosate (PDIM)-dependent manner (Wang et al., 2020).

The transporter, predicted to be responsible for Mg^2+^ uptake across the inner membrane of *Mtb*, is the transmembrane protein CorA. Recently, two groups have identified novel anti-mycobacterial molecules, whose efficacy was Mg^2+^-dependent. Mutations of resistant strains exclusively mapped to the gene *rv1239c*, which encodes CorA (Lopez Quezada et al., 2019; Park et al., 2019). We explored one of the compounds from these studies, PAA10 (Park et al., 2019). Treatment of the triple reporter strain with this compound showed an increased fluorescence signal at concentrations close to its known MIC value. When the reporter was cultured in high (500 μM) Mg^2+^ levels, the FI, detected at a PAA10 concentration of 100 nM, exceeded that measured at 10 μM environmental MgSO_4_, confirming the proposed mechanism of action of intracellular Mg^2+^ starvation. Our new reporter strain will be used to identify Mg^2+^ concentrations of different *Mtb* lesions within the lungs which may address whether the lack of *in vivo* efficacy of PAA10 (Park et al., 2019) was due to poor exposure to the compound at the site of infection or due to high concentrations of Mg^2+^ in the *Mtb* microenvironment.

Future applications of the reporter strains described in this study will invariably require more physiologically relevant model systems than homogenous bacterial cultures, and studies to gauge their utility in cell culture macrophage and animal infection models are ongoing. It is unknown, for example, how reporter gene expression will be affected by less discrete, and temporally fluctuating, changes in pH or ion concentrations as expected to be encountered in more complex biological systems. Similarly, while we could isolate live fluorescent bacteria from mice 13 weeks post infection, it is not clear what effect transgene overexpression may have on long-term fitness and pathogenicity of the organism. Furthermore, while the analytical methods used here (fluorescence plate reader) emphasized population averages, future work will utilize confocal microscopy to analyze individual bacterial responses within spatially and temporally defined regions. Inter-bacterial heterogeneity at the single cell level will provide important information about the heterogeneity of the local milieu. While mindful of these caveats that arise from migrating technical assays from *in vitro* to *in vivo*, we are encouraged by prior work in the application of engineered mycobacterial reporter strains to study *in vivo* phenomena (Tan et al., 2013; Sukumar et al., 2014).

Besides the Mg^2+^-dependent riboswitch used in this study, other responsive RNA elements might be attractive tools for reporter strain development in the future. Only a few riboswitches have been identified in mycobacteria to date. However, it is likely that the mycobacterial genome contains additional ones, which still need to be discovered (McCown et al., 2017). Furthermore, it should be possible to exploit riboswitches of other species, since the response mechanism is solely RNA-based and independent of species-specific regulatory proteins. In addition, riboswitches allow for the possibility of *de novo* design to any ligand. Indeed, the first *de novo* designed riboswitch was recently developed to the second-line anti-TB drug ciprofloxacin (Groher et al., 2018). Using antibiotic-dependent riboswitches for *Mtb* reporter strain development will allow the direct monitoring of drug concentration across diverse lesion types *in vivo* and offers a promising approach to get deeper insights into treatment efficiency.

## Data Availability Statement

The original contributions presented in the study are included in the article/Supplementary Material, further inquiries can be directed to the corresponding author/s.

## Ethics Statement

The animal study was reviewed and approved by NIAID Animal Care and Use Committee.

## Author Contributions

KK, GP, HB, and CB designed the experiments. KK, AB, GP, MA, H-JY, HF, and SG conducted the experiments. KK, AB, GP, MA, H-JY, KM-B, HB, and CB wrote the manuscript. All authors contributed to the article and approved the submitted version.

## Conflict of Interest

The authors declare that the research was conducted in the absence of any commercial or financial relationships that could be construed as a potential conflict of interest.

## Footnotes

1 http://www.jcat.de/Start.jsp
