# Supplementary material for: Development and Optimization of Chromosomally-Integrated Fluorescent Mycobacterium tuberculosis Reporter Constructs
Source: Front Microbiol. 2020 Dec 9;11:591866. doi: 10.3389/fmicb.2020.591866 (PMC7755994; doi:10.3389/fmicb.2020.591866)
Supplement: Supplementary file 1 [file Data_Sheet_1.PDF]

Supplementary Material

1 Supplementary methods

1.1 Restriction enzyme-based cloning

<u>Construction of new plasmids</u>
Polymerase chain reaction (PCR; 50 µl total volume) using Q5 high-fidelity DNA polymerase (NEB) with an extension temperature and rate of 72 ºC and 1 kbp/30 s was performed to amplify the DNA regions of interest.
To confirm the size of the amplicon, 5 µl of each PCR product was analyzed by agarose gel electrophoresis (125 V, 30 min).
To purify the PCR products, DNA binding buffer (Zymo) was added in 4 times excess and each mixture was transferred to a spin column (Zymo). After centrifuging (21130×g, RT, 30 s), the columns were washed twice with DNA wash buffer (Qiagen), and the PCR products were eluted using nuclease-free water (50 µl). The concentration of DNA was detected using a Nanodrop (Thermo Fisher Scientific).

The amplified DNA regions (inserts) and the respective plasmids were digested using FastDigest restriction enzymes (Thermo Fisher Scientific). The reactions were set up in 20 µl volumes with the PCR product or plasmid (100-150 ng/µl, 16 µl), 10X FastDigest Buffer (Thermo Fisher Scientific; 2 µl), and restriction enzymes (1 µl each). If cloning into a single restriction site, FastAP Thermosensitive Alkaline Phosphatase (Thermo Fisher Scientific, 1 U/µL; 1 µl) was added to the plasmid digest. After incubating (37 °C, 1 h), the entire reactions were run on a 1 % agarose gel. The gel was viewed under UV light and the desired bands were excised. QG buffer (Qiagen; 500 µl) was added to each tube containing a gel fragment and the samples were incubated at 45 °C until complete dissolution was achieved. Each mixture was then transferred to a spin column (Zymo), centrifuged (21130×g, RT, 1 min), washed two times with DNA wash buffer (Qiagen), and DNA eluted with nuclease-free water (10-15 µl). The concentration was detected using a Nanodrop.

Digested DNA fragments were ligated using either Instant Sticky-End Master Mix (NEB) or T4 DNA ligase (NEB). For reactions with Instant Sticky-End Master Mix, the digested plasmid (100 ng) was combined with the digested insert (50-100 ng), along with Instant Sticky-End Master Mix (5 µl) and nuclease-free water, to a final reaction volume of 10 µl. These reactions were available for transformation immediately. The same amounts of plasmid and insert DNA were used for reactions with T4 DNA ligase, along with 10X T4 DNA ligase buffer (2 µl), T4 DNA ligase (1 µl), and nuclease-free water, to a final reaction volume of 20 µl. To ensure complete ligation, reactions using T4 DNA ligase were incubated overnight at RT and were available for transformation the following day.

Transformation of *E. coli*

Escherichia coli (*E. coli*) NEBα were transformed with the ligations via heat shock. The ligation reactions (2-3 µl) were added to competent cells (50 µl) and incubated on ice (30 min). The tubes were then placed at 42 °C (30 s) and transferred back to ice (5 min). Super Optimal Broth with Catabolite repression (SOC; NEB) was added to each tube and the reactions were incubated (37°C, 1 h). Transformations were plated on LB agar plates containing the appropriate antibiotic and incubated overnight (37 °C).

Colony PCR

Transformants were screened using Colony PCR. Each PCR reaction was comprised of NEB OneTaq High GC PCR MasterMix (NEB, containing Taq polymerase with an extension temperature and rate of 68 ºC and 1 kbp/1 min; 5 µl), forward primer (0.5 µl), reverse primer (0.5 µl), nuclease-free water (5 µl), and a small bit of a colony picked with a pipette tip. The PCR products were examined on a 1 % agarose gel and positive clones were grown overnight in LB medium containing the corresponding antibiotic. DNA was isolated from cultures via the Zyppy Plasmid Miniprep Kit (Zymo).

1.2 Oligo annealing

A 6x-*His* tag was incorporated into the plasmid of interest via oligo annealing. Oligonucleotides (100 µM, 1 µl each) were added to separate tubes containing 10X Anza polynucleotide kinase (PNK) buffer (Thermo Fisher Scientific; 4 µl), nuclease-free water (33 µl), and Anza PNK (Thermo Fisher Scientific; 2 µl) and subjected to 20 °C (15 min), 80 °C (5 min), and a 12 °C hold. The primer reactions (20 µl each) were combined along with nuclease-free water (60 µl) and incubated (95 °C, 3 min). After incubation, the reactions were slowly cooled to RT. The desired plasmid was digested using FastDigest restriction enzymes and alkaline phosphatase, following the procedure described above. After incubation (37 °C, 1 h), the enzymes in the digest were heat inactivated (65 °C, 15 min). The digested plasmid was column purified as described above and eluted in nuclease-free water (15-20 µl). The digested, dephosphorylated plasmid and the annealed, phosphorylated oligos were ligated using 100-150 ng and 10 ng, respectively, in a 15 µl reaction.

1.3 Gibson Assembly

The DNA insert of interest was amplified with primers containing oligonucleotide regions homolog with the flanking DNA regions of the plasmid's cloning sites. The plasmid was either digested using restriction enzymes or PCR amplified. The amplicons and digested plasmids were gel purified. For ligation plasmid DNA (50-100 ng), insert DNA (50 ng), NEBuilder HiFi DNA assembly MasterMix (NEB; 10 µl), and nuclease-free water were combined in a tube to a final total volume of 20 µl. The sample was incubated (50 ºC, 15 min), chilled on ice, and subsequently applied for transformation, as described above.

1.4 Generation of chemically competent *E. coli*

E. coli NEBα was grown overnight in LB medium containing magnesium chloride ($MgCl_2$, 10 mM) (37 ºC). This culture was sub-inoculated (1:100) into LB (50 ml) containing $MgCl_2$ (10 mM) and further incubated (37 ºC). When the OD_{600} reached 0.6, the flask was chilled on ice (10 min) and centrifuged (3000×g, 4 ºC, 10 min). The culture was resuspended in pre-chilled TFB I (Table S2; 10 ml). After resuspension, additional TFB I (40 ml) was added and the sample was chilled on ice (2 h). After centrifugation (3000×g, 4 ºC, 10 min), the culture was resuspended in TFB II (Table S2; 4 ml). The suspension was aliquoted into sterile tubes (100 µl each), flash frozen, and stored (-80 ºC) for future use.

1.5 Generation of electrocompetent *Mtb*

A culture of *Mycobacterium tuberculosis* (*Mtb*) H37Rv and HN878, respectively, were grown in 7H9 ADGNTw (Table S2) medium (100 ml, 37 ºC) to an OD_{600} of 0.6. Glycine (2 M) was added to achieve a final concentration of 0.2 M. After overnight incubation, the culture was centrifuged (3000×g, 20 ºC, 20 min) and resuspended in 10 % glycerol. The bacteria were washed twice with 10 % glycerol (10 ml each), resuspended in 10 % glycerol (5 ml) and aliquoted into Eppendorf tubes (400 µl each), and stored (-80 ºC) for future use.

1.6 Genomic DNA extraction

A culture of *Mtb* H37Rv (OD_{600}: 0.5, 1 ml) was centrifuged (20293×g, RT, 2 min). The pellet was resuspended by adding 1x TE buffer (Thermo Fisher Scientific) and Chelex buffer (Table S2; 200 µl each), subsequently incubated (56 °C, 30 min, then 100°C, 15 min) and centrifuged (21130×g, RT, 3 min). The supernatant was transferred to a new vial and stored (-20 °C).

1.7 Growth curve

Mtb strains were grown to mid-log phase in 7H9 ADGNTw medium. The precultures were diluted in the same medium to an OD_{650} of 0.003 (10 ml) and incubated for 7 d at 37 °C. The OD_{650} was analyzed daily.

2 Supplementary Figures

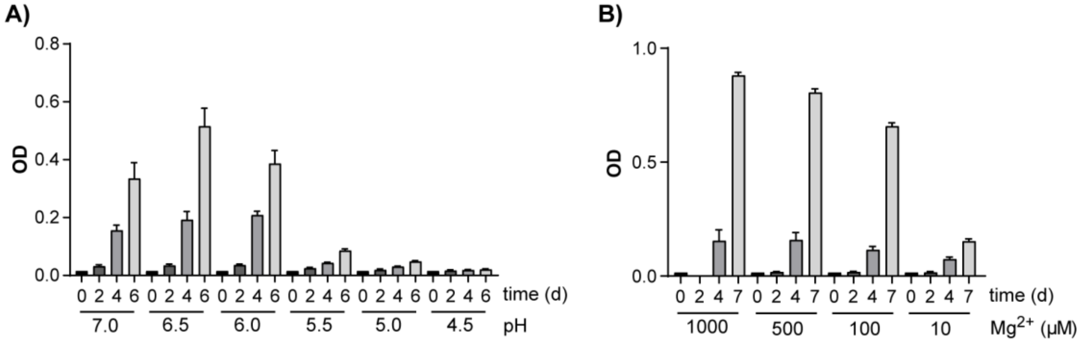

Figure S1. Mycobacterial growth is influenced by environmental pH and Mg^{2+} concentrations. **(A)** Growth characteristics of *Mtb* H37Rv cultured in Sauton's medium at a Mg^{2+} concentration of 500 µM and various pH values. **(B)** Growth characteristics of *Mtb* H37Rv determined in media with pH 7.0 and different Mg^{2+} concentrations. Optical densities (ODs) at 600 nm were measured over a week at different time points. Data are representative of at least four biological replicates; the error bars indicate standard deviation.

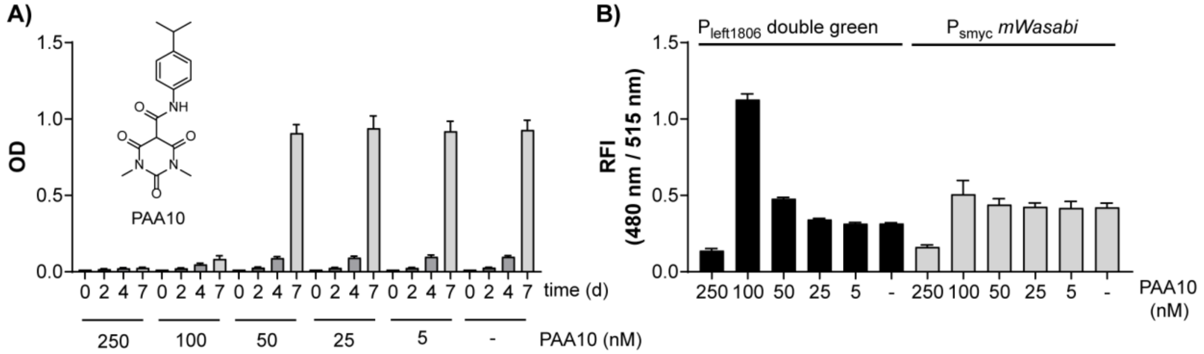

Figure S2. The CorA inhibitor PAA10 led to mycobacterial growth defects and increased fluorescence intensities of a Mg^{2+}-responsive reporter strain. (A) Growth characteristics of *Mtb* H37Rv at pH 7.0 and Mg^{2+} concentrations of 500 µM in presence of different concentrations of pyrimidinetrione amide analogue 10 (PAA10). Optical densities (ODs) at 600 nm were determined over a week at different time points. **(B)** Relative fluorescence intensities (RFIs) of the reporter strain *Mtb* (Giles *attB*::$P_{left1806}$ double green) and the control strain *Mtb* (Giles *attB*::P_{smyc} *mWasabi*) detected after 4 days incubation at pH 7.0 and a Mg^{2+} concentration of 500 µM in presence of different concentrations of PAA10. FIs were analyzed relative to their optical densities at 600 nm and normalized with FI of *Mtb* (pOLYG P_{smyc} *eGFP*) equal to 1. Data are representative of eight biological replicates; the error bars indicate standard deviation.

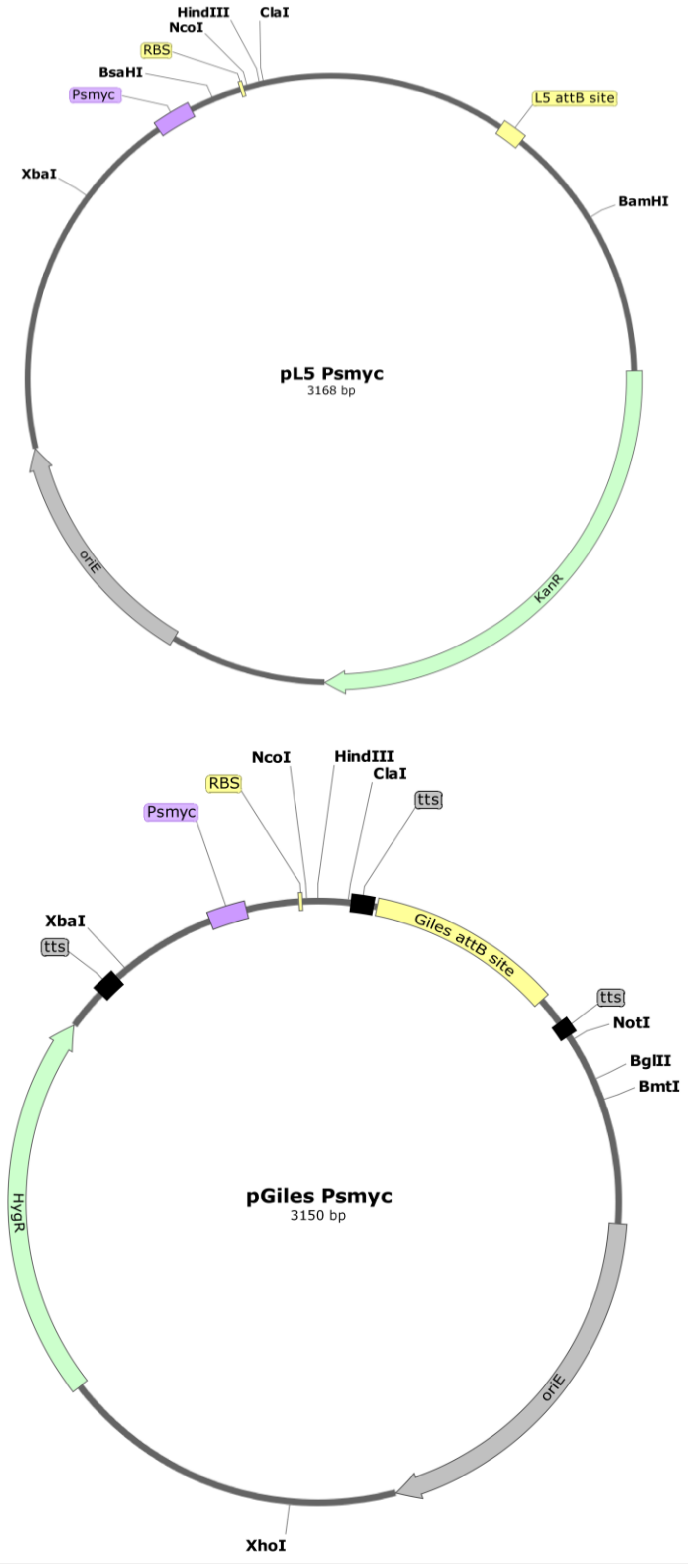

Figure S3. Integrative plasmids used in this study. Only restriction sites used in this study are shown, ribosome binding site (*RBS*), attachment site (*attB*), origin of replication of *E. coli* (*oriE*), transcriptional terminators (*tts*), resistance marker (Kanamycin (Kan^r), Hygromycin (Hyg^r)).

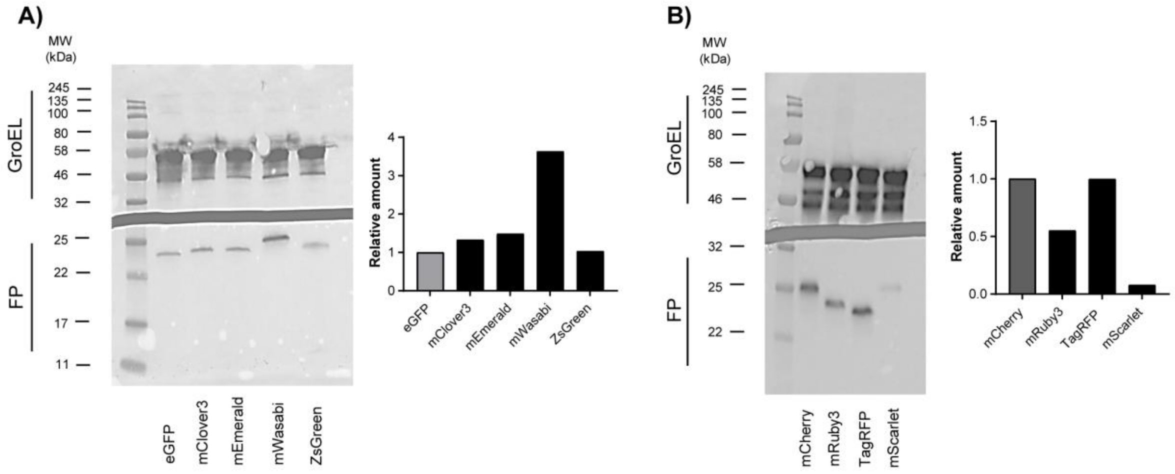

Figure S4. Mycobacterial expression levels of various green and red fluorescent proteins. Expression levels of different **(A)** green and **(B)** red fluorescent proteins (FPs), expressed in *Mtb* H37Rv bacteria under the control of the *smyc* promoter, and analyzed via western blot. As loading control, the protein concentration of the chaperon GroEL was determined. The amounts of the FPs were quantified and normalized with expression levels of *Mtb* (L5 *attB*::P_{smyc} *eGFP*) or *Mtb* (L5 *attB*::P_{smyc} *mCherry*) equal to 1. One out of two independent experiments shown.

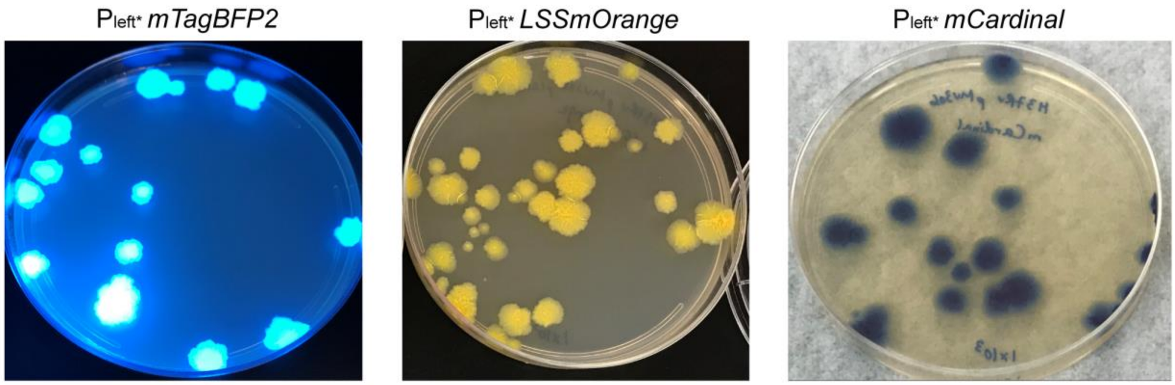

Figure S5. Colonies of *Mtb* strains expressing mTagBFP2, LSSmOrange, or mCardinal under the control of the *left promoter are visibly colored.** Fluorescent *Mtb* mutants grown on agar plates and imaged either under UV-light (*Mtb* (L5 *attB*::P_{left*} *mTagBFP2*)) or in visible light (*Mtb* (L5 *attB*::P_{left*} *LSSmOrange*), *Mtb* (L5 *attB*::P_{left*} *mCardinal*)).

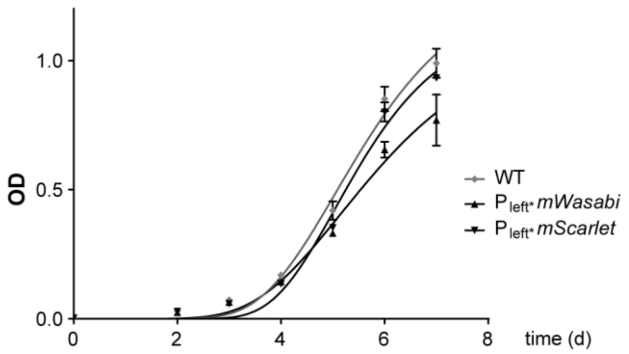

Figure S6. P_{left*} strains had similar growth characteristics compared to a WT strain. Growth rates of *Mtb* (L5 *attB*::P_{left*} *mWasabi*), *Mtb* (L5 *attB*::P_{left*} *mScarlet*), and an *Mtb* H37Rv WT strain in 7H9 medium were detected by OD_{650}. Data are representative of three biological replicates; the error bars indicate standard deviation.

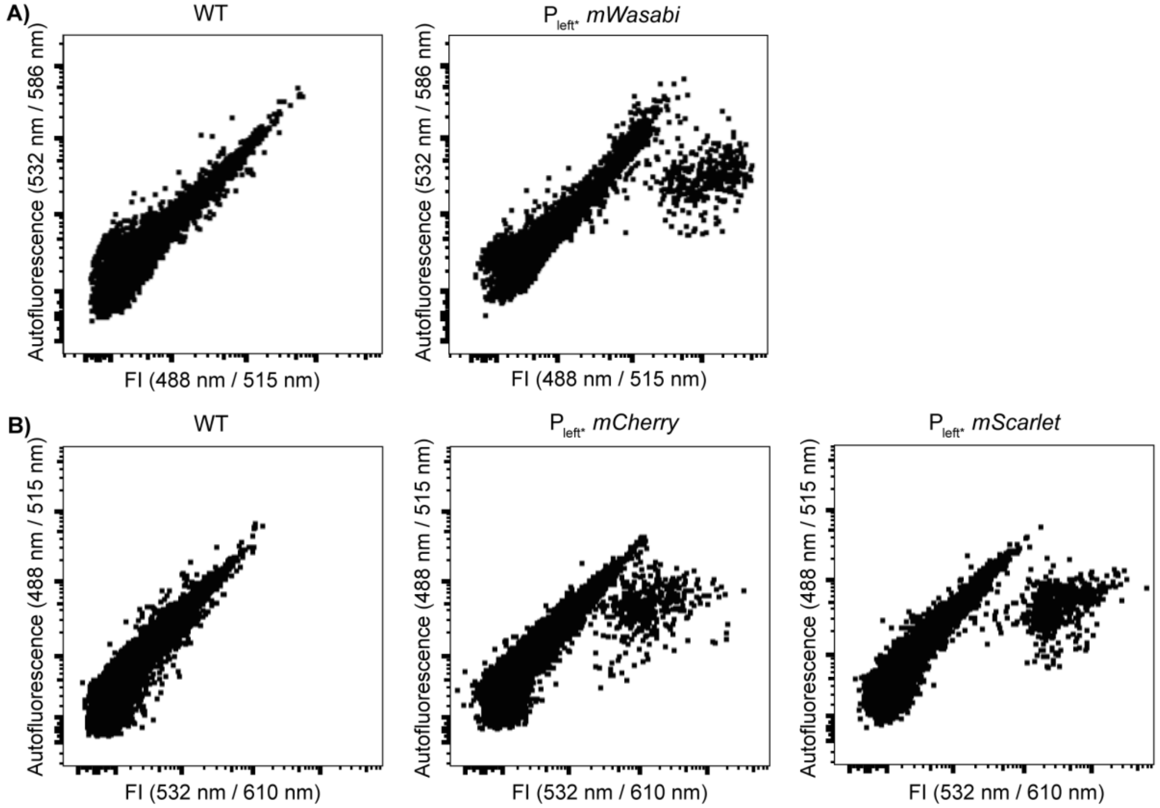

Figure S7. Flow cytometric detection of fluorescent reporter strains in lungs of WT mice. Flow cytometric analysis of **(A)** *Mtb* HN878 (L5 *attB*::P_{left*} *mWasabi*) at 515 nm and **(B)** *Mtb* HN878 (L5 *attB*::P_{left*} *mCherry*) and *Mtb* HN878 (L5 *attB*::P_{left*} *mScarlet*) signal at 610 nm in lung single-cell suspensions from WT B6.SJL (CD45.1/1) 37 days post-infection with 50 CFU. Data are representative of one experiment with 2-3 mice per fluorescent reporter strain.

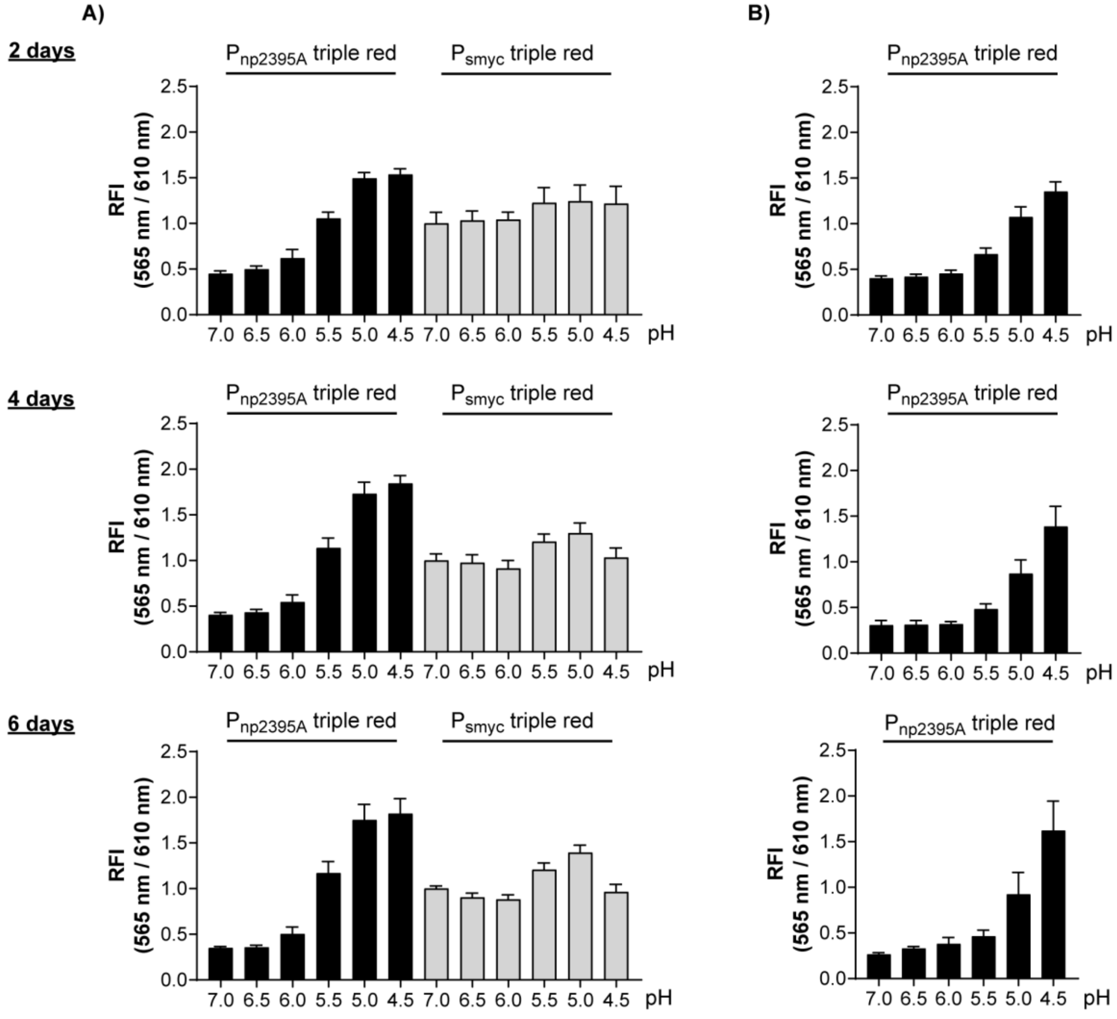

Figure S8. The pH-responsiveness of *Mtb* (Giles *attB*::P$_{\mathbf{np2395A}}$ triple red) is Mg^{2+}- and time-dependent. Relative fluorescence intensities (RFIs) of the reporter strain *Mtb* (Giles *attB*::P$_{np2395A}$ triple red) and the control strain *Mtb* (Giles *attB*::P$_{smyc}$ triple red) detected at three different time points (2, 4, and 6 days) in media with various pH values and either **(A)** high Mg^{2+} concentration (500 µM) or **(B)** low Mg^{2+} concentration (10 µM). FIs were detected during the logarithmic growth phase of the strains, analyzed relative to their optical densities at 600 nm, and normalized with FI of *Mtb* (pOLYG P$_{smyc}$ *mCherry*) equal to 1. Data are representative of at least seven biological replicates; the error bars indicate standard deviation.

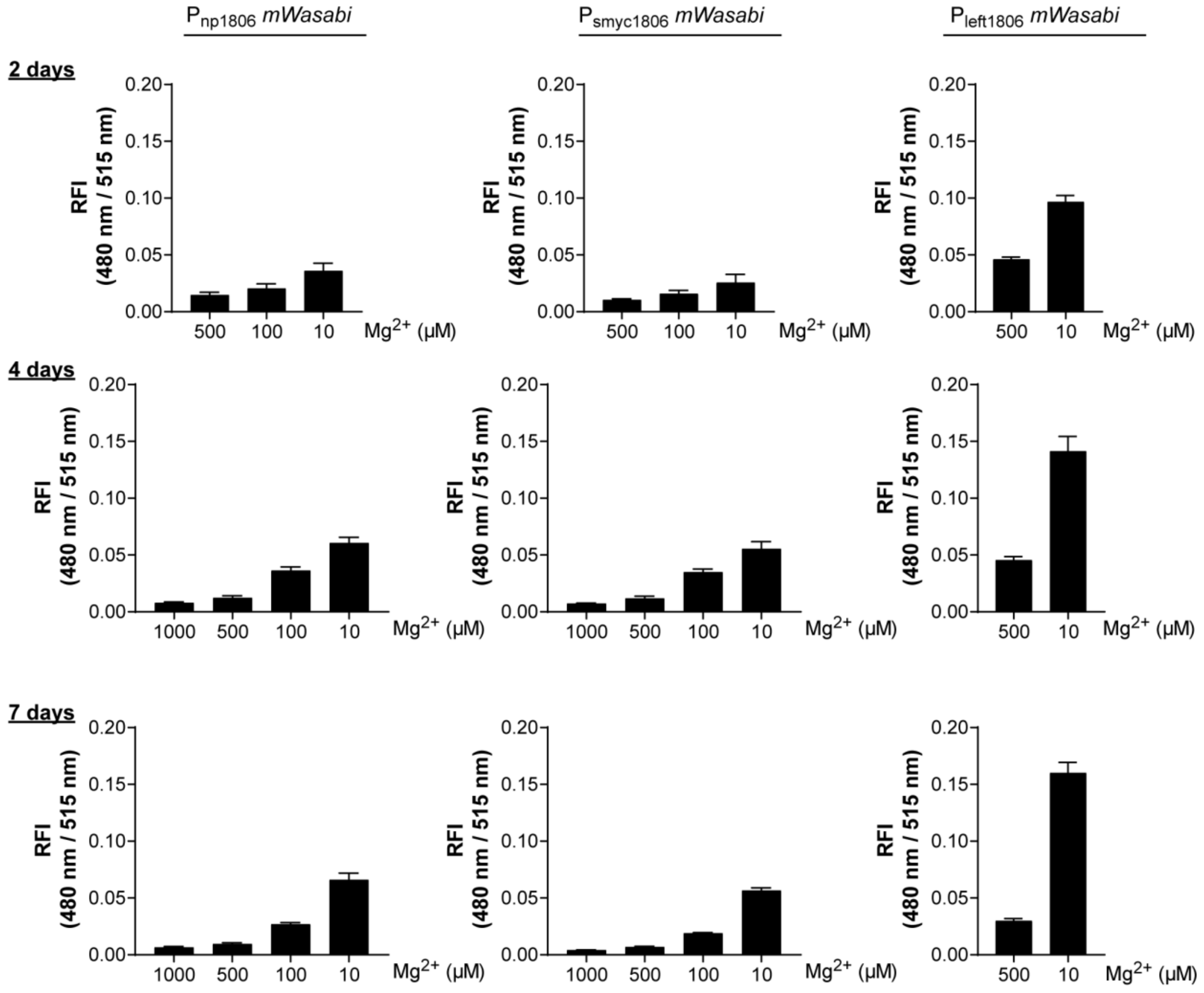

Figure S9. Mg^{2+} responsiveness was maintained when the riboswitch upstream of *rv1806* was fused to the promoters P_{smyc} or P_{left}. Relative fluorescence intensities (RFIs) of the reporter strains *Mtb* (Giles *attB*::P_{np1806} *mWasabi*), *Mtb* (Giles *attB*::$P_{smyc1806}$ *mWasabi*), and *Mtb* (Giles *attB*::$P_{left1806}$ *mWasabi*) detected at three different time points (2, 4, and 7 days) in media with pH 7.0 and various Mg^{2+} concentrations. FIs were measured during the logarithmic growth phase of the strains, analyzed relative to their optical densities at 600 nm, and normalized with FI of *Mtb* (pOLYG P_{smyc} *eGFP*) equal to 1. Data are representative of at least four biological replicates; the error bars indicate standard deviation.

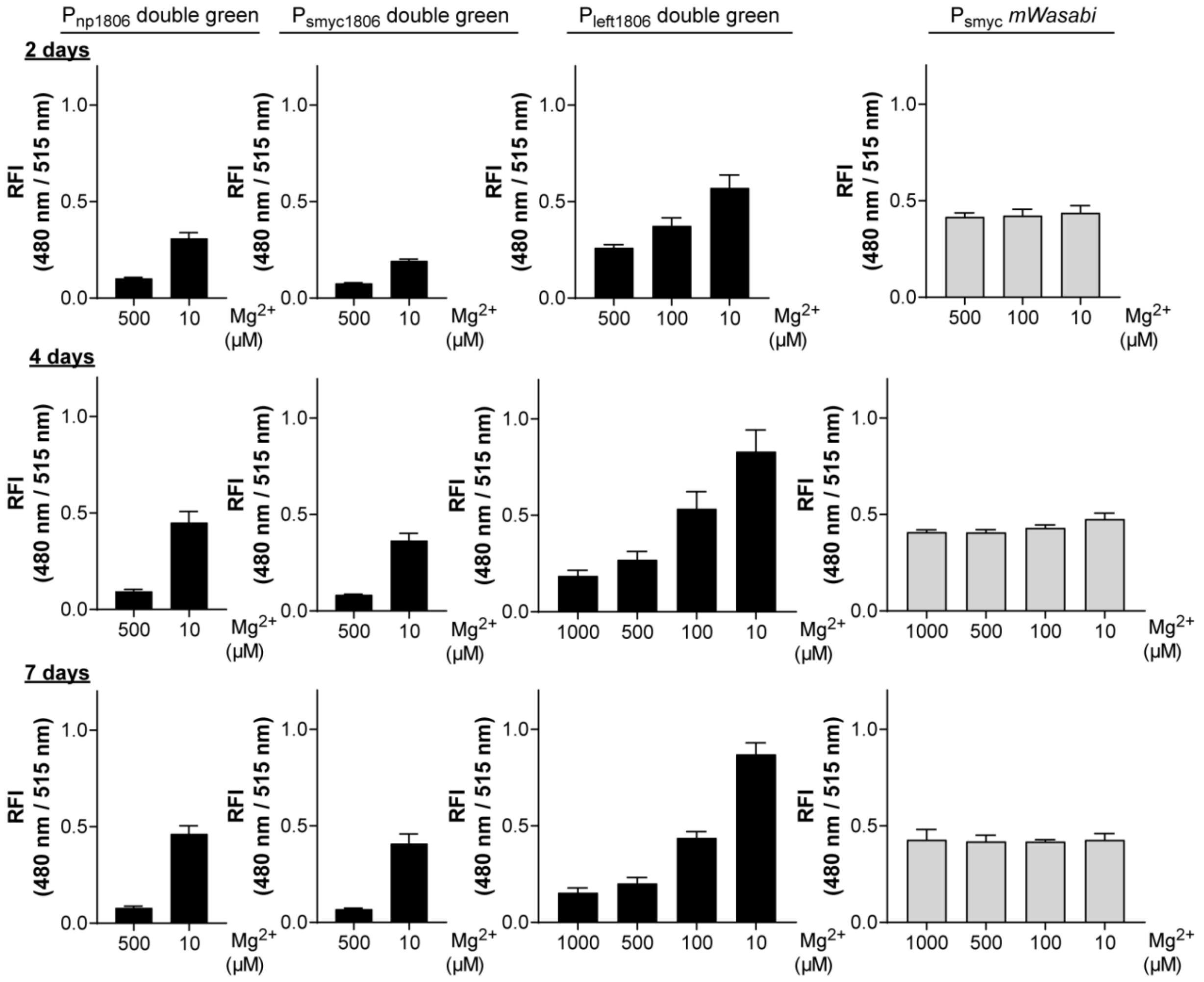

Figure S10. Mg^{2+} responsiveness was maintained when multicistronic constructs were introduced. Relative fluorescence intensities (RFIs) of the reporter strains *Mtb* (Giles *attB*::P_{np1806} double green), *Mtb* (Giles *attB*::$P_{smyc1806}$ double green), and *Mtb* (Giles *attB*::$P_{left1806}$ double green), as well as the control strain *Mtb* (Giles *attB*::P_{smyc} *mWasabi*), detected at three different time points (2, 4, and 7 days) in media with pH 7.0 and various Mg^{2+} concentrations. FIs were measured during the logarithmic growth phase of the strains, analyzed relative to their optical densities at 600 nm, and normalized with FI of *Mtb* (pOLYG P_{smyc} *eGFP*) equal to 1. Data are representative of at least four biological replicates; the error bars indicate standard deviation.

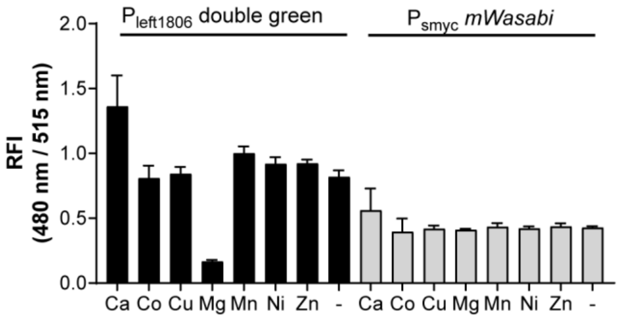

	MIC	Fluorescence assay
$CaCl_2$	>2.5 mM	1 mM
$CoSO_4$	312.5 µM	100 µM
$CuSO_4$	156 µM	40 µM
$MgSO_4$	>2.5 mM	1 mM
$MnSO_4$	625 µM	150 µM
$NiSO_4$	1.25 mM	300 µM
$ZnSO_4$	>2.5 mM	1 mM

Figure S11. Other divalent ions do not influence the riboswitch-dependent fluorescence signal. (A) Relative fluorescence intensities (RFIs) of the reporter strain *Mtb* (Giles *attB*::$P_{left1806}$ double green) and the control strain *Mtb* (Giles *attB*::P_{smyc} *mWasabi*) detected after 6 days of incubation at a Mg^{2+} concentration of 10 µM, pH 7.0 and addition of calcium chloride ($CaCl_2$; 1 mM), cobalt(II) sulfate ($CoSO_4$; 100 µM), copper(II) sulfate ($CuSO_4$; 40 µM), magnesium sulfate ($MgSO_4$; 1 mM), manganese(II) sulfate ($MnSO_4$; 150 µM), nickel(II) sulfate ($NiSO_4$; 300 µM), or zinc sulfate ($ZnSO_4$; 1 mM). FIs were analyzed relative to their optical densities at 600 nm and normalized with FI of *Mtb* (pOLYG P_{smyc} *eGFP*) equal to 1. Data are representative of eight biological replicates; the error bars indicate standard deviation. **(B)** The minimal inhibitory concentrations (MIC) of different divalent ions on *Mtb* H37Rv growth detected after 14 days of incubation (column 2). Concentrations chosen for the fluorescence assay based on the MIC data (column 3).

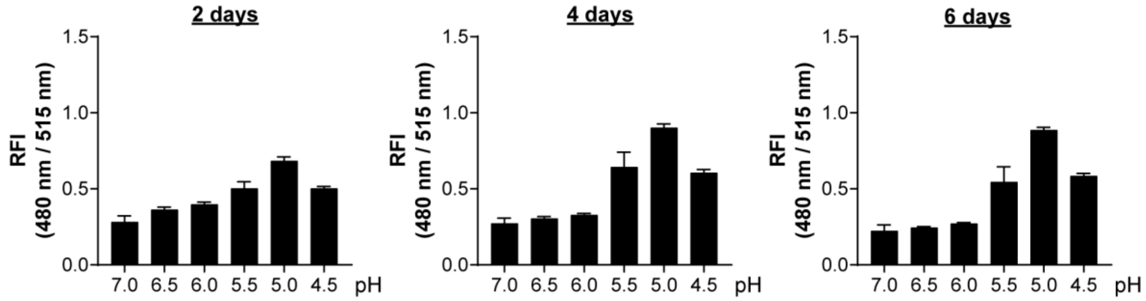

Figure S12. The fluorescence intensity of the Mg^{2+} reporter *Mtb* (Giles *attB*::$P_{left1806}$ double green) increases under acid conditions. Relative fluorescence intensities (RFIs) of the reporter strain *Mtb* (Giles *attB*::$P_{left1806}$ double green) detected at three different time points (2, 4, and 6 days) in media with a Mg^{2+} concentration of 500 µM and various pH values. FIs were measured during the logarithmic growth phase of the strain, analyzed relative to the optical density at 600 nm, and normalized with FI of *Mtb* (pOLYG P_{smyc} *eGFP*) equal to 1. Data are representative of at least six biological replicates; the error bars indicate standard deviation.

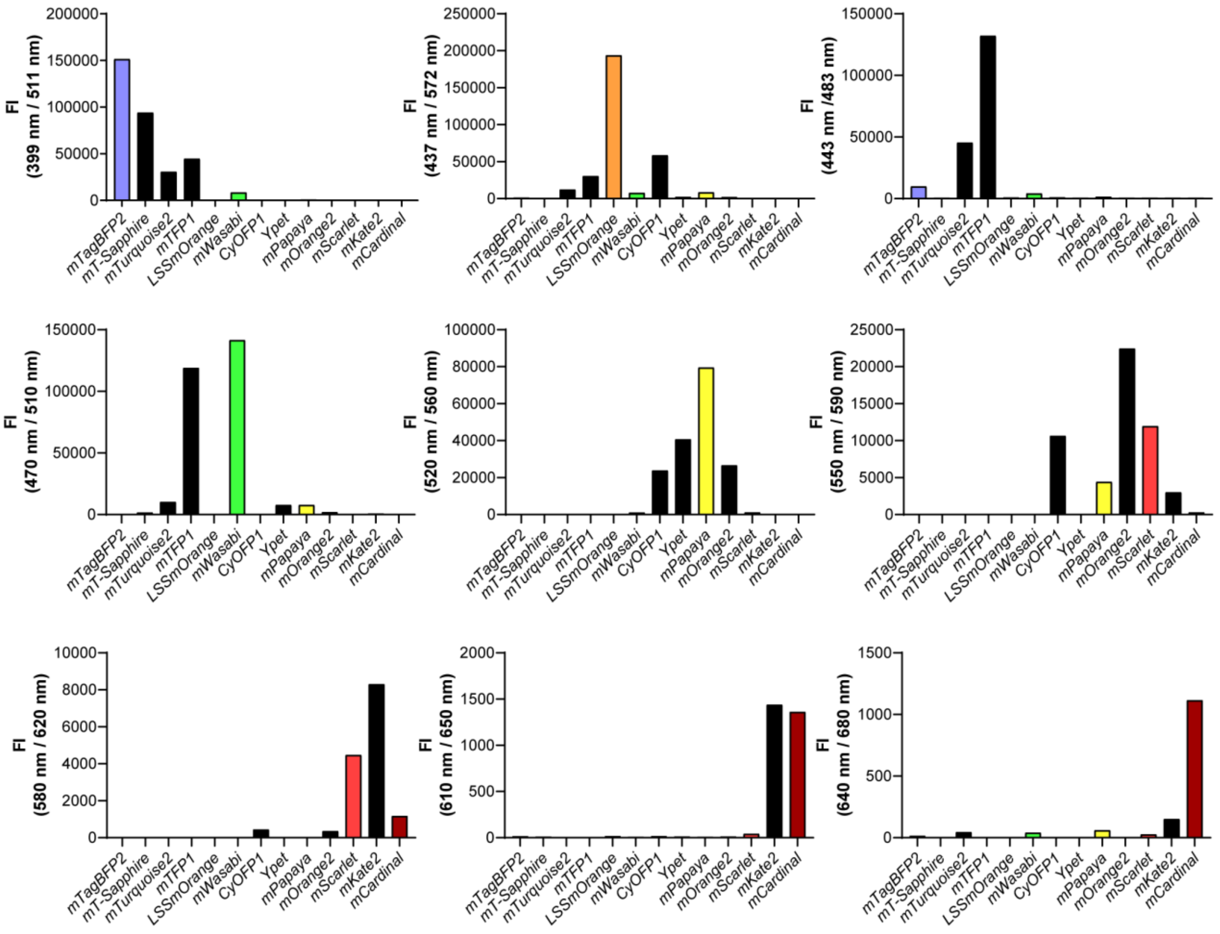

Figure S13. Screening a library of fluorescent proteins identified four additional *Mtb* strains that may be used in combination with *Mtb* (L5 *attB*::P$_{smyc}$ *mScarlet*) and *Mtb* (L5 *attB*::P$_{smyc}$ *mWasabi*). Fluorescence intensities (FIs) of *Mtb* H37Rv strains expressing different fluorescent proteins under the control of the *smyc* promoter analyzed across a range of excitation and emission wavelengths. FIs were detected during the logarithmic growth phase of the strains and analyzed relative to their optical densities at 600 nm. Data are representative of at least two biological replicates.

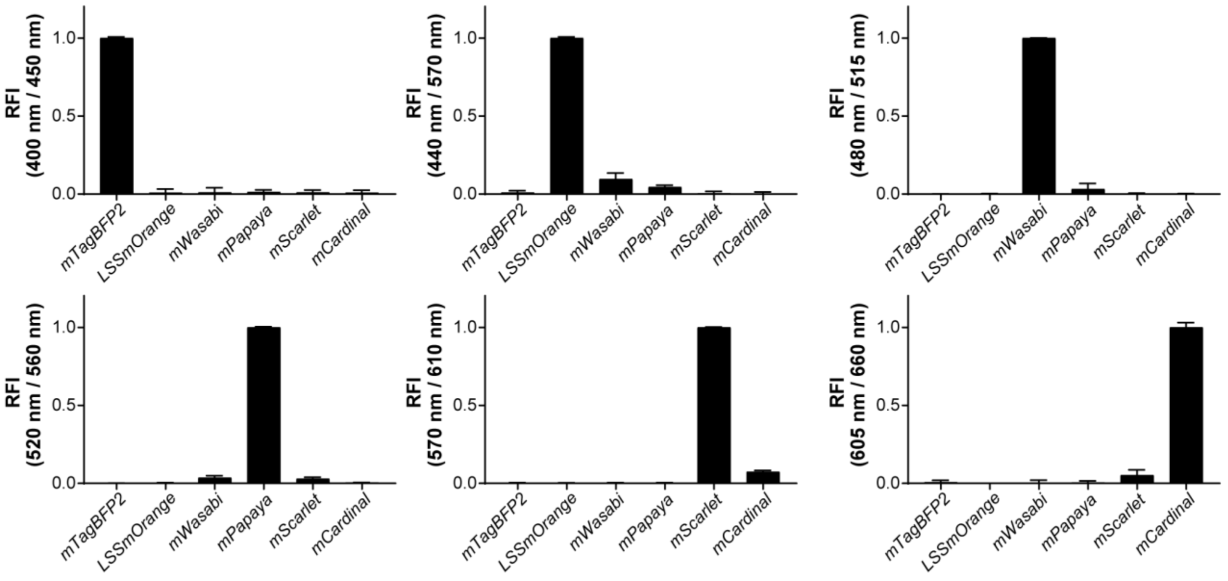

Figure S14. Successful combination of six fluorescent proteins with minimal spectral crosstalk. Relative fluorescence intensities (RFIs) of six *Mtb* H37Rv strains expressing different fluorescent proteins under the control of the *smyc* promoter analyzed at their respective optimal excitation and emission wavelengths. FIs were detected during the logarithmic growth phase of the strains, analyzed relative to their optical densities at 600 nm, and normalized with the highest FI equal to 1. Data are representative of at least four biological replicates; the error bars indicate standard deviation.

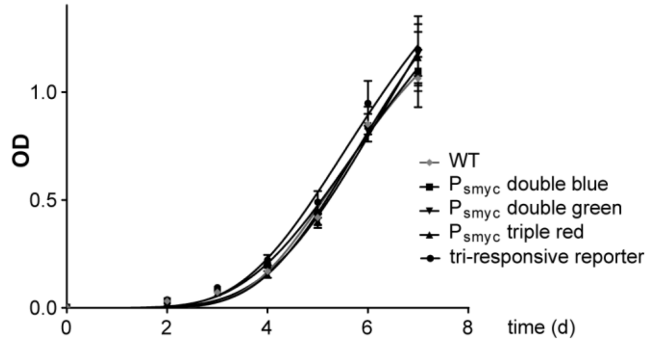

Figure S15. The tri-responsive reporter strain showed satisfying growth characteristics. Growth rates of an *Mtb* H37Rv WT strain, *Mtb* (L5 *attB*::P_{smyc} double blue), *Mtb* (Giles *attB*::P_{smyc} double green), *Mtb* (Giles *attB*::P_{smyc} triple red), and the tri-responsive reporter strain in 7H9 medium were detected by OD_{650}. Data are representative of three biological replicates; the error bars indicate standard deviation.

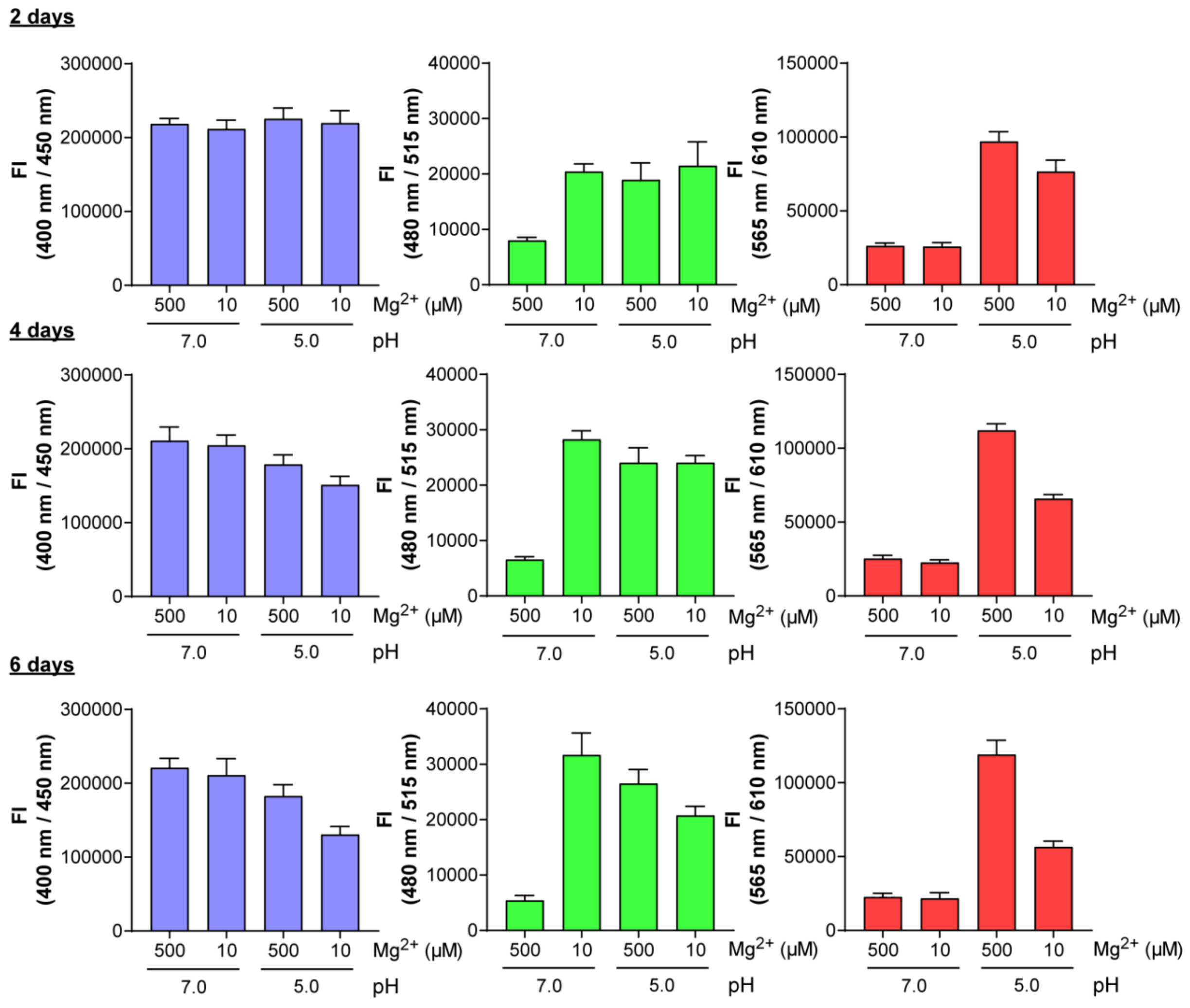

Figure S16. The tri-responsive reporter strain enables simultaneous visualization of three conditions in parallel using fluorescence signals at three different wavelengths: protein expression (blue), Mg^{2+} concentration (green), and pH (red). Fluorescence intensities (FIs) at optimal excitation and emission wavelengths of the three fluorescent proteins detected after 2, 4, and 6 days of growth in neutral (pH 7.0) and acidic pH (pH 5.0), as well as high (500 µM) and low (10 µM) Mg^{2+} concentrations. FIs were measured during the logarithmic growth phase of the strain and analyzed relative to the optical densities at 600 nm. Data are representative of sixteen biological replicates; the error bars indicate standard deviation.

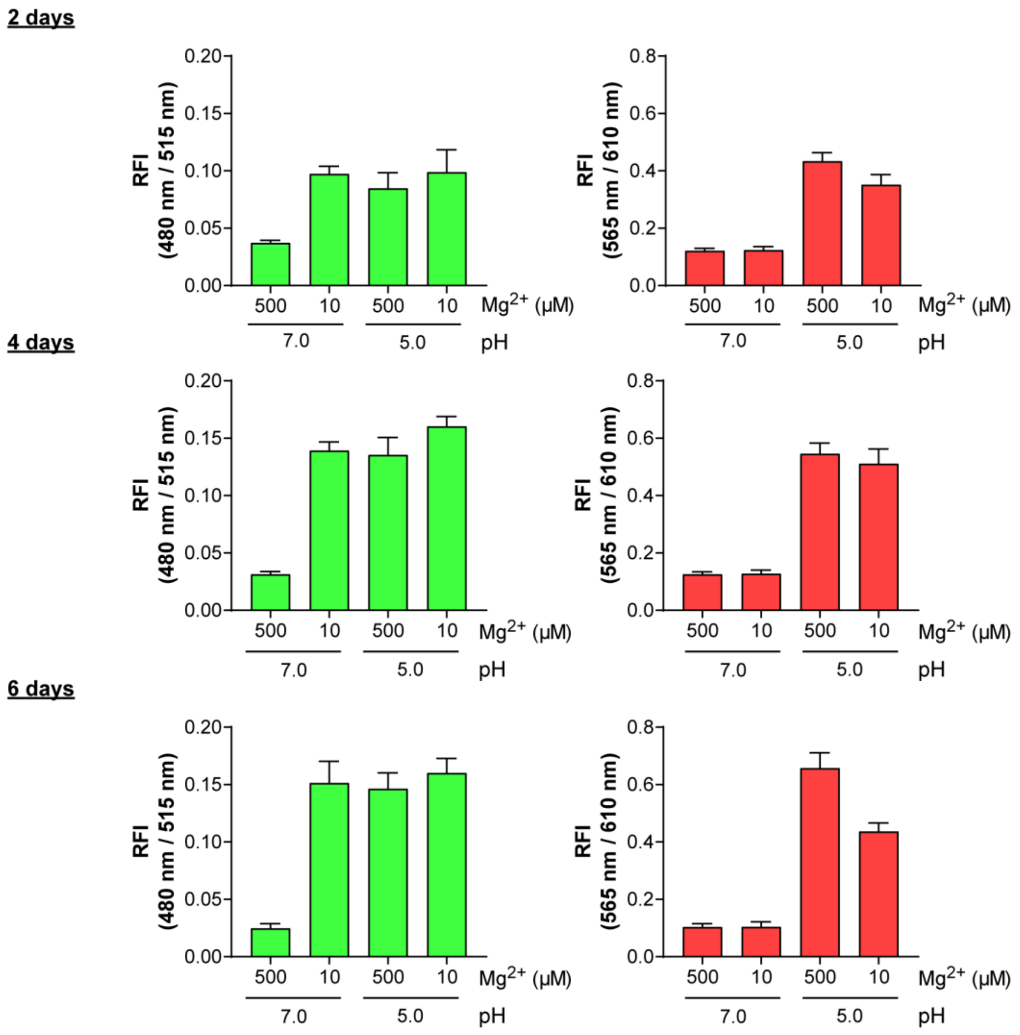

Figure S17. The Mg^{2+}- and pH-dependent response of the tri-responsive reporter strain analyzed relative to changes in its protein expression level. Fluorescence intensities (FIs) at 480 nm/515 nm (green) and 565 nm/610 nm (red) detected after 2, 4, and 6 days of growth in neutral (pH 7.0) and acidic pH (pH 5.0), as well as high (500 µM) and low (10 µM) Mg^{2+} concentrations, and depicted relative to changes in protein expression levels (400 nm/450 nm, blue). FIs were measured during the logarithmic growth phase of the strain and analyzed relative to the optical densities at 600 nm. Data are representative of sixteen biological replicates; the error bars indicate standard deviation.

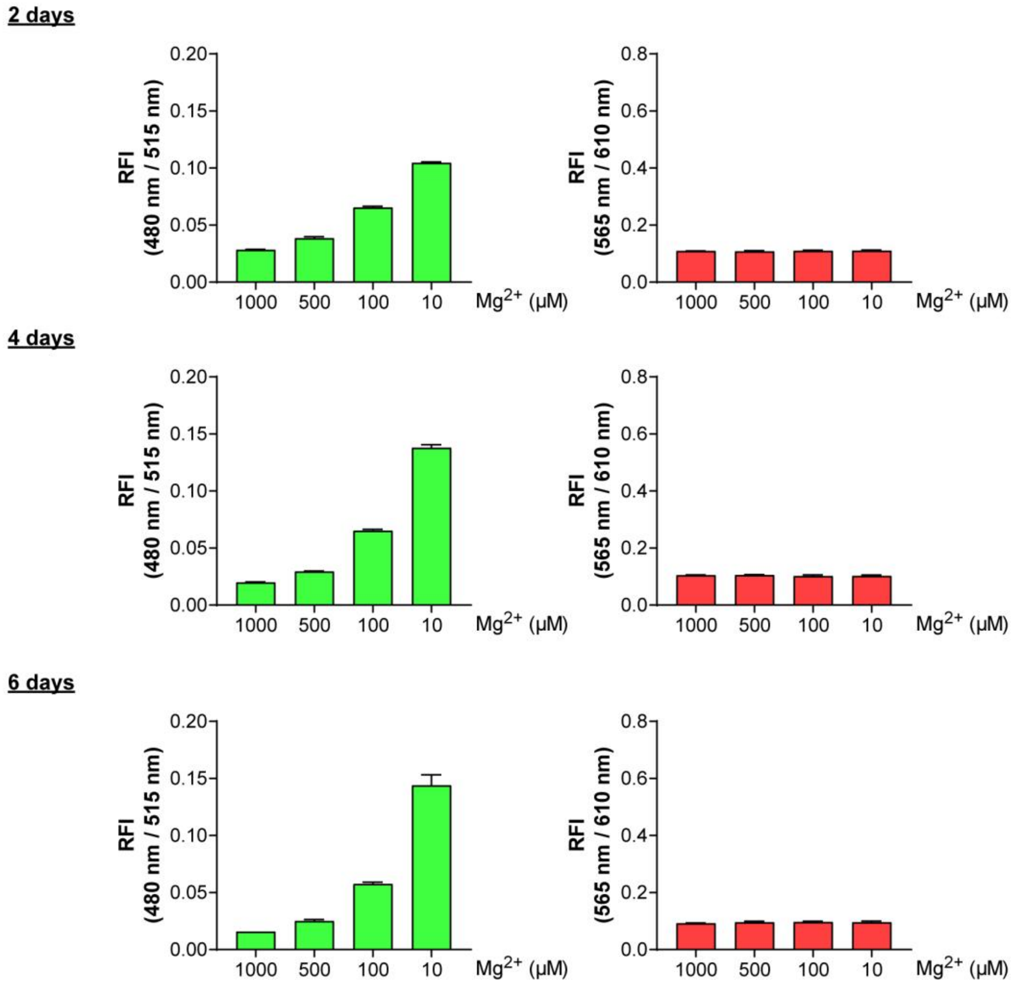

Figure S18. Mg^{2+}-dependent fluorescence response of the tri-responsive reporter strain relative to its protein expression levels. Fluorescence intensities (FIs) at 480 nm/515 nm (green) and 565 nm/610 nm (red) detected after 2, 4, and 6 days of growth at pH 7.0 and various Mg^{2+} concentrations and depicted relative to changes in protein expression levels (400 nm/450 nm, blue). FIs were measured during the logarithmic growth phase of the strain and analyzed relative to the optical densities at 600 nm. Data are representative of four biological replicates; the error bars indicate standard deviation.

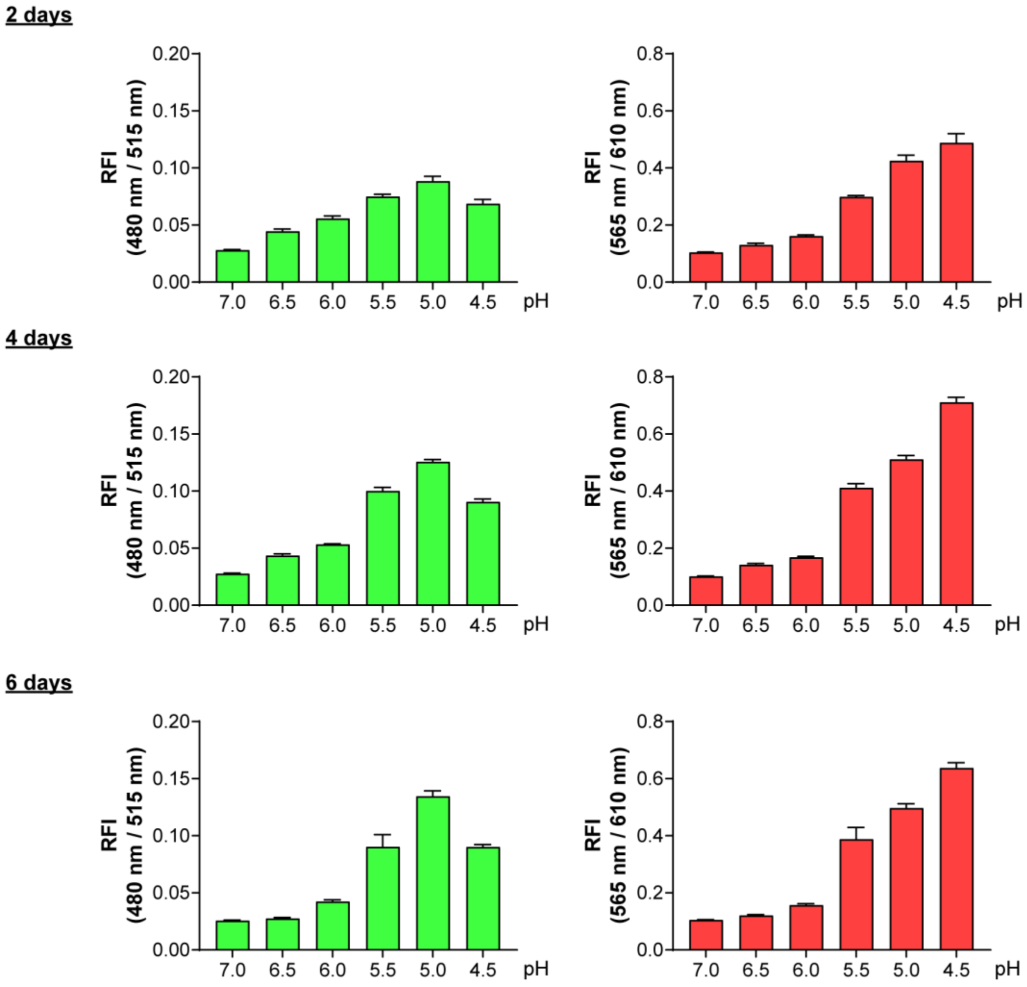

Figure S19. pH-dependent fluorescence response of the tri-responsive reporter strain relative to its protein expression levels. FIs at 480 nm/515 nm (green) and 565 nm/610 nm (red) detected after 2, 4, and 6 days of growth at Mg^{2+} concentrations of 500 µM and different pH values and depicted relative to changes in protein expression levels (400 nm/450 nm, blue). FIs were measured during the logarithmic growth phase of the strain and analyzed relative to the optical densities at 600 nm. Data are representative of four biological replicates; the error bars indicate standard deviation.

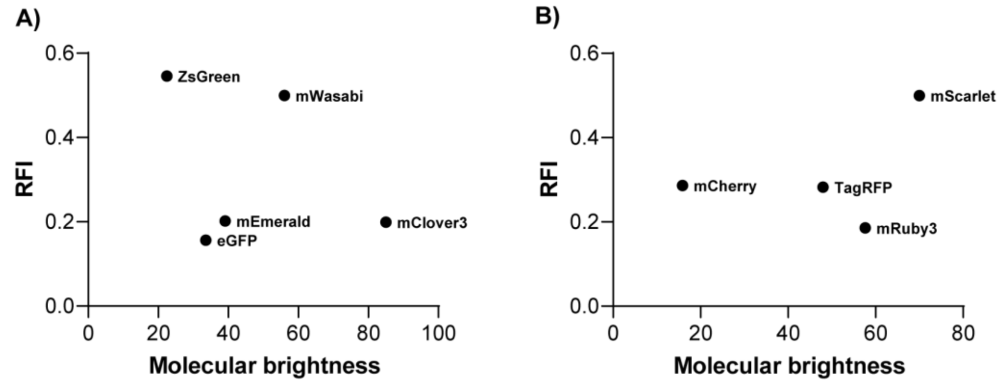

Figure S20. Molecular brightness of a fluorescent protein does not correlate with the relative fluorescence intensity (RFI) of the respective fluorescent *Mtb* strain. The known molecular brightness levels and the newly detected RFIs of *Mtb* expressing different **(A)** green fluorescent proteins and **(B)** red fluorescent proteins were graphed.

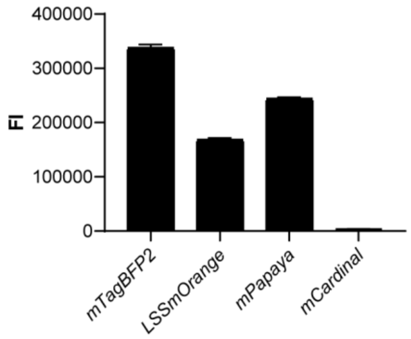

Figure S21. *Mtb* bacteria expressing mTagBFP2 had the strongest readout signal in this study. Fluorescence intensities (FIs) of *Mtb* (L5 *attB*::P_{smyc} *mTagBFP2*), *Mtb* (L5 *attB*::P_{smyc} *LSSmOrange*), *Mtb* (L5 *attB*::P_{smyc} *mPapaya*), and Mtb (L5 *attB*::P_{smyc} *mCardinal*) detected at the respective optimal excitation and emission wavelengths: 400 nm/450 nm, 440 nm/570 nm, 520 nm/560nm, and 605 nm/660 nm. FIs were detected during the logarithmic growth phase of the strains and analyzed relative to the optical densities at 600 nm. Data are representative of two biological replicates; the error bars indicate standard deviation.

3 Supplementary Tables

Table S1. Bacterial strains used in this work.

Strain	Genotype	Source or reference
E. coli (NEB5α)	fhuA2 Δ(argF-lacZ)U169 phoA glnV44 Φ80 Δ(lacZ)M15 gyrA96 recA1 relA1 endA1 thi-1 hsdR17	NEB
Mtb H37Rv		ATCC 25618
Mtb (pOLYG P_{smyc} *eGFP*)	pOLYG, Hyg^r, P_{smyc}-*eGFP* (the non His-tagged promoter-gene element was constructed within pL5 and transferred into *XbaI*/*HindIII* restriction sites of pOLYG)	Lab strain
Mtb (pOLYG P_{smyc} *mCherry*)	pOLYG, Hyg^r, P_{smyc}-*mCherry* (the non His-tagged promoter-gene element was constructed within pL5 and transferred into *XbaI*/*HindIII* restriction sites of pOLYG)	Lab strain
Mtb (L5 *attB*::P_{smyc} *eGFP*)	*attB*::pL5, Kan^r, P_{smyc}-*HiseGFP*	This study
Mtb (L5 *attB*::P_{smyc} *mClover3*)	*attB*::pL5, Kan^r, P_{smyc}-*HismClover3*	This study
Mtb (L5 *attB*::P_{smyc} *mEmerald*)	*attB*::pL5, Kan^r, P_{smyc}-*HismEmerald*	This study
Mtb (L5 *attB*::P_{smyc} *mWasabi*)	*attB*::pL5, Kan^r, P_{smyc}-*HismWasabi*	This study
Mtb (L5 *attB*::P_{smyc} *ZsGreen*)	*attB*::pL5, Kan^r, P_{smyc}-*HisZsGreen*	This study
Mtb (L5 *attB*::P_{smyc} *mCherry*)	*attB*::pL5, Kan^r, P_{smyc}-*HismCherry*	This study
Mtb (L5 *attB*::P_{smyc} *mRuby3*)	*attB*::pL5, Kan^r, P_{smyc}-*HismRuby3*	This study
Mtb (L5 *attB*::P_{smyc} *TagRFP*)	*attB*::pL5, Kan^r, P_{smyc}-*HisTagRFP*	This study
Mtb (L5 *attB*::P_{smyc} *mScarlet*)	*attB*::pL5, Kan^r, P_{smyc}-*HismScarlet*	This study
Mtb (L5 *attB*::P_{smyc} double green)	*attB*::pL5, Kan^r, P_{smyc}-*HismWasabi-RBS-HisCOmWasabi*	This study
Mtb (L5 *attB*::P_{smyc} double red)	*attB*::pL5, Kan^r, P_{smyc}-*HismScarlet-RBS-HisCOmScarlet*	This study
Mtb (L5 *attB*::P_{smyc} triple red)	*attB*::pL5, Kan^r, P_{smyc}-*HismScarlet-RBS-HisCOmScarlet-RBS-HismRuby3*	This study
Mtb (L5 *attB*::P_{smyc} *eGFP*)	*attB*::pL5, Kan^r, P_{smyc}-*eGFP*	This study
Mtb (L5 *attB*::P_{hsp60} *eGFP*)	*attB*::pL5, Kan^r, P_{hsp60}-*eGFP*	This study
Mtb (L5 *attB*::P_{G13} *eGFP*)	*attB*::pL5, Kan^r, P_{G13}-*eGFP*	This study
Mtb (L5 *attB*::P_{msp12} *eGFP*)	*attB*::pL5, Kan^r, P_{msp12}-*eGFP*	This study
Mtb (L5 *attB*::P_{MOP} *eGFP*)	*attB*::pL5, Kan^r, P_{MOP}-*eGFP*	This study
Mtb (L5 *attB*::P_{left} *eGFP*)	*attB*::pL5, Kan^r, P_{left}-*eGFP*	This study
Mtb (L5 *attB*::P_{left*} *eGFP*)	*attB*::pL5, Kan^r, P_{left*}-*eGFP*	This study
Mtb (Giles *attB*::P_{smyc} *mWasabi*)	*attB*::pGiles, Hyg^r, P_{smyc}-*HismWasabi*	This study
Mtb (Giles *attB*::$P_{np2395A}$ triple red)	*attB*::pGiles, Hyg^r, $P_{np2395A}$-*HismScarlet*-RBS-*HisCOmScarlet*-RBS-*HismRuby3*	This study
Mtb (Giles *attB*::P_{np1535} *mWasabi*)	*attB*::pGiles, Hyg^r, P_{np1535}-*HismWasabi*	This study
Mtb (Giles *attB*::P_{np1806} *mWasabi*)	*attB*::pGiles, Hyg^r, P_{np1806}-*HismWasabi*	This study
Mtb (Giles *attB*::P_{np1806} double green)	*attB*::pGiles, Hyg^r, P_{np1806}-*HismWasabi-RBS-HisCOmWasabi*	This study
Mtb (Giles *attB*::$P_{smyc1806}$ *mWasabi*)	*attB*::pGiles, Hyg^r, $P_{smyc1806}$-*HismWasabi*	This study
Mtb (Giles *attB*::$P_{smyc1806}$ double green)	*attB*::pGiles, Hyg^r, $P_{smyc1806}$-*HismWasabi-RBS-HisCOmWasabi*	This study

Mtb (Giles *attB*::$P_{left1806}$ *mWasabi*)	*attB*::pGiles, Hyg^{r}, $P_{left1806}$-*HismWasabi*	This study
Mtb (Giles *attB*::$P_{left1806}$ double green)	*attB*::pGiles, Hyg^{r}, $P_{left1806}$-*HismWasabi-RBS-HisCOmWasabi*	This study
Mtb (L5 *attB*::P_{smyc} *mTagBFP2*)	*attB*::pL5, Kan^{r}, P_{smyc}-*HismTagBFP2*	This study
Mtb (L5 *attB*::P_{smyc} *mT-Sapphire*)	*attB*::pL5, Kan^{r}, P_{smyc}-*HismT-Sapphire*	This study
Mtb (L5 *attB*::P_{smyc} *mTurquoise2*)	*attB*::pL5, Kan^{r}, P_{smyc}-*HismTurquoise2*	This study
Mtb (L5 *attB*::P_{smyc} *mTFP1*)	*attB*::pL5, Kan^{r}, P_{smyc}-*HismTFP1*	This study
Mtb (L5 *attB*::P_{smyc} *LSSmOrange*)	*attB*::pL5, Kan^{r}, P_{smyc}-*HisLSSmOrange*	This study
Mtb (L5 *attB*::P_{smyc} *Ypet*)	*attB*::pL5, Kan^{r}, P_{smyc}-*HisYpet*	This study
Mtb (L5 *attB*::P_{smyc} *mPapaya*)	*attB*::pL5, Kan^{r}, P_{smyc}-*HismPapaya*	This study
Mtb (L5 *attB*::P_{smyc} *mOrange2*)	*attB*::pL5, Kan^{r}, P_{smyc}-*HismOrange2*	This study
Mtb (L5 *attB*::P_{smyc} *mKate2*)	*attB*::pL5, Kan^{r}, P_{smyc}-*HismKate2*	This study
Mtb (L5 *attB*::P_{smyc} *mCardinal*)	*attB*::pL5, Kan^{r}, P_{smyc}-*HismCardinal*	This study
Mtb (L5 *attB*::P_{smyc} double blue)	*attB*::pL5, Kan^{r}, P_{smyc}-*HismTagBFP2-RBS-HisCOmTagBFP2*	This study
Mtb (tri-responsive reporter)	*attB*::pL5, Kan^{r}, P_{smyc}-*HismTagBFP2-RBS-HisCOmTagBFP2;*<br>*attB*::pGiles, Hyg^{r}, $P_{np2395A}$-*HismScarlet-RBS-HisCOmScarlet-RBS-HismRuby3*, $P_{left1806}$-*HismWasabi-RBS-HisCOmWasabi*	This study

Table S2 Buffer, media and supplements.

Name	Ingredients
TFB-I	Potassium acetate (2.9 g), manganese chloride (6.3 g), calcium chloride (1.1 g), and glycerol (150 ml) were dissolved in dist. water (up to 1.0 l), and filter sterilized.
TFB-II	MOPS buffer (1 M, pH 7.0; 10 ml), calcium chloride (8.3 g), potassium chloride (746 mg), and glycerol (150 ml) were dissolved in dist. water (up to 1.0 l), and filter sterilized.
Chelex buffer	Tris HCl (1 M, pH 8; 1 ml), EDTA (500 mM, pH 8; 200 µl), sodium azide (100 mg), and BT Chelex 100 resin (BioRad; 20 ml) were added to dist. water (up to 100 ml).
7H9 ADGNTw	Glycerol (2.0 g), D-glucose (2.0 g), sodium chloride (850 mg), BSA (5.0 g), and 20 % Tween 80 (2.5 ml) were dissolved in dist. water (100 µl), added to 7H9 medium (900 ml), and filter sterilized.
7H11 OADGN	Glycerol (5.0 g), D-glucose (2.0 g), sodium chloride (850 mg), BSA (5.0 g), and oleic acid (60 µl) were dissolved in dist. water (100 µl), filter sterilized, and added to autoclaved 7H11 agar (900 ml).
Sauton’s medium	Potassium dihydrogen phosphate (500 mg), potassium sulfate (725 mg), citric acid (2.0 g), ferric ammonium citrate (50 mg), L-asparagine (4.0 g), 1 % zinc sulfate solution (100 µl), glycerol (20.0 g), and 20 % Tyloxapol (2.5 ml) were dissolved in dist. water (up to 1.0 l), adjusted to the desired pH with sodium hydroxide (10 M) and to the respective Mg^{2+} concentration with a magnesium sulfate solution (500 mM), and filter sterilized.

Table S3. Vectors used for cloning and suicide plasmids for *Mtb* transformation.

Plasmid	Description	Source or reference
pOLYG	Hyg^r	(Ó Gaora et al., 1997)
pMV361	pMV306, Kan^r, *int L5*, P_{hsp60}	(Stover et al., 1991)
pL5	pL5, Kan^r, P_{smyc}, *NcoI*	This study
pML1357	pML603, Hyg^r, *int Giles*, P_{smyc}-*GFP*, *xylE*	Addgene #32378 (Huff et al., 2010)
pGiles	pML1357, Hyg^r, $P_{smyc;}$ *NcoI*, *AvrII*, *BglII*, *PstI*, *ApaI*, *BmtI*	This study
pBS-Int	pBluescript, Amp^r, *int L5*	Addgene #50000 (Springer et al., 2001)
pUC19	pUC6, Amp^r	Addgene #50005 (Norrander et al., 1983)
pUC19-GI	pUC6, Amp^r, *int Giles*	This study
pMSP12::GFP	pFPV27, Kan^r, P_{msp12}-*eGFP*	Addgene #30167 (Chan et al., 2002)
pKanCMV-mClover3-mRuby3	pKanCMV, Amp^r, P_{CMV}-*mClover3-mRuby3*	Addgene #74252 (Bajar et al., 2016)
mEmerald-pBAD	pBAD, Amp^r, *HismEmerald*	Addgene #54220
pTEC15	pFPV27, Hyg^r, P_{msp12}-*mWasabi*	Addgene #30174 (Takaki et al., 2013)
pCAG-loxPSTOPloxP-ZsGreen	pDIRECT, Amp^r, P_{CAG}-loxPSTOPloxP-*ZsGreen*	Addgene #51269 (Hermann et al., 2014)
pCHERRY10	pSMT3, Hyg^r, P_{G13}-*mCherry*	Addgene #24664 (Carroll et al., 2010)
pEB2-TagRFP	pEB2, Kan^r, P_{proC}-*TagRFP*	Addgene #103997 (Balleza et al., 2018)
pEB2-mScarlet	pEB2, Kan^r, P_{proC}-*mScarlet*	Addgene #104006 (Balleza et al., 2018)
pTEC16	pFPV27, Hyg^r, P_{msp12}-*mOrange2*	Addgene #30175 (Davis et al., 2013)
pMH29	P_{MOP}, Kan^r	(George et al., 1995)
mTagBFP2-pBAD	pBAD, Amp^r, *HismTagBFP2*	Addgene #54572 (Subach et al., 2011)
mT-Sapphire-pBAD	pBAD, Amp^r, *HismT-Sapphire*	Addgene #54571 (Ai et al., 2008)
mScarlet-I-mTurquoise2	pEGFP-N4, Kan^r, P_{CMV}-*mScarletI-mTurquoise2*	Addgene #98839 (Ai et al., 2008)
mTFP1-pBAD	pBAD, Amp^r, *HismTFP1*	Addgene #54553 (Ai et al., 2006)
pLSSmOrange-mKate2 caspase-3 biosensor	pN1, Kan^r, P_{CMV}-*LSSmOrange-DEVD-mKate2*	Addgene #37132 (Shcherbakova et al., 2012)
pNCS-CyOFP	pNCS, Amp^r, P_{T7}-*HisCyOFP*	Addgene #74278 (Chu et al., 2016)
YPet-N1	pN1, Kan^r, *YPet*	Addgene #54637 (Nguyen et al., 2005)
mPapaya-pBAD	pBAD, Amp^r, *HismPapaya*	Addgene #54834 (Hoi et al., 2013)
pNCS-mCardinal2	pNCS, Amp^r, *mCardinal*	Addgene #52631 (Chu et al., 2014)

Table S4. Oligonucleotides used in this study. Restriction sites are underlined.

#	Primer name	Sequence (5' → 3') (Restriction site)<br>Application
1	pMV361_Fw	GTGACCATGGCAGAGCTCGAATTCGAAGCTTATCGATG (*NcoI*)<br>Amplifies pMV361; introduces *NcoI*
2	pMV361_Rv	GTGACCATGGCGAAGTGATTCCTCCGGATCG (*NcoI*)<br>Amplifies pMV361; introduces *NcoI*
3	pMV306_dI_Fw	CTCTGGATCCTCCCAAGGACACTGAGTC (*BamHI*)<br>Deletes *L5 integrase* from pMV361
4	pMV306_dI_Rv	CTCTGGATCCCTAGGTCTCCTCTCGTCG (*BamHI*)<br>Deletes *L5 integrase* from pMV361
5	P_{smyc}_Fw	GTGTTCTAGAGGATCGTCGGCACC (*XbaI*)<br>Transfers P_{smyc} into pL5
6	P_{smyc}_Rv	GCGCCCATGGAGATACCTCCTTAATTAAGCATGCGGATC (*NcoI*)<br>Transfers P_{smyc} into pL5
7	pML1357_dI_Fw	CCGCTCGAGCCGGCCGTGCGGAATTAAG (*XhoI*)<br>Removes *Giles integrase* from pML1357
8	pML1357_dI_Rv	CCCTCGAGGGTACCCGATACCGTCGG (*XhoI*)<br>Removes *Giles integrase* from pML1357
9	SCS_Fw	AAAACTGCAGTACCTGGGCCCAACTTGCTAGCCGAGAATGGGAG ACCCTGCACCACG (*PstI*, *ApaI*, *BmtI*)<br>Introduces second cloning site into pML1357dI
10	SCS_Rv	AAAACTGCAGACGCTAGATCTCGTTCCCTAGGCCATCTTCTACCA CGACCGCATCC (*PstI*, *BglII*, *AvrII*)<br>Introduces second cloning site into pML1357dI
11	Integrase_Fw	CTAGTCTAGACACGGCCTGTCCGCACCG (*XbaI*)<br>Transfers *Giles integrase* into pUC19
12	Integrase_Rv	CCCAAGCTTGGAAGCCGACGGTATCGGGTACC (*HindIII*)<br>Transfers *Giles integrase* into pUC19
13	NcoI_His6_1	CATGAGCGGTCACCATCACCATCACCACGGCGGTAGCGC<br>Introduces 6x-His tag downstream the *smyc* promoter
14	NcoI_His6_2	CATGGCGCTACCGCCGTGGTGATGGTGATGGTGACCGCT<br>Introduces 6x-His tag downstream the *smyc* promoter
15	eGFP_Fw	GTGTCCATGGTGAGCAAGGGCGAGGAG (*NcoI*)<br>Amplifies *eGFP*, *mClover3*, *mEmerald*, *mT-Sapphire*, and *mTurquoise2*
16	eGFP_Rv	GTGTAAGCTTTTACTTGTACAGCTCGTC (*HindIII*)<br>Amplifies *eGFP*, *mEmerald*, *mT-Sapphire*, *mWasabi*, *mRuby3*, and *mTFP1*

17	mWasabi_Fw	GTGTTCATGAGCGTGAGCAAGGGCGAGGAG (*BspHI*)<br>Amplifies *mWasabi*
18	mClover3_Rv	GTGTAAGCTTTCATTTGTATAGTTCATCCATGCCATGTGTAATCCC GGCGGCGGTCACGAACTC (*HindIII*)<br>Amplifies *mClover3*
19	ZsGreen_Fw	GTGTGCCATGGCCCAGTCCAAGCAC (*NcoI*)<br>Amplifies *ZsGreen*
20	ZsGreen_Rv	GTGTAAGCTTTCAGGGCAAGGCGGAGCC (*HindIII*)<br>Amplifies *ZsGreen*
21	mScarlet_Fw	GTGTGCCATGGTGAGCAAGGGCG (*NcoI*)<br>Amplifies *mScarlet*, and *COmScarlet*
22	mCherry_Fw	GTGTGACATGTCTGTCTCGAAGGGC (*PciI*)<br>Amplifies *mCherry*
23	mCherry_Rv	GCGCAAGCTTTCACTTGTACAGCTCGTC (*HindIII*)<br>Amplifies *mCherry*, *mScarlet*, and *mTurquoise2*
24	mRuby3_Fw	CTCTCCATGGTGTCTAAGGGCGAAG (*NcoI*)<br>Amplifies *mRuby3*
25	TagRFP_Fw	GTGTCCATGGCTCTGAGCTGATTAAGG (*NcoI*)<br>Amplifies *TagRFP*
26	TagRFP_Rv	GCGCAAGCTTCTAGAGCTTGCATGCCTG (*HindIII*)<br>Amplifies *TagRFP*
27	mTagBFP2_Fw	GTGGTCCATGGGCAGCGAGCTGATTAAGGAG (*NcoI*)<br>Amplifies *mTagBFP2*, and *mKate2*
28	mTagBFP2_Rv	GCCACAAGCTTTTAATTAAGCTTGTGCCCCAGTTTGC (*HindIII*)<br>Amplifies *mTagBFP2*
29	Ypet_Fw	CTCTCCATGGTGAGCAAAGGCGAAG (*NcoI*)<br>Amplifies *Ypet*
30	Ypet_Rv	GCGCAAGCTTTTACTTATAGAGCTCGTTC (*HindIII*)<br>Amplifies *Ypet*
31	CyOFP1_Fw	CTCTCCATGGTGAGCAAGGGCGAGG (*NcoI*)<br>Amplifies *CyOFP*
32	CyOFP1_Rv	GCGCAAGCTTTTACTTGTACAGCTCGTCC (*HindIII*)<br>Amplifies *CyOFP*
33	mPapaya_Fw	GCGCCCATGGTGAGCAAGGG (*NcoI*)<br>Amplifies *mPapaya*
34	mPapaya_Rv	GCGCAAGCTTTTACTTGTACAGCTCGTC (*HindIII*)<br>Amplifies *mPapaya*

35	mTFP1_Fw	CTCTTCATGAGTGTGAGCAAGGGCGAG (*BspHI*)<br>Amplifies *mTFP1*
36	mKate2_Rv	GTGTGAAGCTTTCATCTGTGCCCCAGTTTGC (*HindIII*)<br>Amplifies *mKate2*
37	mWasabi_dN_Fw	CCGACATCTCGATGGAGGAGG<br>Introduces point mutation to delete *mWasabi*'s internal *NcoI* site
38	mWasabi_dN_Rv	ACTTCACCTTCACGATGC<br>Introduces point mutation to delete *mWasabi*'s internal *NcoI* site
39	mWasabi_Fw2	CATGCCATGGGCAGCGTGAGCAAGGGCGAG (*NcoI*)<br>Amplifies *mWasabi dN*
40	mWasabi_Rv	CCCAAGCTTACTTGTACAGCTCGTCCATGCCGTC (*HindIII*)<br>Amplifies *mWasabi dN*
41	COmWasabi_Fw	GTGTTCATGAGCAGCGTGAGCAAGGGCGAG (*BspHI*)<br>Amplifies *COmWasabi*
42	COmWasabi_Rv	CCATCGATTACTTGTACAGCTCGTCCATGCCGTC (*ClaI*)<br>Amplifies *COmWasabi*
43	RBS_His_COmWasabi_Fw	CCCAAGCTTGCTTAATTAAGGAGGTATCTCCATGAGCGG (*HindIII*)<br>Transfers *COmWasabi* downstream *mWasabi dN* and *COmTagBFP2* downstream of *mTagBFP2*
44	COmScarlet_Rv	GTGTAAGCTTTCACTTATACAATTCATC (*HindIII*)<br>Amplifies *COmScarlet*
45	RBS_His_COmScarlet_Fw	GTGTAAGCTTTCCGCATGCTAATTAAG (*HindIII*)<br>Amplifies RBS *HisCOmScarlet*
46	mRuby3_triple_Fw	GTGTAACGTTTTAATTAAGGAGGTATCTCC (*AclI*)<br>Transfers *mRuby3* downstream of double red
47	mRuby3_triple_Rv	CCATCGATAAGCTTTTACTTGTAC (*ClaI*)
48	P_{G13}_Fw	GTGCGTCTAGAGATCGCCACTAGCGCCG (*XbaI*)<br>Amplifies *G13* promoter from pCHERRY10
49	P_{G13}_Rv	GCGCCCATGGAGATACCTCCTTCGGTTACCAAGCGTGC (*NcoI*)<br>Amplifies *G13* promoter from pCHERRY10
50	P_{msp12}_Fw	GGGCCACCGGTGCTCTAGAACTAGTGGATC (*XbaI*)<br>Amplifies *msp12* promoter from pTEC16
51	P_{msp12}_Rv	GCGCCCATGGAGATACCTCCTCGTCACTCCATGCTAAC (*NcoI*)<br>Amplifies *msp12* promoter from pTEC16
52	P_{MOP}_Fw	GTGTACTAGTCGGCTCTAGCGCCGATGGTAGTG (*SpeI*)<br>Amplifies P_{MOP} from pMH109
53	P_{MOP}_Rv	GTGTCCATGGTGAATCCTCCTGTCGACATCG (*NcoI*)<br>Amplifies P_{MOP} from pMH109

54	P_{left}_Fw	GCGCTCTAGACGATGATAAGCGGTCAAAC (*XbaI*)<br>Amplifies *left* promoter from synthetic DNA sequence
55	P_{left}_Rv	GTGTCCATGGACGATCTCCCTTTCCCGTC (*NcoI*)<br>Amplifies *left* promoter from synthetic DNA sequence
56	P_{left}_Fw2	GTACCAGATCTTTAAATCTAGACGATGATAAGCGGTCAAAC<br>Amplifies *left* promoter for fusion of P_{left} with the RBS of P_{smyc}
57	P_{left}_Rv2	CGGGCGGCGAATCTCTCTTCACCGGCACCGGCACGATCC<br>Amplifies *left* promoter for fusion of P_{left} with the RBS of P_{smyc}
58	Triple red_Fw	CAATCGTGCGCAGAATGGGGCCCATGGTGAGCAAGGGCGAGGCA<br>GTGATCAAGGAGTTC (contains internal *ApaI*)<br>Amplifies triple red to clone downstream of $P_{np2390c}$
59	Triple red_Rv	GGCGGGCTCAATTGAGCAGATCTTTACTTGTACAGCTCGTCCATG<br>CCACCACCAAGG (contains internal *BglII*)<br>Amplifies triple red to clone downstream of $P_{np2390c}$
60	$P_{np2395A}$_Fw	GTGTGCTAGCGGCCGGTCTGCTGATCAAGACTGC (*BmtI*)<br>Transfers His-tagged native promoter of *rv2395A* into *BmtI*/*ApaI* sites
61	$P_{np2395A}$ His_Rv	GTGTGGGCCCGCTACCGCCGTGGTGATGGTGATGGTGACCGCTCA<br>TCTCTGTCCCCCTTCCGAG<br>Transfers His-tagged native promoter of *rv2395A* into *BmtI*/*ApaI* sites
62	P_{np1535}_Fw	CTAGTCTAGAGCAAAGGCTGCCAAATACATGG (*XbaI*)<br>Amplifies *np1535* from genomic DNA
63	P_{np1535}_Rv	CATGCCATGGTCGCCTCCTAGACGGAG (*NcoI*)<br>Amplifies *np1535* from genomic DNA
64	P_{np1806}_Fw	CTAGTCTAGAAGGGTGTTCGGGTTGCGCTGG (*XbaI*)<br>Amplifies *np1806* from genomic DNA
65	P_{np1806}_Rv	CATGCCATGGGTCTTCTCCTGACAGACG (*NcoI*)<br>Amplifies *np1806* from genomic DNA
66	pL5 P_{smyc}_Fw	GGGCAGCGTGAGCAAGG<br>Constructs $P_{smyc1806}$
67	pL5 P_{smyc}_Rv	CTCCCAGAGCCTACTCGACCG<br>Constructs $P_{smyc1806}$
68	$P_{smyc1806}$_Fw	GAGTAGGCTCTGGGAGGCAAGCACCTCGCTAGGTGAGGCGTCTG<br>Constructs $P_{smyc1806}$
69	$P_{smyc1806}$_Rv	CCTTGCTCACGCTGCCCATGGGTCTTCTCCTGACAGACGACCG<br>Constructs $P_{smyc1806}$
70	pL5 P_{left}_Fw	AGCGGTCACCATCACCATCACCACG<br>Constructs $P_{left1806}$

71	pL5 P_{left}_Rv	GGTGACACAAGAATGCACTACTGGCC<br>Constructs $P_{left1806}$
72	$P_{left1806}$_Fw	GCATTCTTGTGTCACCGCAAGCACCTCGCTAGGTGAGGC<br>Constructs $P_{left1806}$
73	$P_{left1806}$_Rv	GGTGATGGTGACCGCTCATGGGTCTTCTCCTGACAGACGAC<br>Constructs $P_{left1806}$
74	COmTagBFP2_Fw	GTGTTCATGAGCTCGGAGCTGATCAAGG (*BspHI*)<br>Transfer *COmTagBFP2* downstream P_{smyc}-His
75	COmTagBFP2_Rv	CCATCGATTAGTTCAGCTTGTGGCCCAGC (*ClaI*)<br>Transfer *COmTagBFP2* downstream P_{smyc}-His

Table S5. Sequences of promoters used in this study. Known or predicted -35, -10 and TSS sites of the promoters are bolded and underlined; start codons are shown in bold; RBS are bolded and in red; Nucleotides encoding the His tag are depicted in blue; the RBS region of P_{smyc}, which was fused to P_{left} is highlighted in grey; the dotted line represents the promoter regions, which were fused to the riboswitch; and the sequence homology of the riboswitches upstream of *rv1535* and *rv1806* is highlighted in yellow.

Promoter	Sequence
P_{smyc}	GGATCGTCGGCACCGTCACGGCCGTGGGAGGCGGCACGATCCGCGACGTGATGAT<br>CGGCCGCATCCCCACGGTGCTGCGCAGTGAGCTCTACGCCATCCCGGCGTTGATCT<br>GTGCGTTCGCACGCACAGGCCCGGTGTGAGAAGGGTCTCTGACGAGCGGGAGAAC<br>CCACCCGGGGTGGGCGAGT**TTGTCC**TGCGTGTGCTCGGTCGAG**TAGGCT**CTGGG**A**<br>**GT**ACCCGTGTGTACGACCAGCACGGCATACATCATTTCGACGCCGAGAGATTCGCC<br>GCCCGAAATGAGCACGATCCGCATGCTTAATTA**AGGAGG**TATCTCC**ATG**
P_{smyc}-*His*	GGATCGTCGGCACCGTCACGGCCGTGGGAGGCGGCACGATCCGCGACGTGATGAT<br>CGGCCGCATCCCCACGGTGCTGCGCAGTGAGCTCTACGCCATCCCGGCGTTGATCT<br>GTGCGTTCGCACGCACAGGCCCGGTGTGAGAAGGGTCTCTGACGAGCGGGAGAAC<br>CCACCCGGGGTGGGCGAGT**TTGTCC**TGCGTGTGCTCGGTCGAG**TAGGCT**CTGGG**A**<br>**GT**ACCCGTGTGTACGACCAGCACGGCATACATCATTTCGACGCCGAGAGATTCGCC<br>GCCCGAAATGAGCACGATCCGCATGCTTAATTA**AGGAGG**TATCTCC**ATG**AGCGGT<br>**CACCATCACCATCACCAC**GGCGGTAGCGCC**ATG**
P_{hsp60}	CGGTGACCACAACGCGCCCGCTTTGATCGGGGACGTCTGCGGCCGACCATTTACGG<br>GTCTTGTTGTCGTTGGCGGTCATGGGCCGAACATACTCACCCGGATCGGAGGGCCG<br>AGGACAAGGTCGAACGAGGGGCATGACCCGGTGCGGGGCTTC**TTGCAC**TCGGCAT<br>AGGCGAGTGC**TAAGAA**TAACGT**TGG**CACTCGCGACCGGTGAGTGCTAGGTCGGGA<br>CGGTGAGGCCAGGCCCGTCGTCGCAGCGAGTGGCAGCGAGGACAACTTGAGCCGT<br>CCGTCGCGGGCACTGCGCCCGGCCAGCGTAAGTAGCGGGGTTGCCGTCACCCGGTG<br>ACCCCCGGTTTCATCCCCGATC**CGGAGG**AATCACTTCGCC**ATG**
P_{G13}	GATCGCCACTAGCGCCGCGGTCGGAATCAGCGCACTGGCCGCCGGAGCGGTCAGC<br>TACCTGTTGCTGCGCCGCTTCGCTTTCTAGCACCGACGTCCCGGCGCCAGCAGCTAC<br>CTCTCGAGCAAACGTTTTGCGCGCCCGAAAAATAAGTCGTTGCAGAACTTTCATGA<br>ATTAGGCC**TTGCTG**CGCCCAGGCTCCAGTAG**TAGAAA**TGGAG**TCA**CGGCAGCCCG<br>GTGAAGCCAAGGTCGAACCGGAAGAGAAGGTTCGTCCTCCCGACCCGGGCACCCA<br>GCACGGCCCCCGGAACCCACGCGGAGTCATAGCCGCGATAATGGCAGAAGTGTTG<br>CGGGCCTGCGTAATTGCGAAATTCAGATGGTGCCGACGGCCCTTTGGGTGGGGCTG<br>CAGCCAGAAGGGTCGCAAAAGCGCCGAGGCCAACCCACGCAGCCACAAATGCACG<br>CTTGGTAACCGA**AGGAGG**TATCTCC**ATG**

P_{msp12}	ACTAGTGGATCTGACCCGCTCCACAACCGCTGAGCAGGGTGGCAGTCAGAGCGAC<br>CAGGCCGACCGATACGAGGCGGATTTTGAACAGGTTCACTCGAAGCCTCCCGCTGG<br>GTTCGGAGCATCGTCGATCACATGACGGCGTGCTGACACCGATTCGACTATGCACA<br>GTAGTAGGTAGCCGCCGCGGACGGTAGCGCAGGCGGCCTCGGCGCCGTCGTGGCG<br>TCGCGATGGTTGGCGCCAGACCCGCCTGATCGGCGTCCCGACCAGGGCCGCCGGG<br>GTGGTGAGCCTCGGCTTGGTCCAGCCGTAGTGAGACGGGATGTCTTCCCGGTTGTC<br>GGGTTTGGGTGGGCGGTCACATGTTCGCTGGCTGTGGGCGTGTCGGGGGTCGTCGA<br>AGCGGCCGCGAACAGGGCTGGACGATGCACGTCTTGCACCTCCTGTCAACCCCCAA<br>TGTTGAGCCGCGATGCCCCTGACCTGCACCGTTGGACCGCAGTCGTTAGGGGGGCG<br>TGTTAGCATGGAGTGACG**AGGAGG**TATCTCC**ATG**
P_{MOP}	GGCTCTAGCGCCGATGGTAGTGTGGGGTCTCCCCATGCGAGAGTAGGGAACTGCCA<br>GGCATCAAATAAAACGAAAGGCTCAGTCGAAAGGCTGGGCCTTTCGTTTTATCTGT<br>TGTTTGTCGGTGAACGCTCTCCTGAGTAGGACAAATCCGCCGGGAGCGGATTTGAA<br>CGTTGCGAAGCAACGGCCCGGAGGGTGGCGGGCAGGACGCCCGCCATAAACTGCC<br>AGGCATCAAATTAAGCAGAAGGCCATCCTGACGGATGGCCTTTTTGCGTTTCTACA<br>AACTCTTCCTGTCGTCATATCTAGACCAGGCTTGACACTTTATGCTTCCGGCTCGTA<br>TAATGTGTGGAATTGTGAGCGCTCACAATTCGGATCCAGCGATGTCGAC**AGGAGG**<br>ATTCACC**ATG**
P_{left}	CGATGATAAGCGGTCAAACATGAGAATTCGCGGCCGCATAATACGACTCACTATA<br>GGGATCTTAATTAAGGCGCCTCATGTTCTTTCCTGCGTTATCCCCTGATTCTGTGGA<br>TAACCGTATTACCGCCTTTGAGTGAGCTGATACCGCTCGCCGCAGCCGAACGACCG<br>AGCGCAGCGAGTCAGTGAGCGAGGAAGCGGAAGAGCGCCCAATACGCAAACCGC<br>CTCTCCCAGATCTGATATCGCTAGAGGAAACAGCTATGACCATGATTACGCCAAGC<br>TTGCATGCCTGCAGCTAGGGCACCAATTTGCGATTAGGGC**TTGACA**GCCACCCGGC<br>CAGTAGTG**CATTCT**TGTGT**CAC**CGCAGCAGCAAGGCGGTAGGCGGATCCGAGAGG<br>ATCGTGCCGGTGCCGGTGAAAATCCGGCGGCAAGATTCTCCGGTTTGACAGCCACC<br>CGGTTATCGGGTAAGCTGCAAGCATCACCAACTTGGACGGGAA**AGGGAGA**TCGTC<br>C**ATG**
P_{Left*}	CGATGATAAGCGGTCAAACATGAGAATTCGCGGCCGCATAATACGACTCACTATA<br>GGGATCTTAATTAAGGCGCCTCATGTTCTTTCCTGCGTTATCCCCTGATTCTGTGGA<br>TAACCGTATTACCGCCTTTGAGTGAGCTGATACCGCTCGCCGCAGCCGAACGACCG<br>AGCGCAGCGAGTCAGTGAGCGAGGAAGCGGAAGAGCGCCCAATACGCAAACCGC<br>CTCTCCCAGATCTGATATCGCTAGAGGAAACAGCTATGACCATGATTACGCCAAGC<br>TTGCATGCCTGCAGCTAGGGCACCAATTTGCGATTAGGGC**TTGACA**GCCACCCGGC<br>CAGTAGTG**CATTCT**TGTGT**CAC**CGCAGCAGCAAGGCGGTAGGCGGATCCGAGAGG<br>ATCGTGCCGGTGCCGGTGAAGAGAGATTCGCCGCCCGAAATGAGCACGATCCGCA<br>TGCTTAATTA**AGGAGG**TATCTCC**ATG**
P_{left*}-*His*	CGATGATAAGCGGTCAAACATGAGAATTCGCGGCCGCATAATACGACTCACTATA<br>GGGATCTTAATTAAGGCGCCTCATGTTCTTTCCTGCGTTATCCCCTGATTCTGTGGA<br>TAACCGTATTACCGCCTTTGAGTGAGCTGATACCGCTCGCCGCAGCCGAACGACCG<br>AGCGCAGCGAGTCAGTGAGCGAGGAAGCGGAAGAGCGCCCAATACGCAAACCGC<br>CTCTCCCAGATCTGATATCGCTAGAGGAAACAGCTATGACCATGATTACGCCAAGC<br>TTGCATGCCTGCAGCTAGGGCACCAATTTGCGATTAGGGC**TTGACA**GCCACCCGGC<br>CAGTAGTG**CATTCT**TGTGT**CAC**CGCAGCAGCAAGGCGGTAGGCGGATCCGAGAGG<br>ATCGTGCCGGTGCCGGTGAAGAGAGATTCGCCGCCCGAAATGAGCACGATCCGCA<br>TGCTTAATTA**AGGAGG**TATCTCC**ATG**AGCGGT**CACCATCACCATCACCAC**GGCG<br>GTAGCGCC**ATG**
$P_{np2395A}$-*His*	GGCCGGTCTGCTGATCAAGACTGCGTATGGTCCGGCCACCGGCTCGCAGATTCCGG<br>CCCTGGTGGCCTACACCGTGTTTACCGCTGCATTGGTCTTCGGCGTGGCGACTATTT<br>CCAACGACAATCTGCAGGACCTCAAAACCGGCCAACTCGTCGGCGCTACCCCATGG<br>AAGCAGCAGGTTGCACTGATCATCGGCGTGCTCGTCGGGTCGGTGGTGATGGCGCC

GATCCTGCAGCTGATGCAGGCTGGATTCGGGTTCCAGGGGGCGCCGGGCGCAACG
GCCAACGCATTGGCCGCCCCGCAAGCCGCGCTCATGTCCGCGCTGGCCAAGGGAGT
ATTTGGTGGCTCGCTGAACTGGTCGCTGGTCGGTGTAGGGGCCTTGACCGGCGTGA
TAGCGGTCGCGCTCGACGAGACACTGGCCAAGACGACAACCAACCTTCGGCTGCC
GCCACTAGCGGTGGGTATGGGTATGTACCTGTCGGCCGCACTGACGCTGATGATCC
CGATCGGCGCATTCCTCGGGCGGATCTATGACTCCTGGGCGCGGTGGTCTGGGGAT
GACGACGAGCGCAAGAAACGGTTGGGCGTCATGCTCGCGACGGGCCTGATTGTGG
GCGAAAGCCTATACGGGGTGCTCTTTGCCGTCATCGTCGCGACAACTGGCAAAGAG
GAGCCGCTGGCCATGGTCGGCGACGGATTCAGGTTTGCCTCCCAGCCGCTGGGAGC
CATCGTCTTTGCCGGCCTCCTCGCTTGGCTCTACCAGCGCACCCGGGTCACAGCGTC
GTACCGGCTGGCAGCGCCGGCCGGCAGCTCCAAGCCACTGCCCGATTTGCCTGGGT
AACCGCATTGCGCCCGAGGGGTCCGGCTTTTCACAGCAACTTCACGGTTGACATCC
ACCTTGGCTCGCAGCTCTGCGAGGCAGCCTGAGGTGACAAAGCCGGCGGCCCGAC
ACATGCAGCCGAGTTGGCTGGCTCGGA**AGGGGG**ACAGAG**ATG**AGCGGT**CACCAT**
CACCATCACCACGGCGGTAGCGGGCCC**ATG**

P_{np1535}-*His*

GCAAAGGCTGCCAAATACATGGTGAACGCGTAAGGATTCGCGACACCCGCCCGGA
TCA**CGTTGAC**CGAGACGGGTAGGTCGTG**CATGAT**CGGTC**CGG**TAAGCACCTCGTT
AGGTGAGGCGGCTACACGAACATAGGCCACTGACCCCGAACGTCGAGAGACGCCC
CGGGTCAGGACAGCTCTTCCCGGCTTAAGGGTTGAGCCCAGGTGGCTTCCGGCTTA
CCGGACACGTCGTGTGGTGCCGAAGCTCTGACGAGAGGGGTGCGGATTTCCGGCA
GTTGCCGGCATCTCTGTACTCCTGTGACGCGCTTTATCGTGCGGACAACCGTACGTG
TCGTGGCCGTGAGGAGGTGAGGGACGCATGAGTTCCGGTGACAGTCCGGACCGAT
ATCCGGGCTCTGTTTCGTCCCGATCCGGTTTCCGGCGCGACGTTTTGCGCTGAGTCG
TCAAACCAAGATCAGCCTTCTTGGATCGGAACCGCTACGGGACGGGACCAACTCG
GTTCAGTCCATATGTGCTCGTTTTGATTTCCGTCCTCGCTTGCAACTCCGTCT**AGGA**
GGCGACC**ATG**AGCGGT**CACCATCACCATCACCAC**GGCGGTAGCGCC**ATG**

P_{np1806}-*His*

AGGGTGTTCGGGTTGCGCTGGACCCTGAAGGTCGTCTGCTGACCGGCGTTGTCTGC
TCGCTGGCTAACAGCCGATCTTGATAGCCTCCGGGGCATCGGATGAGTCAAGCCGT
TG**GGTTGAC**GCGCGTCGCTACGAGTGT**CACGAT**TACCCT**TGC**AAGCACCTCGCTA
GGTGAGGCGTCTGCGCGGATATAGGCCACTGACCTCGAACGTCGAAAGACGCCCA
GGGTCAGGACAGCTCTTCCCGGCTTAAGGGTTGAGCCCAAGTGGCTTCCGGCTGGA
CCGGCCGGATACGCCGTGTGGTGCCAAAGCTCTGACGAGAGGGGTGCCGAGTTCG
GTGGTCTGCTGGGCTGTCATCCCTTTGTGCTGTGCATCGGCATCCCCGTGTGCCCCG
GCCGTGAGGAGGTGAGAGCGAAATGAGTCCCGGCGATAGTCCGTATCCGAGATCG
ACGACCGTTTCGTTCCGATCCGACCCCGGCGCCGTTTTCGCACTCTGAATCGGCCTT
CCGGTTCGAAATCCGTTATTTCGCAAGCTCGTTGCTTCGCGGCCTTGTGTGAGTGAC
GTTCACGGGAAGTAGCCACGACAGAAGCGGTCATAGGCCTCCGGGTTCGGTCGTCT
GTC**AGGAGA**AGACCC**ATG**AGCGGT**CACCATCACCATCACCAC**GGCGGTAGCGCC
ATG

$P_{smyc1806}$-*His*

GGATCGTCGGCACCGTCACGGCCGTGGGAGGCGGCACGATCCGCGACGTGATGAT
CGGCCGCATCCCCACGGTGCTGCGCAGTGAGCTCTACGCCATCCCGGCGTTGATCT
GTGCGTTCGCACGCACAGGCCCGGTGTGAGAAGGGTCTCTGACGAGCGGGAGAAC
CCACCCGGGGTGGGCGAGT**TTGTCC**TGCGTGTGCTCGGTCGAG**TAGGCT**CTGGG**A**
GGCAAGCACCTCGCTAGGTGAGGCGTCTGCGCGGATATAGGCCACTGACCTCGAA
CGTCGAAAGACGCCCAGGGTCAGGACAGCTCTTCCCGGCTTAAGGGTTGAGCCCA
AGTGGCTTCCGGCTGGACCGGCCGGATACGCCGTGTGGTGCCAAAGCTCTGACGAG
AGGGGTGCCGAGTTCGGTGGTCTGCTGGGCTGTCATCCCTTTGTGCTGTGCATCGG
CATCCCCGTGTGCCCCGGCCGTGAGGAGGTGAGAGCGAAATGAGTCCCGGCGATA
GTCCGTATCCGAGATCGACGACCGTTTCGTTCCGATCCGACCCCGGCGCCGTTTTCG
CACTCTGAATCGGCCTTCCGGTTCGAAATCCGTTATTTCGCAAGCTCGTTGCTTCGC
GGCCTTGTGTGAGTGACGTTCACGGGAAGTAGCCACGACAGAAGCGGTCATAGGC
CTCCGGGTTCGGTCGTCTGTC**AGGAGA**AGACCC**ATG**AGCGGT**CACCATCACCATC**
ACCACGGCGGTAGCGCC**ATG**

$P_{left1806}$-*His*	CGATGATAAGCGGTCAAACATGAGAATTCGCGGCCGCATAATACGACTCACTATA<br>GGGATCTTAATTAAGGCGCCTCATGTTCTTTCCTGCGTTATCCCCTGATTCTGTGGA<br>TAACCGTATTACCGCCTTTGAGTGAGCTGATACCGCTCGCCGCAGCCGAACGACCG<br>AGCGCAGCGAGTCAGTGAGCGAGGAAGCGGAAGAGCGCCCAATACGCAAACCGC<br>CTCTCCCAGATCTGATATCGCTAGAGGAAACAGCTATGACCATGATTACGCCAAGC<br>TTGCATGCCTGCAGCTAGGGCACCAATTTGCGATTAGGGC**TTGACA**GCCACCCGGC<br>CAGTAGTG**CATTCT**TGTGT**CAC**CGCAAGCACCTCGCTAGGTGAGGCGTCTGCGCG<br>GATATAGGCCACTGACCTCGAACGTCGAAAGACGCCCAGGGTCAGGACAGCTCTT<br>CCCGGCTTAAGGGTTGAGCCCAAGTGGCTTCCGGCTGGACCGGCCGGATACGCCGT<br>GTGGTGCCAAAGCTCTGACGAGAGGGGTGCCGAGTTCGGTGGTCTGCTGGGCTGTC<br>ATCCCTTTGTGCTGTGCATCGGCATCCCCGTGTGCCCCGGCCGTGAGGAGGTGAGA<br>GCGAAATGAGTCCCGGCGATAGTCCGTATCCGAGATCGACGACCGTTTCGTTCCGA<br>TCCGACCCCGGCGCCGTTTTCGCACTCTGAATCGGCCTTCCGGTTCGAAATCCGTTA<br>TTTCGCAAGCTCGTTGCTTCGCGGCCTTGTGTGAGTGACGTTCACGGAAGTAGCC<br>ACGACAGAAGCGGTCATAGGCCTCCGGGTTCGGTCGTCTGTC**AGGAGA**AGACCC**A**<br>**TG**AGCGGT**CACCATCACCATCACCAC**GGCGGTAGCGCC**ATG**

Table S6. Sequences of genes for fluorescent proteins used in this study.

Gene	Sequence
eGFP	**ATG**GTGAGCAAGGGCGAGGAGCTGTTCACCGGGGTGGTGCCCATCCTGGTCGAGC<br>TGGACGGCGACGTAAACGGCCACAAGTTCAGCGTGTCCGGCGAGGGCGAGGGCGA<br>TGCCACCTACGGCAAGCTGACCCTGAAGTTCATCTGCACCACCGGCAAGCTGCCCG<br>TGCCCTGGCCCACCCTCGTGACCACCCTGACCTACGGCGTGCAGTGCTTCAGCCGC<br>TACCCCGACCACATGAAGCAGCACGACTTCTTCAAGTCCGCCATGCCCGAAGGCTA<br>CGTCCAGGAGCGCACCATCTTCTTCAAGGACGACGGCAACTACAAGACCCGCGCC<br>GAGGTGAAGTTCGAGGGCGACACCCTGGTGAACCGCATCGAGCTGAAGGGCATCG<br>ACTTCAAGGAGGACGGCAACATCCTGGGGCACAAGCTGGAGTACAACTACAACAG<br>CCACAACGTCTATATCATGGCCGACAAGCAGAAGAACGGCATCAAGGTGAACTTC<br>AAGATCCGCCACAACATCGAGGACGGCAGCGTGCAGCTCGCCGACCACTACCAGC<br>AGAACACCCCCATCGGCGACGGCCCCGTGCTGCTGCCCGACAACCACTACCTGAGC<br>ACCCAGTCCGCCCTGAGCAAAGACCCCAACGAGAAGCGCGATCACATGGTCCTGC<br>TGGAGTTCGTGACCGCCGCCGGGATCACTCTCGGCATGGACGAGCTGTACAAGTAA
mClover3	**ATG**GTGAGCAAGGGCGAGGAGCTGTTCACCGGGGTGGTGCCCATCCTGGTCGAGC<br>TGGACGGCGACGTAAACGGCCACAAGTTCAGCGTCCGCGGCGAGGGCGAGGGCGA<br>TGCCACCAACGGCAAGCTGACCCTGAAGTTCATCTGCACCACCGGCAAGCTGCCCG<br>TGCCCTGGCCCACCCTCGTGACCACCTTCGGCTACGGCGTGGCCTGCTTCAGCCGCT<br>ACCCCGACCACATGAAGCAGCACGACTTCTTCAAGTCCGCCATGCCCGAAGGCTAC<br>GTCCAGGAGCGCACCATCTCTTTCAAGGACGACGGTACCTACAAGACCCGCGCCGA<br>GGTGAAGTTCGAGGGCGACACCCTGGTGAACCGCATCGAGCTGAAGGGCATCGAC<br>TTCAAGGAGGACGGCAACATCCTGGGGCACAAGCTGGAGTACAACTTCAACAGCC<br>ACTACGTCTATATCACGGCCGACAAGCAGAAGAACTGCATCAAGGCTAACTTCAA<br>GATCCGCCACAACGTTGAGGACGGCAGCGTGCAGCTCGCCGACCACTACCAGCAG<br>AACACCCCCATCGGCGACGGCCCCGTGCTGCTGCCCGACAACCACTACCTGAGCCA<br>TCAGTCCAAGCTGAGCAAAGACCCCAACGAGAAGCGCGATCACATGGTCCTGCTG<br>GAGTTCGTGACCGCCGCCGGGATTACACATGGCATGGATGAACTATACAAATGA

mEmerald	**ATG**GTGAGCAAGGGCGAGGAGCTGTTCACCGGGGTGGTGCCCATCCTGGTCGAGC<br>TGGACGGCGACGTAAACGGCCACAAGTTCAGCGTGTCCGGCGAGGGCGAGGGCGA<br>TGCCACCTACGGCAAGCTGACCCTGAAGTTCATCTGCACCACCGGCAAGCTGCCCG<br>TGCCCTGGCCCACCCTCGTGACCACCTTGACCTACGGCGTGCAGTGCTTCGCCCGCT<br>ACCCCGACCACATGAAGCAGCACGACTTCTTCAAGTCCGCCATGCCCGAAGGCTAC<br>GTCCAGGAGCGCACCATCTTCTTCAAGGACGACGGCAACTACAAGACCCGCGCCG<br>AGGTGAAGTTCGAGGGCGACACCCTGGTGAACCGCATCGAGCTGAAGGGCATCGA<br>CTTCAAGGAGGACGGCAACATCCTGGGGCACAAGCTGGAGTACAACTACAACAGC<br>CACAAGGTCTATATCACCGCCGACAAGCAGAAGAACGGCATCAAGGTGAACTTCA<br>AGACCCGCCACAACATCGAGGACGGCAGCGTGCAGCTCGCCGACCACTACCAGCA<br>GAACACCCCCATCGGCGACGGCCCCGTGCTGCTGCCCGACAACCACTACCTGAGCA<br>CCCAGTCCAAGCTGAGCAAAGACCCCAACGAGAAGCGCGATCACATGGTCCTGCT<br>GGAGTTCGTGACCGCCGCCGGGATCACTCTCGGCATGGACGAGCTGTACAAGTAA
mWasabi	**ATG**AGCGTGAGCAAGGGCGAGGAGACCACAATGGGCGTAATCAAGCCCGACATG<br>AAGATCAAGCTGAAGATGGAGGGCAACGTGAATGGCCACGCCTTCGTGATCGAGG<br>GCGAGGGCGAGGGCAAGCCCTACGACGGCACCAACACCATCAACCTGGAGGTGAA<br>GGAGGGAGCCCCCCTGCCCTTCTCCTACGACATTCTGACCACCGCGTTCAGTTACG<br>GCAACAGGGCCTTCACCAAGTACCCCGACGACATCCCCAACTACTTCAAGCAGTCC<br>TTCCCCGAGGGCTACTCTTGGGAGCGCACCATGACCTTCGAGGACAAGGGCATCGT<br>GAAGGTGAAGTCCGACATCTCCATGGAGGAGGACTCCTTCATCTACGAGATACACC<br>TCAAGGGCGAGAACTTCCCCCCCAACGGCCCCGTGATGCAGAAGGAGACCACCGG<br>CTGGGACGCCTCCACCGAGAGGATGTACGTGCGCGACGGCGTGCTGAAGGGCGAC<br>GTCAAGATGAAGCTGCTGCTGGAGGGCGGCGGCCACCACCGCGTTGACTTCAAGA<br>CCATCTACAGGGCCAAGAAGGCGGTGAAGCTGCCCGACTATCACTTTGTGGACCAC<br>CGCATCGAGATCCTGAACCACGACAAGGACTACAACAAGGTGACCGTTTACGAGA<br>TCGCCGTGGCCCGCAACTCCACCGACGGCATGGACGAGCTGTACAAGTAA
ZsGreen	**ATG**GCCCAGTCCAAGCACGGCCTGACCAAGGAGATGACCATGAAGTACCGCATGG<br>AGGGCTGCGTGGACGGCCACAAGTTCGTGATCACCGGCGAGGGCATCGGCTACCC<br>CTTCAAGGGCAAGCAGGCCATCAACCTGTGCGTGGTGGAGGGCGGCCCCTTGCCCT<br>TCGCCGAGGACATCTTGTCCGCCGCCTTCATGTACGGCAACCGCGTGTTCACCGAG<br>TACCCCCAGGACATCGTCGACTACTTCAAGAACTCCTGCCCCGCCGGCTACACCTG<br>GGACCGCTCCTTCCTGTTCGAGGACGGCGCCGTGTGCATCTGCAACGCCGACATCA<br>CCGTGAGCGTGGAGGAGAACTGCATGTACCACGAGTCCAAGTTCTACGGCGTGAA<br>CTTCCCCGCCGACGGCCCCGTGATGAAGAAGATGACCGACAACTGGGAGCCCTCCT<br>GCGAGAAGATCATCCCCGTGCCCAAGCAGGGCATCTTGAAGGGCGACGTGAGCAT<br>GTACCTGCTGCTGAAGGACGGTGGCCGCTTGCGCTGCCAGTTCGACACCGTGTACA<br>AGGCCAAGTCCGTGCCCCGCAAGATGCCCGACTGGCACTTCATCCAGCACAAGCTG<br>ACCCGCGAGGACCGCAGCGACGCCAAGAACCAGAAGTGGCACCTGACCGAGCACG<br>CCATCGCCTCCGGCTCCGCCTTGCCCTGA

mCherry	**ATG**TCTGTCTCGAAGGGCGAGGAGGACAACATGGCGATCATCAAGGAGTTCATGC<br>GCTTCAAGGTCCACATGGAGGGCTCGGTCAACGGCCACGAGTTCGAGATCGAGGG<br>CGAGGGCGAGGGCCGCCCGTACGAGGGCACCCAGACCGCCAAGCTGAAGGTCACC<br>AAGGGCGGCCCGCTGCCGTTCGCCTGGGACATCCTGTCGCCGCAGTTCATGTACGG<br>CAGCAAGGCCTACGTCAAGCACCCGGCCGACATCCCGGACTACCTGAAGCTGTCGT<br>TCCCGGAGGGCTTCAAGTGGGAGCGCGTCATGAACTTCGAGGACGGCGGCGTCGT<br>CACCGTCACCCAGGACTCGTCGCTGCAGGACGGCGAGTTCATCTACAAGGTCAAGC<br>TGCGGGGCACCAACTTCCCGTCGGACGGCCCGGTCATGCAGAAGAAGACCATGGG<br>CTGGGAGGCCTCGTCGGAGCGCATGTACCCGGAGGACGGCGCCCTGAAGGGCGAG<br>ATCAAGCAGCGGCTGAAGCTGAAGGACGGCGGCCACTACGACGCCGAGGTCAAGA<br>CCACCTACAAGGCCAAGAAGCCGGTCCAGCTGCCGGGCGCCTACAACGTGAACAT<br>CAAGCTGGACATCACCAGCCACAACGAGGACTACACCATCGTCGAGCAGTACGAG<br>CGCGCCGAGGGCCGCCACAGCACCGGCGGCATGGACGAGCTGTACAAGTGA
mRuby3	**ATG**GTGTCTAAGGGCGAAGAGCTGATCAAGGAAAATATGCGTATGAAGGTGGTCA<br>TGGAAGGTTCGGTCAACGGCCACCAATTCAAATGCACAGGTGAAGGAGAAGGCAG<br>ACCGTACGAGGGAGTGCAAACCATGAGGATCAAAGTCATCGAGGGAGGACCCCTG<br>CCATTTGCCTTTGACATTCTTGCCACGTCGTTCATGTATGGCAGCCGTACCTTTATC<br>AAGTACCCGGCCGACATCCCTGATTTCTTTAAACAGTCCTTTCCTGAGGGTTTTACT<br>TGGGAAAGAGTTACGAGATACGAAGATGGTGGAGTCGTCACCGTCACGCAGGACA<br>CCAGCCTTGAGGATGGCGAGCTCGTCTACAACGTCAAGGTCAGAGGGGTAAACTTT<br>CCCTCCAATGGTCCCGTGATGCAGAAGAAGACCAAGGGTTGGGAGCCTAATACAG<br>AGATGATGTATCCAGCAGATGGTGGTCTGAGAGGATACACTGACATCGCACTGAA<br>AGTTGATGGTGGTGGCCATCTGCACTGCAACTTCGTGACAACTTACAGGTCAAAAA<br>AGACCGTCGGGAACATCAAGATGCCCGGTGTCCATGCCGTTGATCACCGCCTGGAA<br>AGGATCGAGGAGAGTGACAATGAAACCTACGTAGTGCAAAGAGAAGTGGCAGTTG<br>CCAAATACAGCAACCTTGGTGGTGGCATGGACGAGCTGTACAAGTAA
TagRFP	**ATG**GCTCTGAGCTGATTAAGGAGAACATGCACATGAAGCTGTACATGGAGGGCAC<br>CGTGAACAACCACCACTTCAAGTGCACATCCGAGGGCGAAGGCAAGCCCTACGAG<br>GGCACCCAGACCATGAGAATCAAGGTGGTCGAGGGCGGCCCTTTACCCTTCGCCTT<br>CGACATCCTGGCTACCAGCTTCATGTACGGCAGCAGAACCTTCATCAACCACACCC<br>AGGGCATCCCCGACTTCTTTAAGCAGTCCTTCCCTGAGGGCTTCACATGGGAGAGA<br>GTCACCACATACGAAGACGGGGGCGTGCTGACCGCTACCCAGGACACCAGCTTAC<br>AGGACGGCTGCTTGATCTACAACGTCAAGATCAGAGGGGTGAACTTCCCATCCAAC<br>GGCCCTGTGATGCAGAAGAAAACATTAGGCTGGGAGGCCAACACCGAGATGCTGT<br>ACCCCGCTGACGGCGGCCTGGAAGGCAGAAGCGACATGGCCCTGAAGTTAGTGGG<br>CGGGGGCCACCTGATCTGCAACTTCAAGACCACATACAGATCCAAGAAACCCGCT<br>AAGAACTTGAAGATGCCCGGCGTCTACTATGTGGACCACAGACTGGAAAGAATCA<br>AGGAGGCCGACAAAGAGACCTACGTCGAGCAGCACGAGGTGGCTGTGGCCAGATA<br>CTGCGACCTGCCTAGCAAACTGGGGCACAAATAAATGTCCAGACCTGCAGGCATG<br>CAAGCTCTAG

mScarlet	**ATG**GTGAGCAAGGGCGAGGCAGTGATCAAGGAGTTCATGCGGTTCAAGGTGCACA<br>TGGAGGGCTCCATGAACGGCCACGAGTTCGAGATCGAGGGCGAGGGCGAGGGCCG<br>CCCCTACGAGGGCACCCAGACCGCCAAGCTGAAGGTGACCAAGGGTGGCCCCCTG<br>CCCTTCTCCTGGGACATCCTGTCCCCTCAGTTCATGTACGGCTCCAGGGCCTTCACC<br>AAGCACCCCGCCGACATCCCCGACTACTATAAGCAGTCCTTCCCCGAGGGCTTCAA<br>GTGGGAGCGCGTGATGAACTTCGAGGACGGCGGCGCCGTGACCGTGACCCAGGAC<br>ACCTCCCTGGAGGACGGCACCCTGATCTACAAGGTGAAGCTCCGCGGCACCAACTT<br>CCCTCCTGACGGCCCCGTAATGCAGAAGAAGACAATGGGCTGGGAAGCGTCCACC<br>GAGCGGTTGTACCCCGAGGACGGCGTGCTGAAGGGCGACATTAAGATGGCCCTGC<br>GCCTGAAGGACGGCGGCCGCTACCTGGCGGACTTCAAGACCACCTACAAGGCCAA<br>GAAGCCCGTGCAGATGCCCGGCGCCTACAACGTCGACCGCAAGTTGGACATCACCT<br>CCCACAACGAGGACTACACCGTGGTGGAACAGTACGAACGCTCCGAGGGCCGCCA<br>CTCCACCGGCGGCATGGACGAGCTGTACAAGTGA
mTagBFP2	**ATG**GGCAGCGAGCTGATTAAGGAGAACATGCACATGAAGCTGTACATGGAGGGCA<br>CCGTGGACAACCATCACTTCAAGTGCACATCCGAGGGCGAAGGCAAGCCCTACGA<br>GGGCACCCAGACCATGAGAATCAAGGTGGTCGAGGGCGGCCCTCTCCCCTTCGCCT<br>TCGACATCCTGGCTACTAGCTTCCTCTACGGCAGCAAGACCTTCATCAACCACACC<br>CAGGGCATCCCCGACTTCTTCAAGCAGTCCTTCCCTGAGGGCTTCACATGGGAGAG<br>AGTCACCACATACGAAGACGGGGGCGTGCTGACCGCTACCCAGGACACCAGCCTC<br>CAGGACGGCTGCCTCATCTACAACGTCAAGATCAGAGGGGTGAACTTCACATCCAA<br>CGGCCCTGTGATGCAGAAGAAAACACTCGGCTGGGAGGCCTTCACCGAGACGCTG<br>TACCCCGCTGACGGCGGCCTGGAAGGCAGAAACGACATGGCCCTGAAGCTCGTGG<br>GCGGGAGCCATCTGATCGCAAACGCCAAGACCACATATAGATCCAAGAAACCCGC<br>TAAGAACCTCAAGATGCCTGGCGTCTACTATGTGGACTACAGACTGGAAAGAATCA<br>AGGAGGCCAACAACGAGACCTACGTCGAGCAGCACGAGGTGGCAGTGGCCAGATA<br>CTGCGACCTCCCTAGCAAACTGGGGCACAAGCTCAATTAA
LSSmOrange	**ATG**AGCGTGAGCAAGGGCGAGGAGAATAACATGGCCATCATCAAGGAGTTCATGC<br>GCTTCAAGGTGCGCATGGAGGGCTCCGTGAACGGCCACGAGTTCGAGATCGAGGG<br>CGAGGGCGAGGGCCGCCCCTACGAGGGCTTTCAGACCGTTAAGCTGAAGGTGACC<br>AAGGGTGGCCCCCTGCCCTTCGCCTGGGACATCTTGTCCCCTCAGTTCACCTACGGC<br>TCCAAGGCCTACGTGAAGCACCCCGCCGACATCCCCGACTACCTCAAGCTGTCCTT<br>CCCCGAGGGCTTCAAGTGGGAGCGCGTGATGAACTTCGAGGACGGCGGCGTGGTG<br>ACCGTGACTCAGGACTCCTCCCTGCAGGACGGCGAGTTCATCTACAAGGTGAAGCT<br>GCGCGGCACCAACTTCCCCTCCGACGGCCCCGTAATGCAGAAGAAGACCATGGGC<br>ATGGAGGCCTCCTCCGAGCGGATGTACCCCGAGGACGGCGCCCTGAAGGGCGAGG<br>ACAAGCTCAGGCTGAAGCTGAAGGACGGCGGCCACTACACCTCCGAGGTCAAGAC<br>CACCTACAAGGCCAAGAAGCCCGTGCAGTTGCCCGGCGCCTACATCGTCGACATCA<br>AGTTGGACATCACCTCCCACAACGAGGACTACACCATCGTGGAACAGTACGAACG<br>CGCCGAGGGCCGCCACTCCACCGGCGGCATGGACGAGCTGTACAAGTAA
mPapaya	**ATG**GTGAGCAAGGGCGAGGGGCAATCGAAGCATGGACTAAAGGAGGAGATGACG<br>GTGAAGTACCACATGGAAGGTTGCGTTAATGGTCACAAATTTGTCATTACTGGAGA<br>GGGCATTGGAAACCCTTTTAAGGGTAAGCAAACCGCAAATTTGTGTGTGATAGAAG<br>GAGGCCCGCTGCCGTTCTCGGAGGACATTTTAAGTCCGGGTTTTAAATATGGTGAC<br>CGGATTTTCACAGAGTACCCACAGGATATTGTAGATTACTTCAAGAACTCATGTCC<br>AGCGGGCTATACGTGGGAAAGGAGCTACCTCTTTGAGGACGGAGCGGTCTGTCGA<br>TGCAACGTGGACATAACAGTCTCTGAAAAGGAGAACTGCATCTATCACAAAAGTA<br>TCTTCAGAGGGGTGAATTTTCCCGCCGACGGCCCCGTAATGAAGAAAATGACCACT<br>AATTGGGAAGCTAGTACCGAGAAAATTGTGCCTGTTCCAAAGCAAGGGATATTAA<br>AGGGAAAGGTCAAAATGTGCCTGTTGCTGAAGGATGGCGGTCGTTATCATTGCCAG<br>TTTGATACGGTATATAAAGCTAAATCAGTGCCCTCCAAGATGCCAGAATGGCATTT<br>TATACAGCATAAGCTCCTTAGGGAGGACCGCTCTGATGCTAAAAACCAGAAATGG<br>CAATTAACAGAACATGCAATCGCAGGCATGGACGAGCTGTACAAGTAA

mCardinal

ATGGTGAGCAAGGGCGAGGAGCTGATCAAGGAGAACATGCCCATGAAGCTGTACA
TGGAAGGCACCGTGAACAACCACCACTTCAAGTGCACCACCGAAGGGGAGGGCAA
GCCCTACGAGGGCACCCAGACCCAGAGGATTAAGGTGGTGGAGGGAGGCCCCCTG
CCGTTCGCATTCGACATCCTGGCCACCTGCTTTATGTACGGGAGCAAGACCTTCATC
AAGCACCCCAAGGGCATCCCCGATTTCTTTAAGCAGTCCTTCCCTGAGGGCTTCAC
ATGGGAGAGAGTCACCACATACGAAGACGGGGGCGTGCTTACCGTTACCCAGGAC
ACCAGCCTCCAGGACGGCTGCTTGATCTACAACGTCAAGCTCAGAGGGGTGAACTT
CCCATCCAACGGCCCTGTGATGCAGAAGAAAACACTCGGCTGGGAGGCCACCACC
GAGACCCTGTACCCCGCTGACGGCGGCCTGGAAGGCAGATGCGACATGGCCCTGA
AGCTCGACGGCGGGGGCCACCTGCACTGCAACCTGAAGACCACATACAGATCCAA
GAAACCCGCTGGCAACCTCAAGATGCCCGGCGTCTACTTTGTGGACCGCAGACTGG
AAAGAATCAAGGAGGCCGACAATGAGACCTACGTCGAGCAGCACGAGGTGGCCGA
GGCCAGATACTGCGACCTCCCTAGCAAACTGGGGCACAAACTTAATGGCATGGAC
GAGCTGTACAAGTAA

COmWasabi

ATGAGCAGCGTGAGCAAGGGCGAGGAGACCACAATGGGCGTGATCAAGCCGGAC
ATGAAGATCAAGCTGAAGATGGAGGGCAACGTGAACGGCCACGCCTTCGTGATCG
AGGGCGAGGGCGAGGGCAAGCCGTACGACGGCACCAACACCATCAACCTGGAGGT
GAAGGAGGGCGCCCCGCTGCCGTTCTCGTACGACATCCTGACCACCGCCTTCTCGT
ACGGCAACCGCGCCTTCACCAAGTACCCGGACGACATCCCGAACTACTTCAAGCAG
TCGTTCCCGGAGGGCTACTCGTGGGAGCGCACCATGACCTTCGAGGACAAGGGCAT
CGTGAAGGTGAAGTCGGACATCTCGATGGAGGAGGACTCGTTCATCTACGAGATCC
ACCTGAAGGGCGAGAACTTCCCGCCGAACGGCCCGGTGATGCAGAAGGAGACCAC
CGGCTGGGACGCCTCGACCGAGCGCATGTACGTGCGCGACGGCGTGCTGAAGGGC
GACGTGAAGATGAAGCTGCTGCTGGAGGGCGGCGGCCACCACCGCGTGGACTTCA
AGACCATCTACCGCGCCAAGAAGGCCGTGAAGCTGCCGGACTACCACTTCGTGGA
CCACCGCATCGAGATCCTGAACCACGACAAGGACTACAACAAGGTGACCGTGTAC
GAGATCGCCGTGGCCCGCAACTCGACCGACGGCATGGACGAGCTGTACAAGTAA

COmScarlet

ATGGTGAGCAAGGGCGAGGCGGTGATTAAAGAGTTTATGCGTTTCAAAGTGCATA
TGGAAGGGTCGATGAACGGGCATGAGTTTGAAATCGAGGGGGAGGGCGAGGGCCG
TCCATACGAAGGGACGCAAACCGCGAAATTGAAGGTCACGAAAGGCGGCCCGCTC
CCGTTCTCGTGGGATATCCTCAGCCCACAATTCATGTACGGTTCGCGTGCGTTTACC
AAGCACCCCGCCGACATCCCGGACTACTACAAACAGAGCTTCCCCGAGGGGTTCA
AATGGGAACGTGTGATGAATTTTGAGGACGGGGGTGCGGTGACGGTCACCCAAGA
TACCTCGCTGGAAGACGGGACCCTCATCTATAAGGTCAAGTTGCGGGGGACGAATT
TCCCCCCCGACGGTCCCGTCATGCAAAAGAAAACGATGGGCTGGGAGGCCTCGAC
CGAGCGGCTGTATCCAGAAGATGGCGTGTTGAAGGGTGACATTAAAATGGCCCTG
CGGTTGAAAGATGGTGGGCGCTACTTGGCGGACTTCAAAACCACCTACAAAGCCA
AAAAACCCGTGCAAATGCCCGGGGCGTATAATGTCGACCGTAAGCTGGATATTAC
GAGCCATAACGAAGATTACACGGTGGTGGAACAATACGAACGCTCGGAGGGTCGT
CATTCCACCGGGGGGATGGATGAATTGTATAAGTGA

COmTagBFP2	**ATG**AGCTCGGAGCTGATCAAGGAGAACATGCACATGAAGCTGTACATGGAGGGCA<br>CCGTGGACAACCACCACTTCAAGTGCACCTCGGAGGGCGAGGGCAAGCCGTACGA<br>GGGCACCCAGACCATGCGCATCAAGGTGGTGGAGGGCGGCCCGCTGCCGTTCGCC<br>TTCGACATCCTGGCCACCTCGTTCCTGTACGGCTCGAAGACCTTCATCAACCACACC<br>CAGGGCATCCCGGACTTCTTCAAGCAGTCGTTCCCGGAGGGCTTCACCTGGGAGCG<br>CGTGACCACCTACGAGGACGGCGGCGTGCTGACCGCCACCCAGGACACCTCGCTG<br>CAGGACGGCTGCCTGATCTACAACGTGAAGATCCGCGGCGTGAACTTCACCTCGAA<br>CGGCCCGGTGATGCAGAAGAAGACCCTGGGCTGGGAGGCCTTCACCGAGACCCTG<br>TACCCGGCCGACGGCGGCCTGGAGGGCCGCAACGACATGGCCCTGAAGCTGGTGG<br>GCGGCTCGCACCTGATCGCCAACGCCAAGACCACCTACCGCTCGAAGAAGCCGGC<br>CAAGAACCTGAAGATGCCGGGCGTGTACTACGTGGACTACCGCCTGGAGCGCATC<br>AAGGAGGCCAACAACGAGACCTACGTGGAGCAGCACGAGGTGGCCGTGGCCCGCT<br>ACTGCGACCTGCCGTCGAAGCTGGGCCACAAGCTGAACTAA

4 References

Ai, H.W., Hazelwood, K.L., Davidson, M.W., and Campbell, R.E. (2008). Fluorescent protein FRET pairs for ratiometric imaging of dual biosensors. *Nat. Methods* 5, 401-403.

Ai, H.W., Henderson, J.N., Remington, S.J., and Campbell, R.E. (2006). Directed evolution of a monomeric, bright and photostable version of Clavularia cyan fluorescent protein: structural characterization and applications in fluorescence imaging. *Biochem. J.* 400, 531-540.

Bajar, B.T., Wang, E.S., Lam, A.J., Kim, B.B., Jacobs, C.L., Howe, E.S., et al. (2016). Improving brightness and photostability of green and red fluorescent proteins for live cell imaging and FRET reporting. *Sci. Rep.* 6:20889. doi: 10.1038/srep20889.

Balleza, E., Kim, J.M., and Cluzel, P. (2018). Systematic characterization of maturation time of fluorescent proteins in living cells. *Nat. Methods* 15, 47-51.

Carroll, P., Schreuder, L.J., Muwanguzi-Karugaba, J., Wiles, S., Robertson, B.D., Ripoll, J., et al. (2010). Sensitive detection of gene expression in mycobacteria under replicating and non-replicating conditions using optimized far-red reporters. *PLoS One* 5:e9823. doi: 10.1371/journal.pone.0009823.

Chan, K., Knaak, T., Satkamp, L., Humbert, O., Falkow, S., and Ramakrishnan, L. (2002). Complex pattern of *Mycobacterium marinum* gene expression during long-term granulomatous infection. *Proc. Natl. Acad. Sci. U S A* 99, 3920-3925.

Chu, J., Haynes, R.D., Corbel, S.Y., Li, P., González-González, E., Burg, J.S., et al. (2014). Non-invasive intravital imaging of cellular differentiation with a bright red-excitable fluorescent protein. *Nat. Methods* 11, 572-578.

Chu, J., Oh, Y., Sens, A., Ataie, N., Dana, H., Macklin, J.J., et al. (2016). A bright cyan-excitable orange fluorescent protein facilitates dual-emission microscopy and enhances bioluminescence imaging *in vivo*. *Nat. Biotechnol.* 34, 760-767.

George, K.M., Yuan, Y., Sherman, D.R., and Barry, C.E. 3rd (1995). The biosynthesis of cyclopropanated mycolic acids in *Mycobacterium tuberculosis*. Identification and functional analysis of CMAS-2. *J. Biol. Chem.* 270, 27292-27298.

Hermann, M., Stillhard, P., Wildner, H., Seruggia, D., Kapp, V., Sánchez-Iranzo, H., et al. (2014). Binary recombinase systems for high-resolution conditional mutagenesis. *Nucleic Acids Res.* 42, 3894-3907.

Hoi, H., Howe, E.S., Ding, Y., Zhang, W., Baird, M.A., Sell, B.R., et al. (2013). An engineered monomeric Zoanthus sp. yellow fluorescent protein. *Chem. Biol.* 20, 1296-1304.

Huff, J., Czyz, A., Landick, R., and Niederweis, M. (2010). Taking phage integration to the next level as a genetic tool for mycobacteria. *Gene* 468, 8-19.

Mastop, M., Bindels, D.S., Shaner, N.C., Postma, M., Gadella, T.W.J. Jr., and Goedhart, J. (2017). Characterization of a spectrally diverse set of fluorescent proteins as FRET acceptors for mTurquoise2. *Sci. Rep.* 7, 11999.

Nguyen, A.W., and Daugherty, P.S. (2005). Evolutionary optimization of fluorescent proteins for intracellular FRET. *Nat. Biotechnol.* 23, 355-360.

Norrander, J., Kempe, T., and Messing, J. (1983). Construction of improved M13 vectors using oligodeoxynucleotide-directed mutagenesis. *Gene* 26, 101-106.

Ó Gaora, P., Barnini, S., Hayward, C., Filley, E., Rool, G., Young, D., et al. (1997). Mycobacteria as Immunogens: Development of Expression Vectors for Use in Multiple Mycobacterial Species. *Med. Principles Pract.* 6, 91-96.

Shcherbakova, D.M., Hink, M.A., Joosen, L., Gadella, T.W., and Verkhusha, V.V. (2012). An orange fluorescent protein with a large Stokes shift for single-excitation multicolor FCCS and FRET imaging. *J. Am. Chem. Soc.* 134, 7913-7923.

Springer, B., Sander, P., Sedlacek, L., Ellrott, K., and Böttger, E.C. (2001). Instability and site-specific excision of integration-proficient mycobacteriophage L5 plasmids: development of stably maintained integrative vectors. *Int. J. Med. Microbiol.* 290, 669-75.

Stover, C.K., de la Cruz, V.F., Fuerst, T.R., Burlein, J.E., Benson, L.A., Bennett, L.T., et al. (1991). New use of BCG for recombinant vaccines. *Nature* 351, 456-460.

Subach, O.M., Cranfill, P.J., Davidson, M.W., and Verkhusha, V.V. (2011). An enhanced monomeric blue fluorescent protein with the high chemical stability of the chromophore. *PLoS One* 6:e28674. doi: 10.1371/journal.pone.0028674.

Takaki, K., Davis, J.M., Winglee, K., and Ramakrishnan, L. (2013). Evaluation of the pathogenesis and treatment of *Mycobacterium marinum* infection in zebrafish. *Nat. Protoc.* 8, 1114-1124.
